# Ferroelectric, Switchable Dielectric and Nonlinear Optical Properties in Inorganic–Organic Lead-Free 1D Hybrids Based on Bi(III) and Azetidine: (C_3_NH_8_)_2_[BiCl_5_], (C_3_NH_8_)_2_[BiBr_5_]

**DOI:** 10.1021/acs.jpclett.4c02695

**Published:** 2024-11-15

**Authors:** Magdalena Rok, Bartosz Zarychta, Jan K. Zaręba, Aleksandra Krupińska, Błażej Dziuk, Piotr Durlak, Rafał Janicki, Ryszard Jakubas, Grażyna Bator, Wojciech Medycki, Michaela Zamponi, Anna Piecha-Bisiorek

**Affiliations:** † Faculty of Chemistry, University of Wroclaw, Joliot-Curie 14, 50-383 Wrocław, Poland; ‡ Faculty of Chemistry, University of Opole, Opole PL-45052, Poland; § Institute of Advanced Materials, Faculty of Chemistry, Wrocław University of Science and Technology, 50-370 Wrocław, Poland; ∥ Institute of Molecular Physics, Polish Academy of Sciences, Smoluchowskiego 17, 60-179 Poznań, Poland; ⊥ Forschungszentrum Jülich GmbH, Jülich Centre for Neutron Science (JCNS) at Heinz Maier-Leibnitz Zentrum (MLZ), Lichtenbergstrasse 1, 85748 Garching, Germany

## Abstract

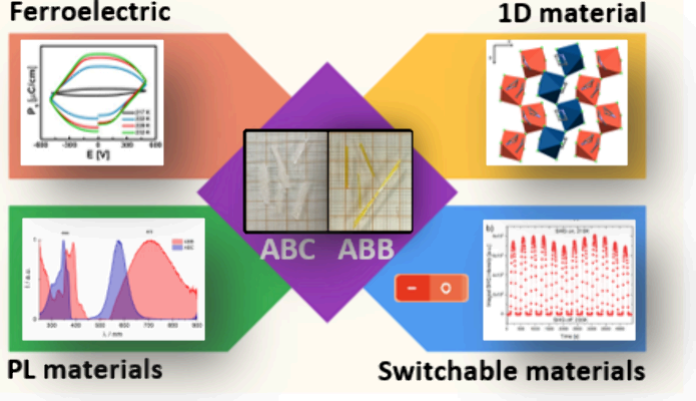

This study investigates lead-free organic–inorganic hybrids (C_3_NH_8_)_2_[BiCl_5_] (**ABC**) and (C_3_NH_8_)_2_[BiBr_5_] (**ABB**), focusing on their structural, dielectric, ferroelectric, and optical properties. Both compounds exhibit paraelectric (**I**) to ferroelectric (**II**) phase transitions (PTs) at 230/233 K and 228/229 K, respectively, transitioning from orthorhombic (*Pnma*) to monoclinic (*P*2_1_) phases, with distorted [BiX_6_]^3–^ octahedra forming 1D chains. Quasielastic neutron scattering and solid-state ^1^H NMR studies reveal the localized motion of azetidinium cations. Dielectric measurements of **ABC** and **ABB** show step-like permittivity changes by 11–12 units post-transition **I** → **II**, demonstrating reversible switching behavior. Absorption studies reveal band gaps of 3.24 eV for **ABC** and 2.76 eV for **ABB**, classifying them as insulators. Luminescence spectra at 77K display 578 nm (**ABC**) and 708 nm (**ABB**) emissions, attributed to ^3^P_1,0_ → ^1^S_0_ transitions. Both compounds demonstrate stable second harmonic generation (SHG) switching of the *on*–*off* type. The switching performance is evaluated over multiple thermal cycles using the *t*_req_ metric, which decreases with increasing temperature change rate and indicates that **ABB**’s SHG switching is approximately 30% faster than that of **ABC**.

Over the past two decades, the chemistry of halobismuthates(III) and haloantimonates(III) has garnered significant interest due to their array of physical properties, such as ferroelectricity, nonlinear optical features, photochromism, and semiconductivity.^1−6^ Particularly, Bi(III) species have emerged as competitive candidates for developing novel lead-free hybrid perovskites with robust chemical stability. This attribute renders them especially valuable for applications in solar cell technologies, where stability and environmental safety are paramount.^7−9^

The general formula for halogenoantimonates(III) and halogenobismuthates(III), R_*a*_M_*b*_X_3*b*+*a*_ where R is an organic cation, M is Sb^3+^ or Bi^3+^, and X is Cl, Br, or I, encapsulates a diverse group of compounds. These compounds have been an active area of research for many years due to their ability to exhibit ferroelectric properties across a relatively large number of systems.^1,10−14^ The fundamental anionic unit, MX_6_, tends to share up to four ligands (X) with adjacent octahedra, forming various anionic units. To date, more than 45 types of these anionic units have been identified, yet ferroelectric properties have been confined to a select few structural arrangements. These include: (i) zero-dimensional (0D) corner-sharing M_2_X_11_ bioctahedron,^15,16^ (ii) (0D) isolated MX_6_ units (only two examples),^17,18^ (iii) 0D face-sharing M_2_X_9_ bioctahedron (extremely rare),^19^ (iv) 2D “honeycomb” M_2_X_9_,^20,21^ (v) 1D [MX_4_]_∞_ chain,^22,23^ and (vi) 1D [MX_5_]_∞_ chain.^24−27^ It should be added that in the case of R_2_MX_5_ stoichiometry 0D isolated square pyramidal [MX_5_]^2–^ units,^28^ [M_2_X_10_]^4–^ bioctahedral,^29,30^ [M_4_X_20_],^8−31^ and 1D anionic network^2,32−35^ are identified (see Scheme 1).

**Scheme 1:**
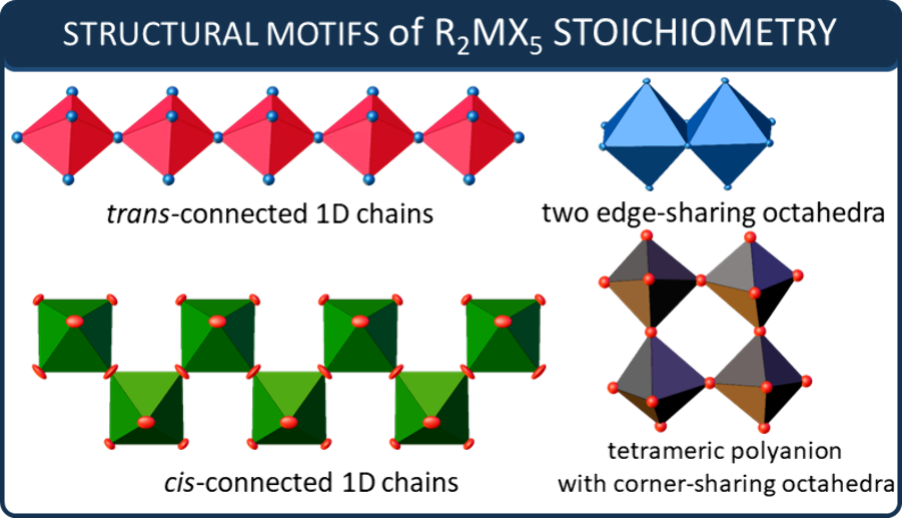
Schematic Projection of Inorganic Structures Occurring in the Halobismuthates(III) and Haloantimonates(III) with the R_2_MX_5_ Chemical Stoichiometry

Among the 1D units, *trans*- and *cis*-connected chains of the MX_6_ octahedra can be distinguished. In the case of R_2_MX_5_ stoichiometry, two halide atoms have a bridging position and four are terminal. Two opposite bridging halide atoms share the vertices of consecutive octahedra, resulting in a linear 1D chain extended in a given direction (*trans*-chain). In contrast, in the *cis*-type chain, two adjacent halides join octahedra to form 1D zigzag chains (as shown in Scheme 1). It is very puzzling that ferroelectric properties are predominantly associated with 1D R_2_MX_5_-type stoichiometry, with the vast majority adopting *cis*-type chains. Ksiądzyna^10^ and Owczarek^36^ made a structural analysis of halogenoantimonates(III) and halogenobismuthates(III) with the stoichiometry R_2_MX_5_ in terms of the possibility of acentric structures depending on the type of the anion sublattice. It has turned out that the acentric arrangement of the crystal structure is preferred for infinite 1D chains in 40% of cases compared to a mere 10% for 0D units. Until now, 14 (1D)-type R_2_MX_5_ ferroelectrics have been synthesized and characterized. Only two compounds, i.e., comprising methylviologen dication adopted *trans*-connected MX_6_ octahedra, and the remainder featured *cis*-type anionic polymer structure.

The ferroelectrics with the *trans*-mode chains exhibit the “displacive” molecular mechanism of ferroelectric PT, attributed to the substantial distortions of the anionic chain. Conversely, the 12 *cis*-mode ferroelectrics displayed a mixed mechanism of PTs, wherein the “order–disorder” contribution seems dominant. Despite the diversity of size and symmetry of the organic cations applied so far in the R_2_MX_5_-type ferroelectrics, no particular preferences have been noted, although extant studies employed both linear aliphatic and cyclic (aromatic and aliphatic) cations. Notably, some members of this family of ferroelectrics, such as (MV)BiI_3_Cl_2_, have demonstrated exceptionally high values of spontaneous polarization (*P*_s_) reaching up to 80 μC·cm^–2^ in the first field sweep and 15 μC·cm^−2^ in the subsequent ones, marking them among the highest values for ferroelectric organic–inorganic hybrids.^24^

Here, we have successfully obtained new lead-free organic–inorganic hybrids based on Bi(III) and azetidinium cations with acronyms **ABC** and **ABB** for (C_3_NH_8_)_2_[BiCl_5_] and (C_3_NH_8_)_2_[BiBr_5_], respectively. The shape and quality of the crystal can be seen in Figure S1 in the Supporting Information (SI). We performed phase-by-phase structural analysis for these crystals. The second harmonic generation (SHG) signal over a wide temperature range and the ferroelectric hysteresis loop were measured to confirm the polar properties of the compounds under investigations. Clear dielectric and nonlinear optical contrast between centric and acentric crystal phases made possible determination of bimodal dielectric and second harmonic generation switching. Two methods, quasi-elastic neutron scattering and solid-state ^1^H NMR measurements, were used to observe molecular dynamics. Theoretical calculations (ab initio density functional theory) supported the experimental results. The powder diffractogram was recorded to confirm the phase compatibility because most physicochemical measurements were performed on the polycrystalline sample (Figure S2, SI).

The structures of **ABC** and **ABB** compounds are isostructural at corresponding temperatures across both high and low-temperature phases, with critical PT temperatures (*T*_c_) observed at 230/233 K and 228/229 K, for **ABC** and **ABB**, respectively. The high-temperature phase is orthorhombic to the *Pnma* space group, while the low-temperature phase is monoclinic (*P*2_1_). Within these phases, the anionic substructures are composed of distorted and disordered [BiX_6_]^3–^ (X= Cl or Br) octahedra that share two *cis* corners with two other neighbors, forming infinite one-dimensional [{BiX_5_}^2–^]_*n*_ chains (Figure 1). The azetidinium cations are located between the inorganic chains with their ammonium groups facing the oppositely charged inorganic polyanions. The crystal data and the structure determination details for (C_3_H_6_NH_2_)_2_[BiX_5_] are listed in Table S1, SI. The bond lengths, angles, shortest contacts between organic and inorganic moieties, and hydrogen bond geometries are presented in Tables S2 and S3, SI.

**Figure 1:**
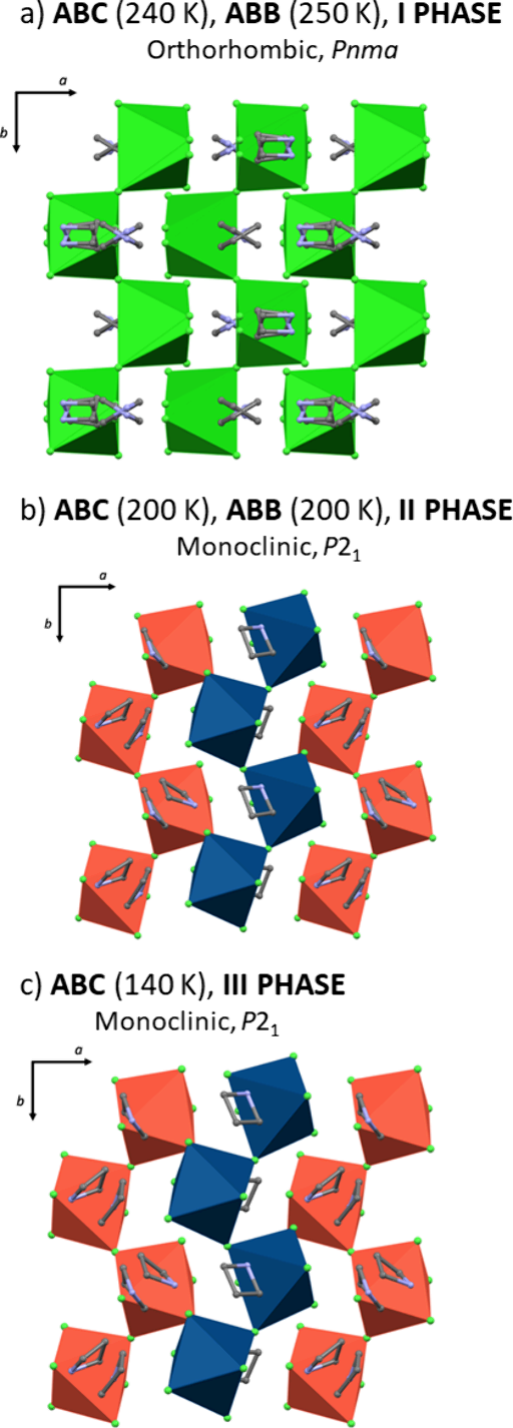
Packing diagrams of the analyzed compounds: (a) **ABC** (240 K) and **ABB** (250 K) in **I** phase, (b) **ABC** (200 K) and **ABB** (200 K) in **II** phase, and (c) ABC (140 K) in **III** phase.

*Structures in Phase **I***. In the high-temperature phase (**I**) of the analyzed compounds, the central bismuth(III) atom is located at the special position (4c Wyckoff position), surrounded by four crystallographically independent bromine ligands to form a [BiX_6_]^3–^ octahedron. The infinite [{BiX_5_}^2–^]_*n*_ chains are extended along the *b* direction of the unit cell. Two axial halogen atoms, i.e., X1 and X2, are disordered, with occupancy factors of 0.5. The longest Bi–X bonds correspond to bridging halogen atoms, and the shortest to terminal, opposite to the bridging ones. The longest Bi–X distances are 2.8706(3) and 3.0452(4) Å for Cl3(Cl3^I^) and Br3(Cl3^I^) (symmetry code: (^I^) −*x*, −1/2+*y*, −*z*), in chlorine and bromine derivatives, respectively, while the shortest Bi–X bond lengths are 2.514(2) and 2.6929(14) Å, respectively. The disordered axial atoms have intermediate lengths. Disparate Bi–X bond lengths are typical of [BiX_5_]^2–^ inorganic substructures. The X–Bi–X angles, involving neighboring *cis* halogen atoms, range from 82.28 (11)° to 96.39 (14)° for the chlorine derivative and from 83.09 (8)° to 96.19 (10)° in the bromine derivative. *Trans* halogen atoms exhibit angles from 165.1 (2)° to 180° for Cl and 168.02 (19)° to 180° for Br. The unit cell incorporates two crystallographically distinct azetidinium cations, which are disordered and occupy special positions. The type of disorder is similar for the two cations. The disorder model suggests that the cation dynamics could be described as vibrational motion (Figure 2). Relatively weak intermolecular interactions of N–H···X and C–H···X types hold the cation, with the shortest N(C)···Cl[Br] distances of 3.31(2) Å [3.49 (3) Å] and 3.29(2) Å [3.48 (3) Å] for N and C, respectively. Besides the interactions within the inorganic substructure, the N···X contacts between oppositely charged moieties also contribute to the deformation of the [BiX_6_]^3–^ polyhedra. The N···X distances are between 3.421(7) and 3.611(9) Å. The observed changes in the Bi–X bond lengths and X–Bi–X angles correlate well with the distances and strengths of the N···X interactions.

**Figure 2:**
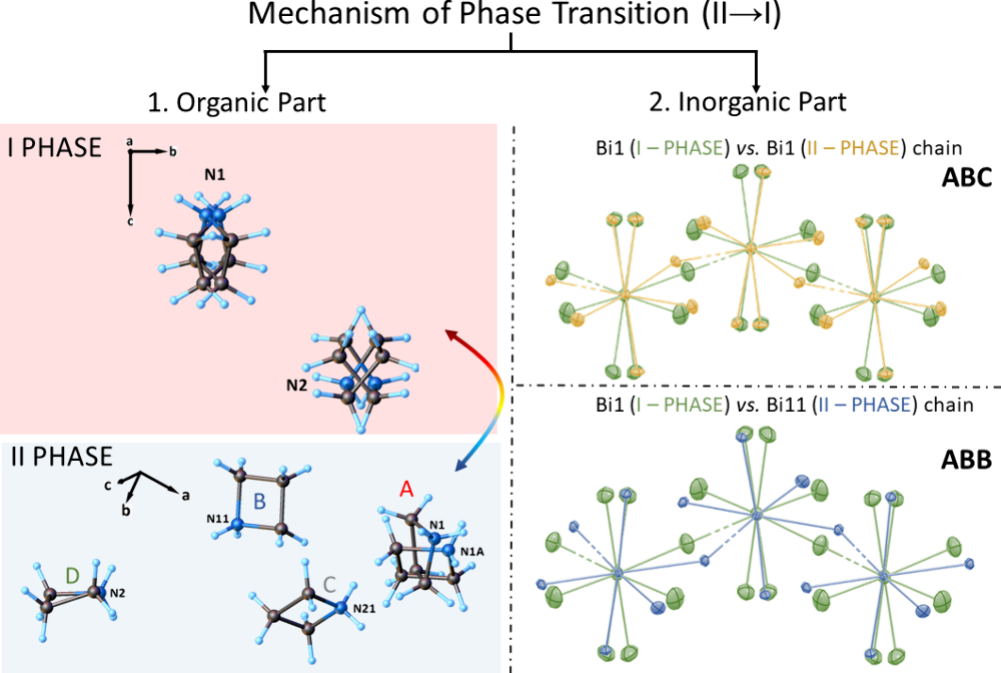
Disorder model of the analyzed compound cations: the left side presents the organic cation changes in phases **I** and **II**, and the right side is the anionic part. The overlay structures of corresponding inorganic sublattices: the difference between structures at 240 and 200 K, Bi1 vs Bi1 chain, and Bi1 vs Bi11 chain for **ABC** and **ABB**, respectively. Anisotropic ellipsoids are drawn at the 10% probability level for clarity.

*Structures of Phase **II***. All of the atoms of (C_3_H_6_NH_2_)_2_[BiX_5_] are in general positions in phase **II**. The number of atoms in the unit cell below PT is doubled, indicating differential changes in the structure. In the asymmetric part of the unit cell, there are two central Bi(III) atoms surrounded by five crystallographically independent halogen atoms, each forming a distorted [BiX_6_]^3–^ octahedra. One of the halogen atoms around Bi1 stays disordered (X1, 0.5 occupation factors), while the ligands around Bi11 are ordered. This structural variance gives rise to two distinct infinite [{BiX_5_}^2–^]*_n_* chains composed of corner-sharing octahedra. The chains are arranged in an alternate way in the structure. There are also four independent azetidinium cations in the asymmetric part of the unit cell. One of them is disordered with an occupation factor of 0.5. The arrangement of the ligands around the bismuth(III) atoms in phase **II** deviates more strongly from the ideal octahedron than in the high-temperature phase (Figure 2, right side). The Bi–X bond length pairs are in the same order as in the structure determined at temperature above phase transition. The lengths of the Bi–Cl[Br] bonds vary from 2.528(12) Å [2.698(4) Å] to 2.937(9) Å [3.059(3) Å]. The difference between the shortest and the longest Bi– Cl[Br] distances are 0.386(26) Å [0.358(7) Å] and 0.409(23) Å [0.379(6) Å] for Bi1 and Bi11, respectively, while for phase I structure it is 0.357(2) Å [0.352(2) Å]. This variation is similarly reflected in the case of the *cis* and *trans* angles (Table S2, SI). The hydrogen bonding net in phase **II** is slightly stronger with the shortest N(C)···Cl[Br] distance of 3.20(5) Å [3.32 (3) Å] and 2.61(11) Å [2.76 (12) Å] for N and C, respectively.

*Phase Transition (**II** → **I**)*. X-ray single crystallographic analysis substantiates that the PT mechanism in the title compounds is connected to changes in the dynamics of both the inorganic and organic substructures. The observed disorder of the ligands around Bi^3+^ atoms, together with relatively large thermal motions, are reflected in the high displacement parameters of the remaining halogen atoms. Additionally, the dynamics of three out of four crystallographically independent azetidinium cations are frozen below the temperature of the phase transition, leading to a change in the crystallographic system and space group (Figure 2 left side).

*Structure of Phase **III** of **ABC***. At first glance, the structure of **ABC** at 140 K (phase III) is essentially the same as that at 200 K. The difference in cell volume equals only 44.83(33) Å^3^, representing only 3% of the high-temperature value. Nevertheless, some minor differences are revealed in the overlaid structures (Figure 3). The angle between octahedra in the Bi1 chain differs by 2.7(5)°. The difference is relatively small; however, the Bi11 chain stays unchanged (Δ_Bi11–Cl31–Bi11_ = 0.8(5)°), which might suggest that the transformation is connected more with the **II** → **III** PT than the regular thermal expansion. Additionally, there is a slight positional shift of the N1 cation at 140 K.

**Figure 3:**
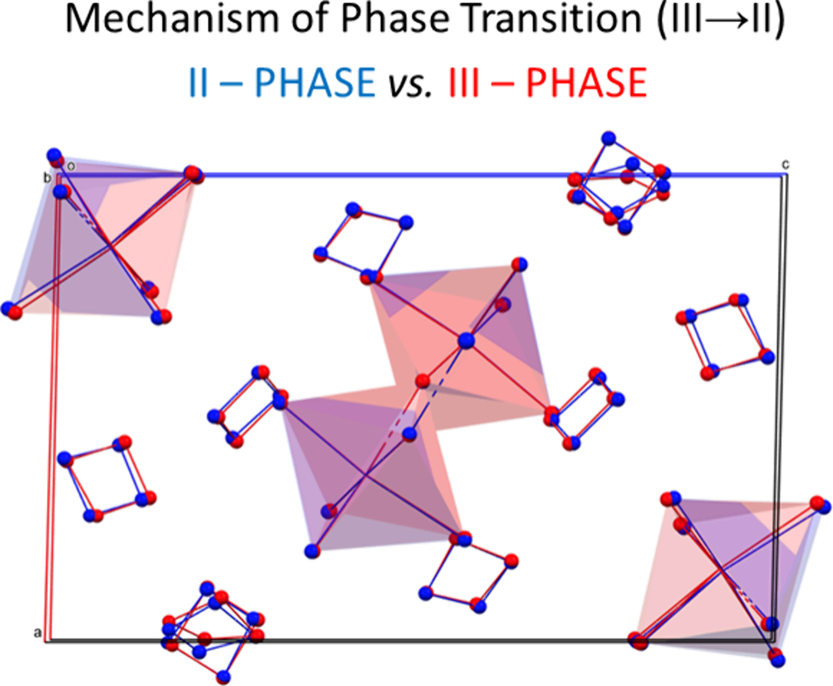
Overlayed organic parts of **ABC** at 200 (blue) and 140 K (red) and inorganic sublattices.

The differential scanning calorimetry (DSC) measurement results are listed in Figure 4. In the DSC diagram for **ABC**, two peaks for the heating–cooling cycles are observed. These anomalies correspond to two structural PT. The temperature values for PTs are the following: for **I** → **II***T* = 230/233 K and for **II** → **III***T* = 179/180 K (on cooling/heating with 0 K/min rate). Thermodynamic parameters of the PTs are collected in Table S4, SI. The estimated values of enthalpy and entropy changes for **I** → **II** PT are equal to Δ*H* = 2950 J/mol and Δ*S* = 13 J/mol·K. The well-shaped anomalies and temperature hysteresis indicate a discontinuous nature of the **I** → **II** PT. The other anomaly (**II** → **III** PT) is significantly more minor with the temperature hysteresis equal to 1 K and a value of Δ*S* = 3.2 J/mol·K. The shape of the thermal anomaly and the entropy transition suggest that this PT is close to a continuous one.

**Figure 4:**
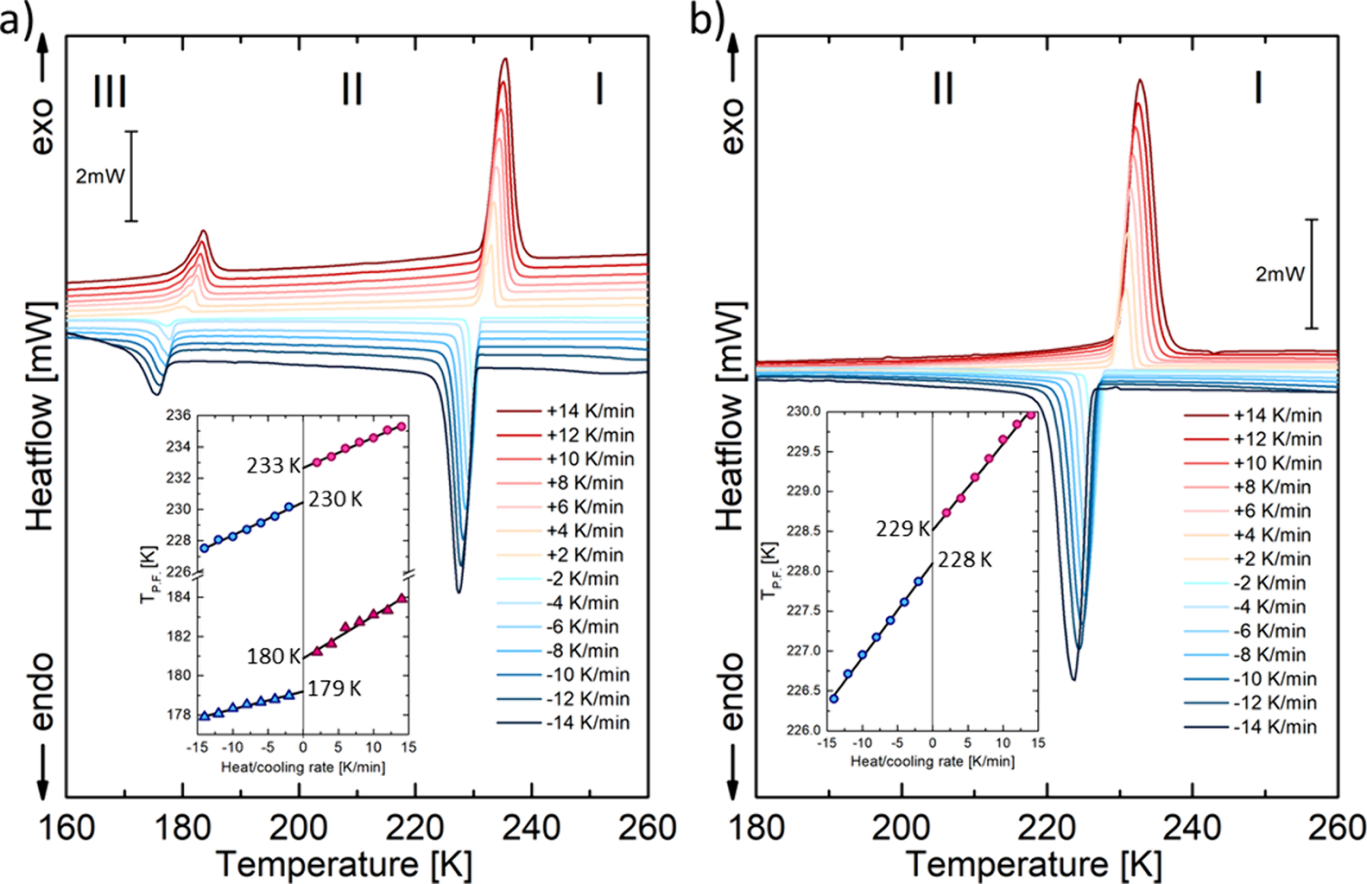
DSC traces for (a) **ABC** and (b) **ABB** crystal were measured on different heating and cooling rates (mass of the sample *m* = 10.006 and 15.885 mg, respectively).

On the other hand, **ABB** undergoes only one reversible PT at 229/228 K on cooling/heating. The **I** → **II** transition exhibit features typical of the discontinuous one, just like **ABC**. Also, the change in enthalpy and entropy transition values of the **I** → **II** PT is similar to that observed for the chloride derivative. Based on the Boltzmann equation (Δ*S* = *R* ln *N*), the ratio of the respective numbers of microstates, *N*, at high temperature (**I**), as well as low temperature (**II**) phases, can be roughly estimated and in the case of **ABC** and **ABB** equals 4.8 and 4.5, respectively. The value of the transition entropy and relatively high value of *N* for the **I** → **II** PT suggest a mechanism of the order–disorder type in both compounds.

To estimate the thermal stability of the **ABC** and **ABB** compounds, we have undertaken thermogravimetric TGA and simultaneous DSC measurements. **ABC** and **ABB** are thermally stable up to about 436 and 450 K, respectively (see Figure S3 in SI). Above those temperatures, we observe a weight loss of more than 3%, which is taken as the limited thermal stability of the material.

Dielectric response measurements were undertaken to determine the dynamics of the dipolar groups of the **ABC** and **ABB** compounds. The complex electric permittivity measurements were performed in the 100–350 K temperature range and 200 Hz to 2 MHz frequency range (see Figure S4, SI). The temperature-dependent profiles of the real part (ε′) of the complex electric permittivity for both **ABC** and **ABB** are depicted in Figure 5. The most spectacular changes in the dielectric response are associated with structural PT (**I** → **II**). The measurements were performed on a polycrystalline sample in the form of a pellet. As the temperature decreases, a notable increase in permittivity occurs at points corresponding to PTs for both **ABC** and **ABB**. In the case of chloride derivative (**ABC**), an additional ε′ change associated with the **II** → **III** PT, is observed as a subtle feature in Figure 5a. Apart from the effects related to PTs, low-frequency dielectric relaxation processes are observed in both crystals (**ABC** and **ABB**).

**Figure 5:**
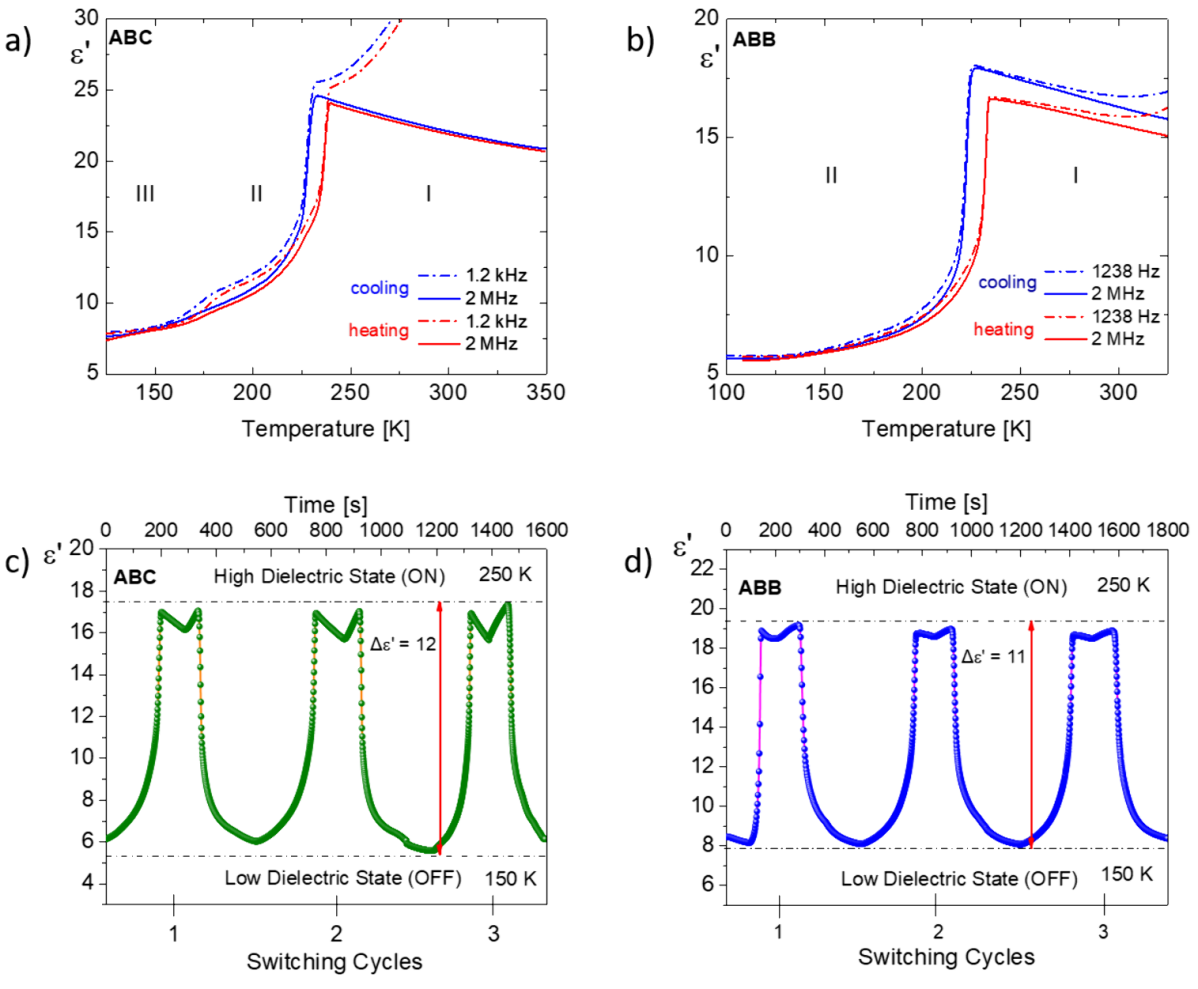
Temperature dependence of the real part, ε′, of the complex permittivity for (a) **ABC** and (b) **ABB** on cooling/heating at two frequencies 1.2 kHz and 2 MHz. Cycles of switching ON and OFF of ε′ between 150 and 250 K measured at 2 MHz for (c) **ABC** and (d) **ABB**.

First, we will focus on the **ABC** crystal as its measured dielectric response is more complex than that for **ABB**. In the low-temperature phase (**III**) only one relaxation process (labeled as 1 in Figure S5, SI) was observed. The experimental data were fitted to the theoretical Cole–Cole model,^37^ as shown in Figure S7a, SI. The fitting results are presented in Figure S6, SI. As the temperature increases, the dielectric relaxation (1) shifts to high frequencies, reaching the microwave range. On the other hand, following the **III** → **II** PT, a new relaxation process emerges, labeled as no. 2 in Figure S5, SI. For temperatures above 200 K, this observed process splits into two distinct relaxation times, 2a and 2b, that differ enough for the simultaneous fitting of two Cole–Cole models (Figure S7a, SI). Just below the **II** → **I** PT temperature, relaxations 2a and 2b disappear. The contribution of the *ac* conductivity is mainly observed within phase **I**. In the case of **ABB**, two relaxation processes are detected in the low-temperature phase (**II**), as illustrated in Figure S7b, SI. It means that two distinct relaxators are observed within phase (**II**) for both **ABC** and **ABB**. Based on the data received from the fitting, the activation energies were estimated using the Arrhenius relation (Figure S8, SI).

Structural transitions with an “order–disorder” mechanism are observed in both **ABC** and **ABB** crystals. The minimal temperature hysteresis in the vicinity of the PT from a phase where a highly dielectric state is observed to a relatively low state classifies these systems into a group of so-called dielectric switches. Multiple switching between low-dielectric and high-dielectric states is a sought-after feature in applications, such as smart electronics, switches, sensors, and transistors. Parts c and d of Figure 5 show an example of reversible dielectric switching between “ON” and “OFF” states at 2 MHz. In phase (**I**), the dielectric constant value for both crystals is about 19 units. After the PT, a jump is observed with increments of about 12 and 11 for **ABC** and **ABB**, respectively. In the case of these two compounds, no weakening of the dielectric signal was observed during cyclic processes, demonstrating the high thermal and electrical stability of the samples.

According to Figure S1, SI, **ABC** and **ABB** crystals crystallize in fragile and brittle needles, necessitating all electrical measurements to be performed on samples compressed into pellets. The sample preparation method can affect the electrical response, especially the shape of the ferroelectric hysteresis loop. Figure S9, SI, illustrates the polarization-electric field intensity (*P*–*E*) relationship measured on the **ABC** sample in its paraelectric phase, where a considerable contribution of dc conductivity is observed.

Figure 6a shows the hysteresis loop measured at 232 K (**II** ferroelectric phase) for **ABC**. This figure reveals the unsaturated *P*–*E* loop for which the further increase in field strength causes the sample’s electrical breakthrough. Nevertheless, the emergence of two opposite peaks due to charge displacement on the IC–E (instantaneous current density-electric field) curve in Figure 6a proves that the **ABC** crystal has two stable states of opposite polarity. For a temperature of 232 K, the spontaneous polarization value is 1.5 μC/cm^2^, while the coercive field is 397 V (2.9 kV/cm) at the limit of the power supply used.

**Figure 6:**
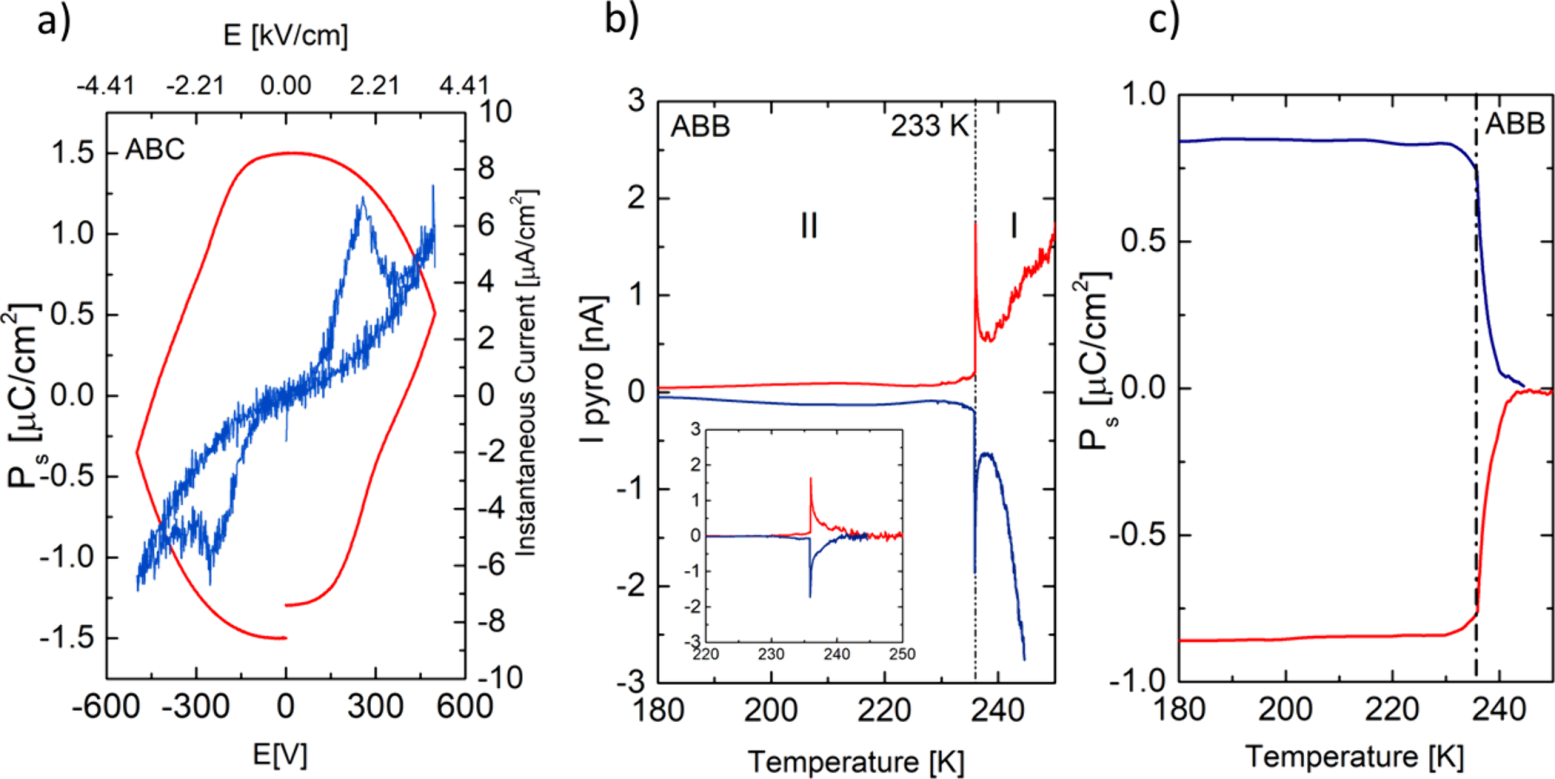
(a) Polarization–electric field (*P*–*E*) hysteresis loops measured for the chosen temperatures (max. electric field of 3.68 kV/cm, *f* = 0.5 Hz, 232 K). The right axis represents the Instantaneous current [μA/cm^2^] curve with two opposite peaks. (b) Temperature dependence of *I*_pyro_ measured for **ABB** sample after applying the dc electric field (±2.35 kV/cm, *d* = 0.85 mm) in phase **I** and then cooling the sample to phase **II**. The measurements of *I*_pyro_ were carried out during heating, (c) polarization *P*_s_(T) determined by the integration of the pyroelectric current.

In the case of **ABB**, obtaining a ferroelectric loop, similar in shape to the **ABC** analogue, was impossible. Problems encountered in this case result from the sample type (polycrystalline pellet). Therefore, the pyroelectric current measurements were attempted for this sample. Due to the strong contribution of dc conductivity, the sample was polarized in high-temperature phase **I** at 250 K and then cooled to 180 K. After 30 min of shorting the sample, the pyroelectric current was measured on the heating run. The results of the pyroelectric current measurements are presented in Figure 6b, whereas Figure 6c shows the temperature dependences of *P*_s_ calculated from the equation: *P*_s_= ∫ *I*_pyro_ d*t*/*S*, where *S* is the contact area of the sample (in this case, the polycrystalline pellet). According to Figure 6b, a strong dc conductivity contribution is observed despite short-circuiting for 30 min of the sample. Therefore, the background was subtracted from the measured current to extract the peak associated with the change in polarization (inset in Figure 6b). The resulting polarization value is 0.8 [μC/cm^2^], which is lower than that obtained from *P*–*E* measurements for **ABC**. Further increases in voltage (max 200 V) destroyed the sample. Nevertheless, reversing the pyrocurrent peak with an external field also suggests the ferroelectric properties of the **ABB** compounds.

Changes in polarization from temperature dependent measurements of pyroelectric current were also determined for the **ABC** sample (Figure S10, SI). Noteworthy is noteworthy that close to the **III** → **II** transition, the observed peak is reversible in the external electric field. This is evidence that in the case of chloride derivative **ABC**, the two phases (**III** and **II**) can be classified as ferroelectric phases.

Figure S11 (SI) presents an example of organic cations that form polar structures with Sb(III) and Bi(III) halides with ferroelectric properties. The data, including PT temperatures, spontaneous polarization values, and anionic sublattice types, are compiled in Table S5, SI. These data suggest that ferroelectric hysteresis loops are primarily recorded when the anionic subnetwork has the shape of a deformed *cis*-chain. In addition, rather significant polarization values are observed for larger (by volume) cations. In the case of **ABC**, not all cation’s orientation contributes to the polarization. Figure 7 shows the packing of the crystal in an elementary cell; the projections of the dipole moments of the A, C, and D cations on the *b*-axis add up along this direction. In contrast, the orientation of the cation labeled B is opposite those mentioned. This spatial orientation, combined with the loop measurements on the polycrystalline sample, may lead to an underestimation of spontaneous polarization.

**Figure 7:**
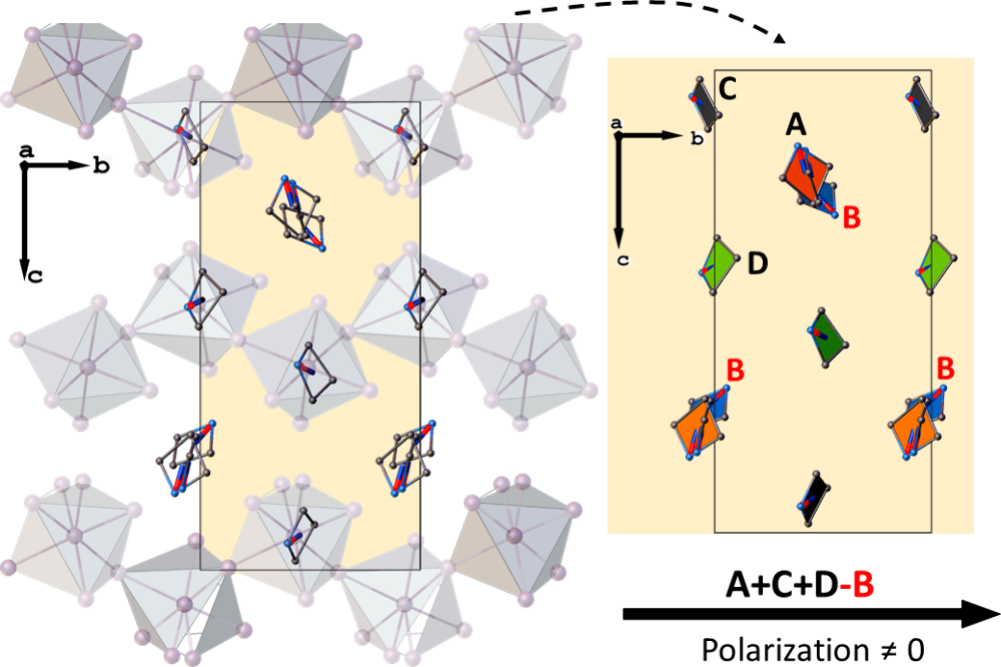
Mutual orientation of the electric dipole moments contributing to *P*_s_ within the cationic network.

The UV–vis reflectance spectra of **ABB** and **ABC** were measured at room temperature (RT). The spectra of the Bi(III) ion may be analyzed in terms of the s → p transitions in the free Bi(III) ion (6s^2^ → 6s6p). The excited 6s6p configuration generates ^3^P and ^1^P levels, which then branch into states in the sequence of increasing energy (^3^P_0_, ^3^P_1_, ^3^P_2_, ^1^P_1_) due to the spin–orbit coupling.^38,39^ A strong absorption band in the spectra of **ABB** starts at about 495 nm (∼2.50 eV), comprising three poorly resolved bands with maxima at about 420, 390, and 340 nm. The absorption of **ABC** is markedly shifted toward the UV region compared to **ABB**; the spectrum extends from about 420 nm (∼2.95 eV) with maxima at about 368, 330, and 290 nm. Compared with the Bi–Br one, the observed hypsochromic shift is brought about by a decrease of the covalent contribution to the Bi–Cl bond.^40^ In order to determine the energy band gap, the Tauc plot^37^ was employed, assuming that the transitions are indirectly allowed. The determined values found to be 2.76 and 3.24 eV for **ABB** and **ABC**, respectively, indicate that the compounds under study may be classified as insulators (Figure 8).

**Figure 8:**
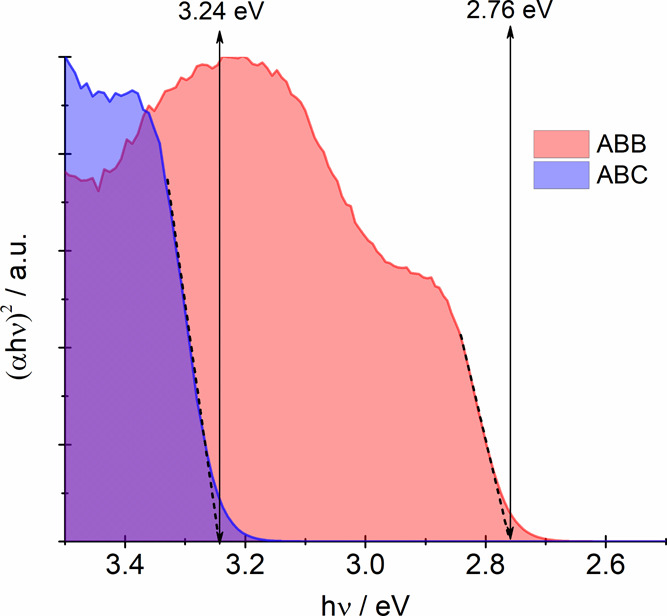
Tauc plot of **ABC** and **ABB**.

Attempts were made to record luminescence spectra at RT, but the compounds under study exhibited luminescence at 77 K. This is a reasonably typical property for compounds consisting of polymeric [{BiX_5_}^2–^]_*n*_ species. The short Bi(III)–Bi(III) distances (6.09 Å for **ABB** and 5.74 Å for **ABC**) result in significant luminescence quenching at room temperature. The luminescence spectra of **ABB** and **ABC** are presented in Figure 9. The emission bands centered at 708 nm (**ABB**) and 578 nm (**ABC**) are attributed to ^3^P_1,0_ → ^1^S_0_ transitions. As can be seen, the excitation emission bands are split. This feature is caused by the second-order Jahn–Teller effect.^39^

**Figure 9:**
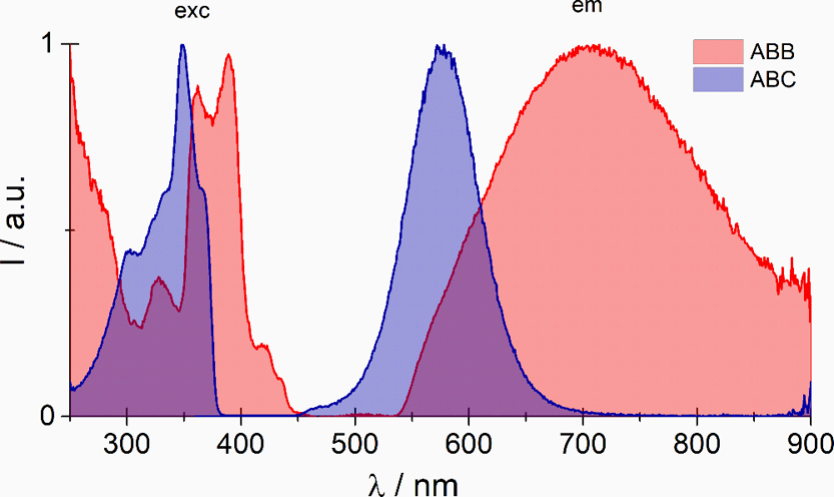
Luminescence spectra of **ABC** and **ABB** at 77 K.

The geometric and lattice parameter optimization of both crystal structures (**ABC** and **ABB**) carried out at the DFT computational method level indicates good agreement of the calculated geometric parameters (bond lengths, angles, dihedral angles, and lattice parameters) with those obtained from measurements carried out using the X-ray method. Please see Figures S12–S13 and Tables S6 and S7 in the SI. As shown in Figure 10, the wide band gaps (about 4.21 eV for **ABC** and 3.78 eV for **ABB**) confirm the insulator characteristics of both materials. The calculated values are about 1 eV higher than those determined experimentally.

**Figure 10:**
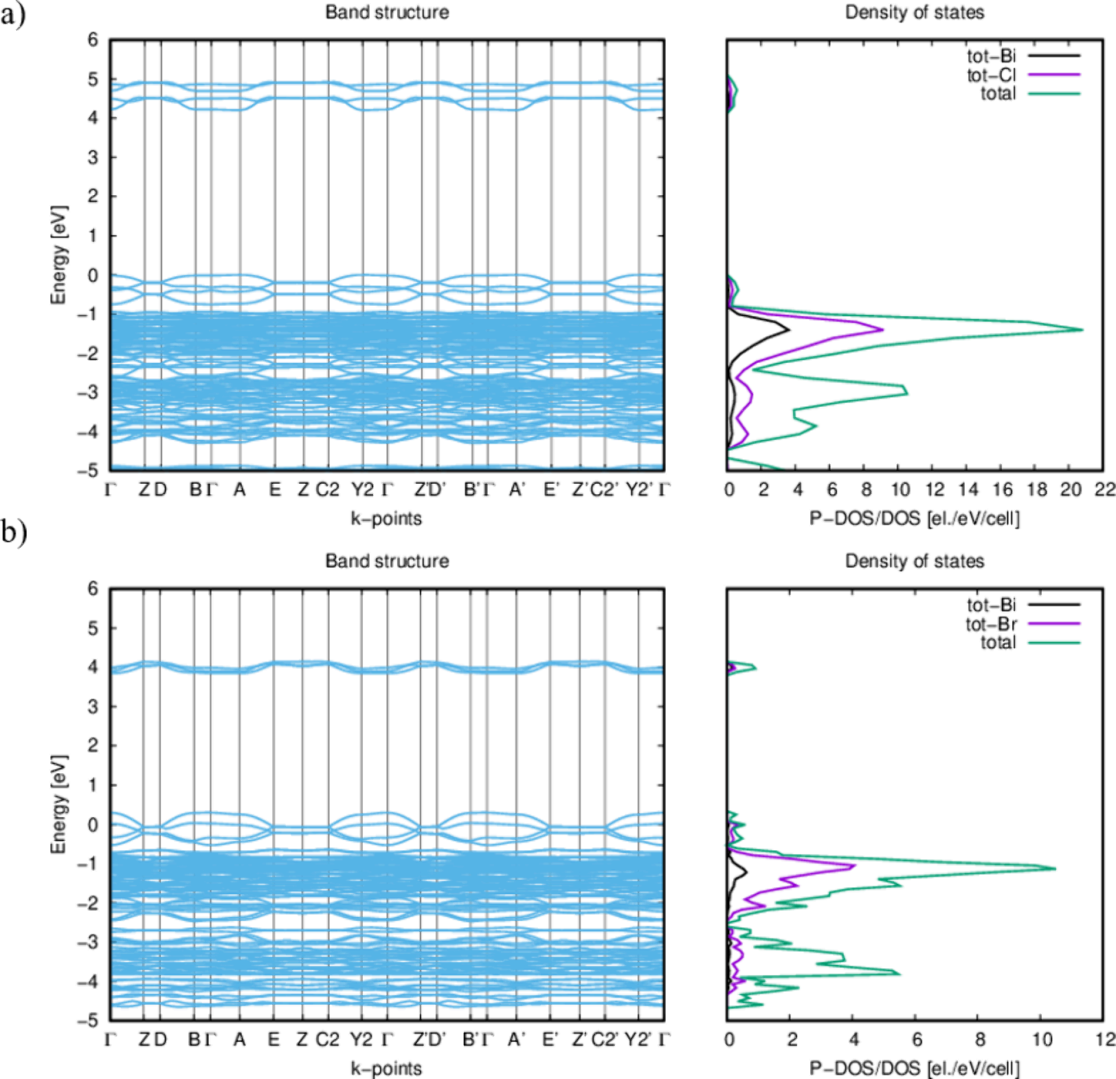
Unified electronic band structure (EBS) of (a) **ABC**: *E*_g_ = 4.21 eV, and (b) **ABB:***E*_g_ = 3.78 eV crystals, and the density of states (DOS|P-DOS) derived from the ab initio calculations.

The densities of states projected onto the energy band diagram show that the highest valence states are predominantly composed of Br(p)/Cl(p) states, whereas the lowest conduction band states primarily involve bismuth p states, as shown in Figure 10 a,b. The conduction band minimum and the valence band maximum are located at the same crystal momentum (*k*-vector) values in the Brillouin zone (Γ point), indicating a direct band gap.

The value calculated for the spontaneous polarization parameter is *P*_s_ = 1.52 μC/cm^2^ and *P*_s_ = 2.05 μC/cm^2^ for structures **ABC** and **ABB**, respectively, and is in excellent agreement with the parameters determined experimentally (1.50 μC/cm^2^ measured for **ABC**).

Subsequently, our investigation extended to temperature-resolved second harmonic generation (TR-SHG) measurements to substantiate the noncentrosymmetric nature of the low-temperature crystal phases of **ABC** and **ABB**. To this end, TR-SHG measurements were performed in the 123–293 K range with 10 K/min rate by irradiation of powdered samples with 1400 nm femtosecond laser pulses. Temperature plots of integral SHG intensities (λ_SHG_ = 700 nm) are provided in Figure 11, while experimental SHG spectral data are provided in Figures S14, S15, SI. The TR-SHG results for **ABC** and **ABB** reveal several shared characteristics. Primarily, for both compounds, one observes an abrupt enhancement in the SHG signal intensity upon cooling, starting at ca. 227 and 221 K, with the signal vanishing at 228 and 223 K upon reheating, respectively. Therefore, it is apparent that the phase transition **I** → **II** in **ABC** and **ABB** is reversible, invokes the loss of the symmetry center, exhibits the first-order (discontinuous) character, and shows narrow thermal hysteresis.

**Figure 11:**
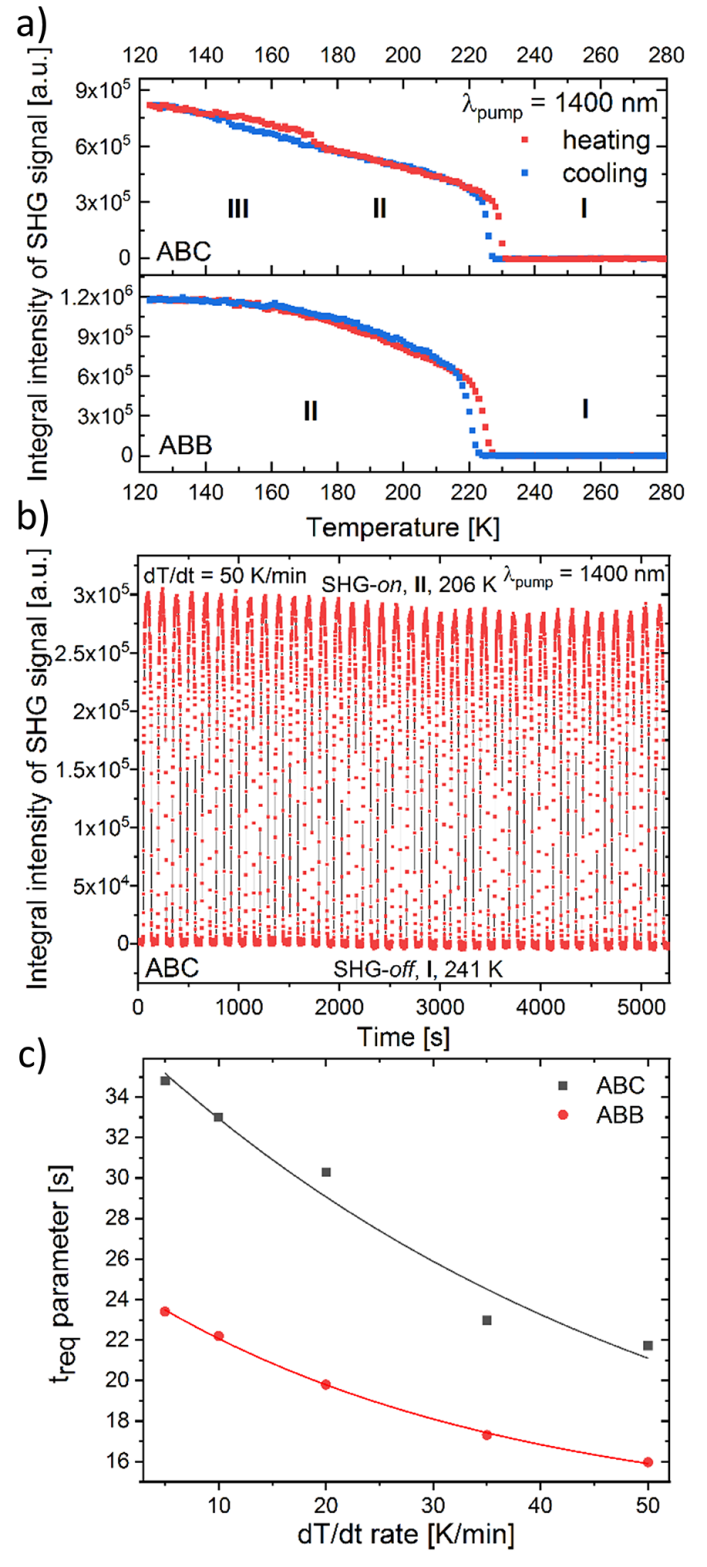
(a) Integral intensities of the SHG signal for **ABC** (upper panel) and **ABB** (lower panel plotted as a function of temperature. (b) The plot of integral intensities of SHG signals (λ_SHG_ = 700 nm) obtained during temperature-induced switching experiment for **ABC** at 50 K/min heating–cooling rate. (c) Plot of determined *t*_req_ parameter vs heating–cooling rate for **ABC** and **ABB.** Lines are provided to guide the eyes.

These features reflect well what was observed from the DSC data for these compounds. Moreover, after transitioning to the noncentrosymmetric phase **II**, with cooling, one observes strong upward drift of SHG intensities in both compounds. This trend aligns with previous TR-SHG results,^41,42^ suggesting the influence of the immobilization of molecular dynamics, particularly of protonated amines, here azetidinium cations, on the SHG response. However, that drift is approximately linear for **ABC**, with a minor plot inflection attributable to the **II** → **III** PT, whereas for **ABB**, the SHG response rises in a nonlinear manner upon cooling, reaching a plateau near 123 K. Considering the dielectric findings previously discussed, the disparities in the relaxation dynamics between **ABC** and **ABB** at low temperatures may offer a preliminary rationale for the observed variances in their low-temperature SHG behaviors.

To estimate how strong the SHG response of **ABC** and **ABB** is, the Kurtz–Perry powder test has been performed at the same wavelength as for TR-SHG studies by comparing the SHG intensities registered at 120 K with that of KDP at 293 K (Figure S16, SI). It turns out that **ABB** exhibits an SHG approximately 1.5 times stronger than KDP, whereas **ABC** exhibits an SHG intensity equivalent to 0.2 times that of KDP.

Note that SHG plots in Figure 11a demonstrate a very good match between heating and cooling runs along with small temperature hysteresis. The capability to completely reverse the phase transition is essential for effective SHG switching, a NLO functionality that has recently garnered significant interest.^43−46^ However, we also note that extant research on SHG switching predominantly examines a narrow range of parameters, including operating temperature, the type and number of distinct SHG states, and occasionally the SHG contrast. Especially in the context of potential applications of switches of this kind, it is surprising that there have been so far little efforts to quantify and characterize the speed at which an SHG switch reacts to temperature changes, making the kinetics of SHG switching largely unexplored. This oversight is primarily because such studies are more often than not conducted at unspecified rate of temperature change (d*T*/d*t*), or at best at a single d*T*/d*t* rate.

The lack of experimental data, along with the absence of quantitative metrics to describe the time needed for an SHG switch to transition between crystal phases, impedes the meaningful comparison of the kinetic performance of different SHG switches at varying rates of temperature change. To address this issue, we have recently introduced the parameter *t*_req_ (time requirement), defined as^47^
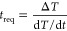
1

This relationship enables the understanding of the time required to complete a structural phase transition, *t*_req_, as the ratio of thermal hysteresis width (Δ*T*) to temperature change rate (d*T*/d*t*). Consequently, this provides a means to determine the duration of the switching event at various heating–cooling rates, thereby facilitating a more comprehensive evaluation of SHG switch performance.

To completely probe the SHG switching properties of **ABC** and **ABB** materials, we conducted experiments examining the reversibility of transitions between their noncentrosymmetric (**II**) and centrosymmetric (**I**) phases across multiple thermal cycles. These experiments were performed under varying temperature change rates (d*T*/d*t*) from slow to rapid (5, 10, 20, 35, and 50 K/min). Figure 11b illustrates a representative example of the SHG switching behavior of the **ABC** sample at a rate of 50 K/min, with temperatures alternating between 241 K (SHG-*off* state) and 206 K (SHG-*on* state) over 39 consecutive heating–cooling cycles. Additional data for other temperature change rates for **ABC** are provided in Figure S17, SI, while a complete data set for **ABB** is available in Figure S18, SI. Generally, each SHG switching plot, regardless of the heating–cooling rate applied, exhibits a stable, nondiminishing SHG signal. There is a minor fluctuation in the SHG-*on* maxima (up to 5% of the total SHG intensity, and in one instance, 10%, as shown in Figure S18e, SI) attributable to the intensity drift of the femtosecond laser pump, rather than the bleaching characteristics of the samples. This level of signal stability is deemed satisfactory, especially considering the quadratic dependence of the SHG signal on the laser intensity. Notably, the same sample of each material was utilized for all switching studies. Thus, by aggregating all the SHG switching cycles experienced by both samples, the switching stability of both **ABC** and **ABB** is validated up to approximately 100 cycles, demonstrating robust and reversible SHG-*on* to SHG-*off* switching behavior. Subsequently, we determined the *t*_req_ parameters for **ABC** and **ABB** (Figure 11c) based on the SHG temperature hysteresis data collected (Figure S19, SI). It is apparent that the *t*_req_ values decrease exponentially with an increasing d*T*/d*t* rate in both compounds, with **ABB** consistently switching approximately 30% faster than **ABC**. Theoretically, a higher temperature change rate should prompt a more rapid phase transition. This is indeed observed; however, the reduction in the *t*_req_ parameter is not substantial. For instance, for **ABC** (**ABB**), a 10-fold increase in the temperature change rate from 5 K/min to 50 K/min reduces the *t*_req_ parameter from 34.8 s (23.4 s) to 21.7 s (16.0 s). This can be attributed to the fact that a higher d*T*/d*t* is kinetically counteracted by a broader temperature hysteresis. This suggests that increasing the temperature change rate is not an effective strategy for achieving a faster SHG or phase switching. Instead, future research should focus on developing materials with reduced *t*_req_ by minimizing temperature hysteresis. Another consideration is how **ABC** and **ABB** compare with other SHG switches in terms of the *t*_req_ parameter. Currently, available data is limited, as *t*_req_ has only been previously calculated for a pyrrolidinium-based cyanide perovskite with the formula pirr_2_KCr(CN)_6_.^47^ This compound exhibited bimodal third-harmonic generation and dielectric switching. At the fastest d*T*/d*t* rate investigated (20 K/min), *t*_req_ was determined to be 1.1 min, which corresponds to approximately 2 (3) times slower switching performance relative to **ABC** (**ABB**).

The interaction between low-energy neutrons and atoms serves as a unique probe for investigating molecular dynamics in the solid state. Nowadays, the quasielastic neutron scattering (QENS) method complements other spectroscopic methods like solid-state NMR, dielectric response, and vibrational spectroscopy, especially in organic–inorganic systems, where organic cations play a crucial role in the physicochemical properties of the bulk material. This is associated with the cross sections (σ) for neutron scattering, providing a metric for the surface area interaction of atomic nuclei with neutrons.

For hydrogen, the incoherent scattering cross-section, σ, equals 80 barns (1 barn = 10^–24^ cm^2^), in the case of the other nucleus from the **ABC** crystal under study, the total scattering cross sections are as follows: C (5.555 b), N (11.5 b), Bi (9.141 b), and Cl (16.7 b). This means that the QENS method is effective for monitoring dynamic changes in the hydrogen-containing organic components of hybrid materials.

Neutron backscattering spectroscopy measures the scattering function *S*(*Q*, ω), where *Q* is the momentum and ω is the energy transferred by the nucleus on which the neutron is scattered. In the case of the crystal we tested, no dependence of the width of the quasi-elastic signal on the value of the momentum transfer was found, indicating a localized motion. In **ABC** crystal, dynamics is primarily related to the rotational movements of the organic molecule, and in our QENS experiment, we used the backscattering spectrometer SPHERES^48,49^ (Spectrometer for High Energy Resolution) to access motions in the time range in the order of ∼100 ps to few ns. Analysis of the QENS data allows us to draw three main conclusions. First, whether changes in the dynamics of the organic part are responsible for the PTs. Second, the relaxation times of the entire molecule and/or specific functional groups are inferred from the modeled half-width at half-maximum Γ_HWHM_. The last conclusion concerns the geometry of motion, which can be proposed based on models of the elastic incoherent structure factor (EISF) in dependence on the momentum transfer between neutron and proton. An elastic fixed window scan was performed to understand the role of organic cation motions in the PT mechanism. Figure 12 presents the dependency of elastic intensity versus temperature measured during the cooling and heating cycle from 3.5 to 280 K. The elastic intensity decreases slowly from 50 to 150 K, followed by a more pronounced drop until 230 K on the heating cycle, where sharp jumps are observed on both cooling and heating cycles. These rapid changes are attributed to the structural **I** → **II** PT, while changes in the vicinity of the **II** → **III** PT do not significantly affect elastic scattering.

**Figure 12:**
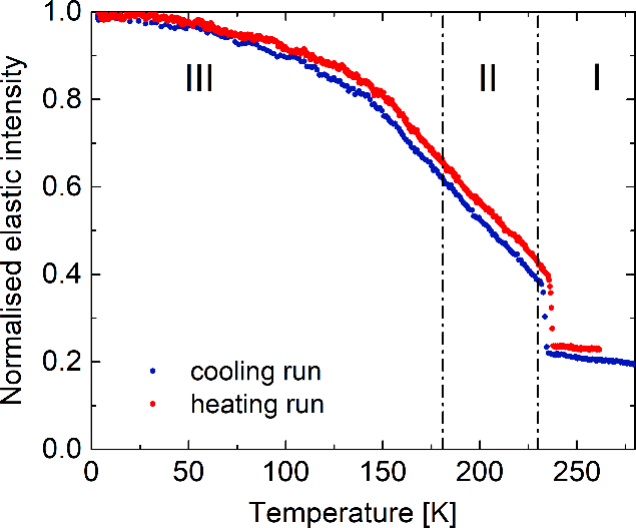
Elastic intensity of neutron scattering vs temperature measured for **ABC**.

Figure 13a presents the examples of the QENS spectra measured in all phases, plotted for detectors averaged over the *Q*-range of 0.6–1.3 Å^–1^. In all phases, the QENS spectra were analyzed using a function consisting of one elastic and one quasielastic component. The elastic neutron scattering is caused by atoms whose movements are too slow to be resolved with the instrument’s resolution and move stochastically (Figure 13b, blue line), while the quasielastic component expresses energy transfer, gain, and loss due to hydrogen motions (green line). The pink line represents the theoretical model convoluted with the instrumental resolution function, which is determined by measuring the purely elastic scattering of the sample at 3.5 K.

**Figure 13:**
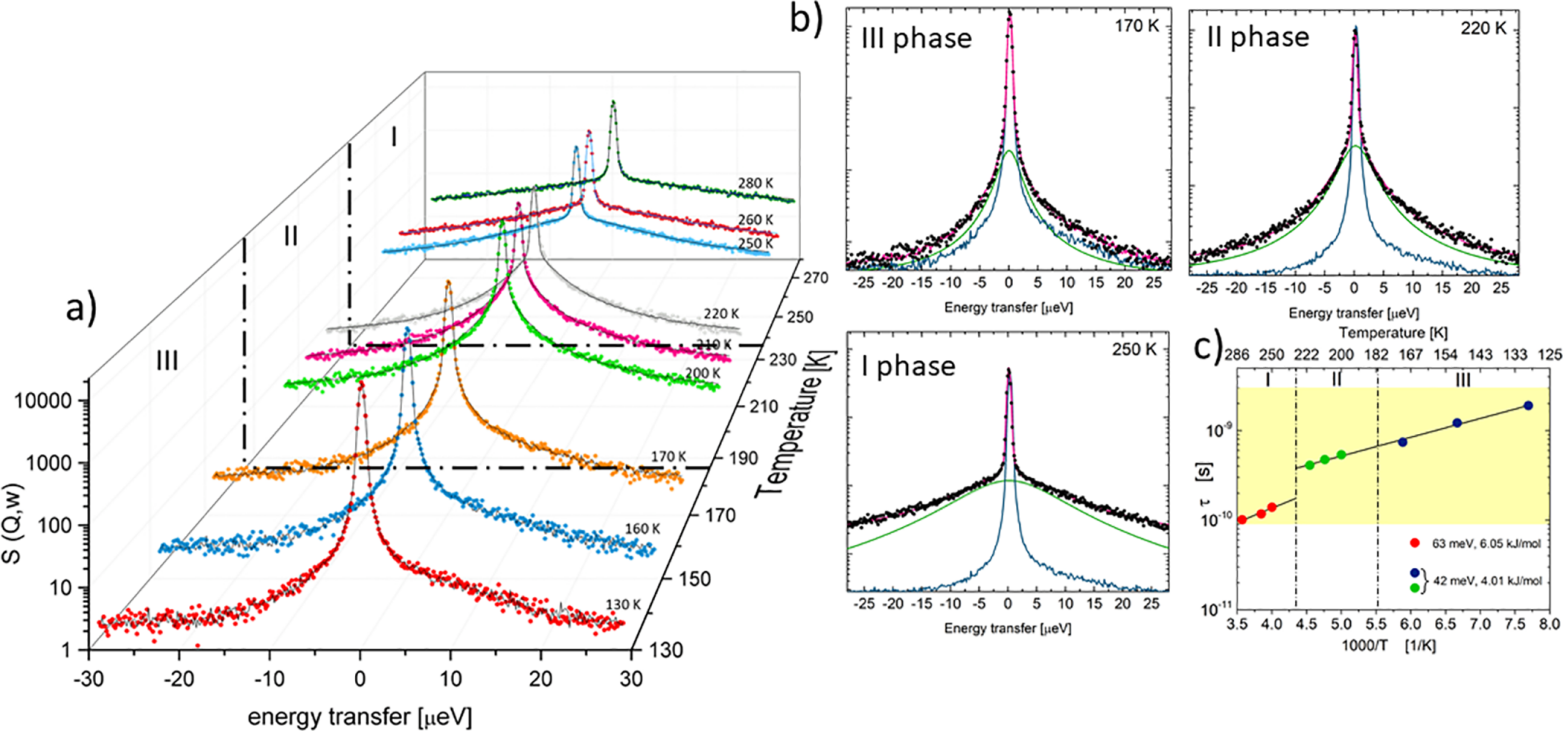
(a) QENS data collected for **ABC** at various temperatures averaged over the *Q*-range of 0.6−1.3 Å^–1^. (b) The QENS broadening was measured in phases **III** (170 K), **II** (220 K), and **I** (250 K). Data collected at 3.5 K was used as the instrument resolution function in the theoretical convolution. Black points represent the experimental data. The solid pink line is the convoluted curve consisting of the instrument resolution with an elastic peak (solid blue line), and one Lorentzian (solid green line). (c) τ of the Lorentzian function used in the fit to the QENS (for average *Q*) data as the Arrhenius plot.

Mathematical notation of the neutron scattering law can be written in the form (eq 2):
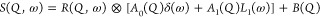
2
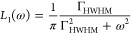
3
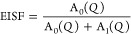
4where *R*(*Q*,ω) is the instrumental resolution, δ(ω) is a Dirac delta function, *L*_1_(ω) is a Lorentzian function defined by eq 3, and Γ_HWHM_ is the bandwidth parameter (half-width at half-maximum, HWHM). The *B*(*Q*) part describes the flat inelastic background in the QENS region, and *A*_0_(*Q*) and *A*_1_(*Q*) are the intensity of purely elastic and quasielastic scattering, respectively. From Γ_HWHM_ the mean time between the proton jumps can be written based on the relationship  [s].^50^

Next, based on the Arrhenius equation, the activation energy for the hindered rotation can be estimated, as shown in Figure 13c. The highlighted yellow area represents the rate of the observed processes that are recorded by the SPHERES spectrometer (between 100 ps and 3 ns). Additionally, the intensities *A*_0_(*Q*) and *A*_1_(*Q*) were used to calculate the EISF (eq 4), in which the *Q* dependence determines the number of sites accessible by the hydrogen atoms and the locations of these sites. The experimentally determined EISF for each phase is presented in Figure 14a. Whereas *Q*-dependence of Γ_HWHM_ estimated from fitting the data taken within **I** phase is illustrated in Figure 14b. Analyzing all of the dependencies obtained on data measured by the SPHERES spectrometer, the following conclusions can be drawn about the dynamics of organic cation movement. First of all, movements of azetidinium cations are relatively slow within phase **II** and especially in phase **III**, which leads to a negligible quasielastic broadening compared with the broadening due to the instrument energy resolution.

**Figure 14:**
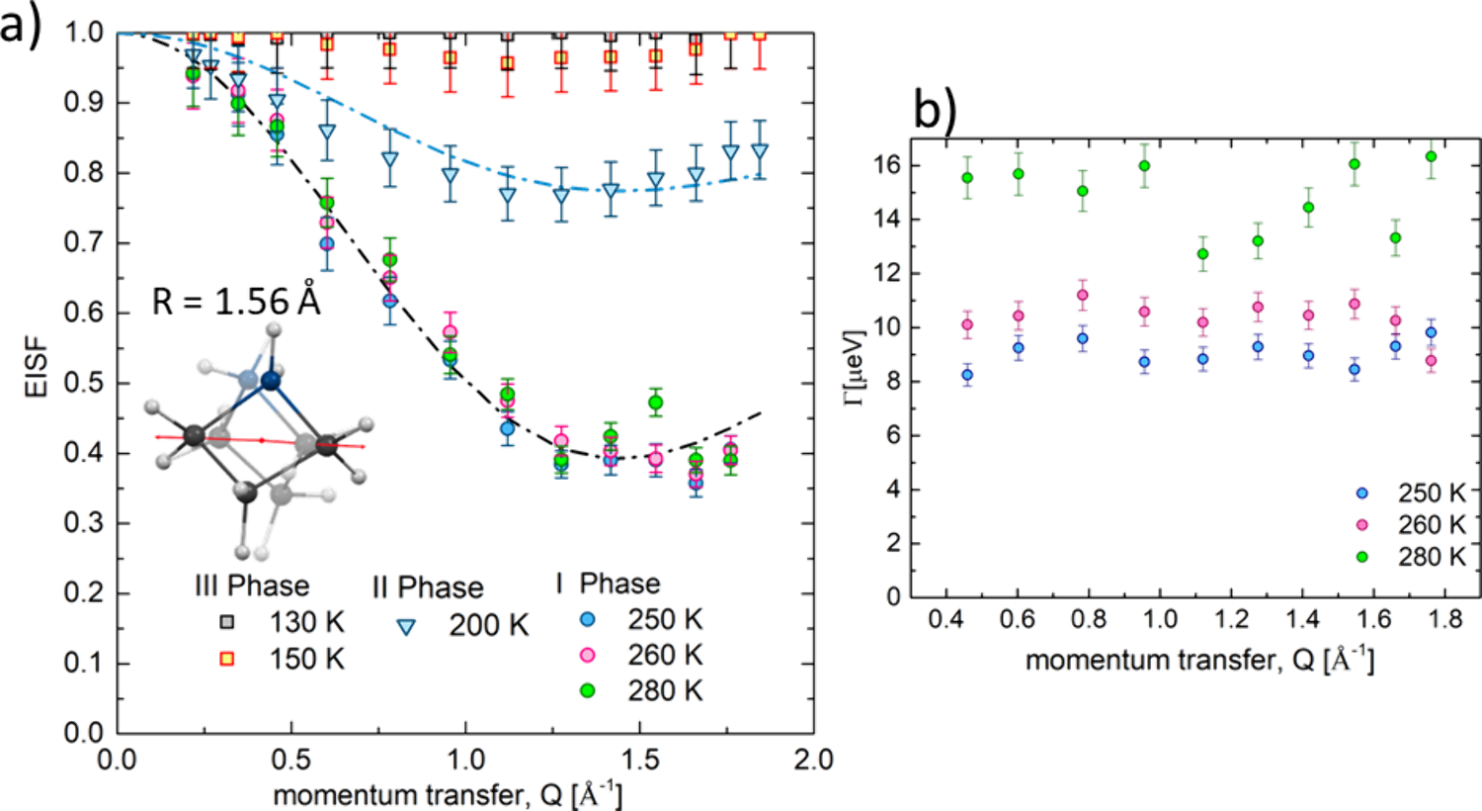
(a) Experimental EISF for all measured within all solid-state phases. (b) *Q*-Dependence of Γ_HWHM_ estimated from fitting to the data taken on the SPHERES spectrometer within **I** phase.

The EISF for temperatures measured in the **III** phase is close to 1, proving that the scattered intensity is almost purely elastic (see eq 4). The dynamics of organic molecules are too slow to be measured by backscattering spectroscopy. In the case of **II** and **I** phases, the proposed dynamics mechanism involves a jump between two equivalent sites and is described by the equation: , where *j*_0_(*x*) = sin(*x*)/*x* is the zeroth-order spherical Bessel function, *R* is radii of rotational reorientation calculated from the geometry of azetidinium molecules based on crystal structure in phase **I**. The value of *p* indicates the percent of molecules involved in the observed dynamics. For the high-temperature phase (**I**), *p* is 100%, meaning that all organic cations perform the same type of movement. In contrast, for phase **II**, *p* is 37%, which is close to data obtained from crystal analysis. Only one model properly adjusts to the EISF experimental data for each temperature from the **II** and **I** solid phases. The path trajectory between equivalent sites is illustrated as the inset in Figure 14a. The radius obtained from the theoretical fit is 1.56 Å, while that determined based on structural data equals 1.52 Å.

Figure 15 illustrates the results of the ^1^H NMR of spin–lattice relaxation times (*T*_1_) measured as a function of the temperature for **ABB**. For this analogue, *T*_1_ changes linearly with temperature between 84 K (*T*_1_ = 1.7s) and 135 K (*T*_1_ = 0.7s), leading to an estimated energy of activation of 1.64 kJ/mol. Such a small value of the longitudinal relaxation *T*_1_ at low temperatures may indicate the dominance of the quadrupole interaction with halogen nuclei. For this compound, a distinct well-shaped minimum of the longitudinal relaxation time below the PT at 233 K is visible, and for its analysis, we used the BPP theory in the form of the formula:^50^
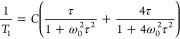
5

**Figure 15:**
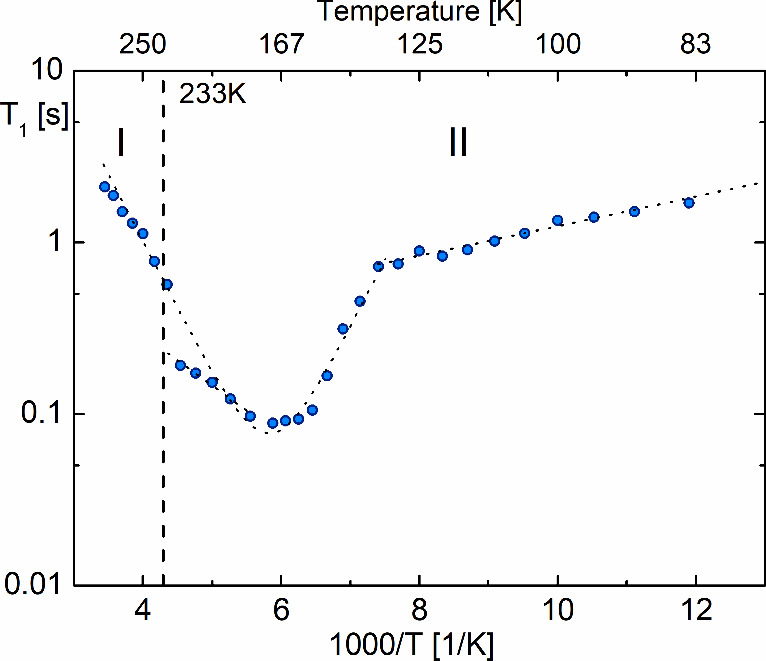
Temperature dependence of ^1^H NMR spin–lattice relaxation time *T*_1_ for **ABB**.

In this equation, ω_0_ represents the Larmor resonance angular frequency, *C* is the relaxation constant, and the τ is the correlation time. The Arrhenius law describes the temperature dependence of correlation time: τ = τ_0_ exp(*E*_a_/*RT*), where τ_0_ is the correlation time at the limit infinite temperature, *E*_a_ is the height of the barrier, and *R* is the gas constant. After the fitting procedure, the activation energy *E*_a_ and the correlation time τ motion were estimated as *E*_a_ = 14.9 kJ/mol, τ = 1.86 × 10^–13^ s, and relaxation constant *C* = 4.05 × 10^9^ s^–2^, respectively. The estimated *E*_a_ over phase **I** is about 12 kJ/mol. A sharp increase in the *T*_1_ value in the vicinity of the **II** → **I** PT confirms a discontinuous PT at 233 K and a distinct change in the motions of the azetidinium cations.

Figure S20 in SI shows the temperature dependence of the second moment values (*M*_2_) for the protons in **ABB**. For this compound, a continuous reduction of *M*_2_ up to ca. 10.3 G^2^ is observed between 110 and 200 K, and then a significant jump of *M*_2_ to approximately 3.7 G^2^ takes place after crossing the PT at 229 K. Starting from 110 K, the libration and ring puckering motion of cations increases, and above 229 K, they definitely switch to axial movement along the N–C axis. The axial movement (two-site model) of the azetidine cation in **ABB**, instead of the puckering–ring movement, leads to the reduction of the *M*_2_ value of the ^1^H NMR line to less than 4 G^2^. This observation aligns with findings by Japanese researchers on azetidinium compounds.^51,52^

In conclusion, two newcomers to the family of organic–inorganic hybrids based on Bi(III) have been synthesized. Both **ABC** and **ABB** adopt uncommon alignment of the layers, which have been created by the *cis* configuration of the  chains. The tested analogues undergo a phase transition from paraelectric **I** (*Pnma*) to ferroelectric phase **II** (*P*2_1_). In the case of **ABC**, with **ABC** experiencing an additional transition to a polar phase **III**. The temperature dependence of the SHG signal confirmed the noncentrosymmetry of phases **II** and **III**. The *P*–*E* hysteresis loop and pyroelectric current confirmed the ferroelectric properties for **ABC** with the spontaneous polarization values of 1.66 μC·cm^–2^. In the case of **ABB**, pyroelectric current measurement also suggests ferroelectric properties of the crystal. Azetidinium cations primarily influence the ferroelectric phase transition, although the distortion of the anionic sublattice should also play a role. Quasielastic neutron scattering and solid-state ^1^H NMR measurements were used to observe the molecular dynamics, revealing the localized motion of azetidinium cations. Theoretical calculations (ab initio density functional theory) supported the experimental results, indicating insulator characteristics and energy band gaps. The energy band gaps of 3.24 eV for **ABC** and 2.76 eV for **ABB** were also determined experimentally from UV–vis and demonstrated a change in the emission color depending on the halogen ligands. Compounds **ABC** and **ABB** display a bimodal dielectric and SHG switching. Dielectric switching employs step-like ε′ permittivity increments of 11–12 units upon **I** → **II** PT, demonstrating reversible dielectric switching behavior. SHG switching in **ABC** and **ABB** has been described using the recently introduced *t*_req_ parameter. This parameter, defined as the ratio of the thermal hysteresis width to temperature change rate, provides a quantitative measure of the time required for SHG switching. Experiments conducted at various temperature change rates (5 to 50 K/min) reveal that *t*_req_ decreases exponentially with increasing d*T*/d*t* rates, indicating faster switching. **ABB** shows a 7.5 times stronger SHG response and switches approximately 30% faster than **ABC**. Despite the faster transitions at higher rates, the reduction in *t*_req_ is limited due to broader temperature hysteresis, highlighting the need for materials with minimized hysteresis for an enhanced SHG switching performance.

## Supporting Information Available

Experimental details (ABC_ABB_ESI) (PDF)
2281321-2281324_global (CIF)
ABD200 K_2301159 (CIF)
Transparent Peer Review report available (PDF)

## Supplementary Material

jz4c02695_si_001.pdf

jz4c02695_si_002.cif

jz4c02695_si_003.cif
