# Supplementary material for: Ferroelectric, Switchable Dielectric and Nonlinear Optical Properties in Inorganic–Organic Lead-Free 1D Hybrids Based on Bi(III) and Azetidine: (C3NH8)2[BiCl5], (C3NH8)2[BiBr5]
Source: J Phys Chem Lett. 2024 Nov 15;15(47):11709–22. doi: 10.1021/acs.jpclett.4c02695 (PMC11613664; doi:10.1021/acs.jpclett.4c02695)
Supplement: Supplementary file 1 — jz4c02695_si_001.pdf [file jz4c02695_si_001.pdf]

Ferroelectric, Switchable Dielectric and Nonlinear Optical Properties in Inorganic-Organic Lead-Free 1D Hybrids Based on Bi(III) and Azetidine: $(C_3NH_8)_2[BiCl_5]$, $(C_3NH_8)_2[BiBr_5]$

Magdalena Rok[a], Bartosz Zarychta[b], Jan K. Zaręba[c], Aleksandra Krupińska[a], Błażej Dziuk[c], Piotr Durlak[a], Rafał Janicki[a], Ryszard Jakubas[a], Grażyna Bator[a], Wojciech Medycki[d], Michaela Zamponi[e], Anna Piecha-Bisiorek[a]*

[a]Faculty of Chemistry, University of Wroclaw, Joliot-Curie 14, 50-383 Wrocław, Poland.
[b]Faculty of Chemistry, University of Opole, Opole PL-45052, Poland
[c]Institute of Advanced Materials, Faculty of Chemistry, Wrocław University of Science and Technology, 50-370 Wrocław, Poland
[d]Institute of Molecular Physics, Polish Academy of Sciences, Smoluchowskiego 17, 60-179 Poznań, Poland.
[e]Forschungszentrum Jülich GmbH, Jülich Centre for Neutron Science (JCNS) at Heinz Maier-Leibnitz Zentrum (MLZ), Lichtenbergstr. 1, 85748 Garching, Germany.
e-mail: magdalena.rok@uwr.edu.pl

TABLE OF CONTENTS

Experimental part 4
- **Synthesis** 4
- **Crystal structure determinations** 6
- **Thermal analysis** 13
- **Electric properties** 14
- **Calculation details** 23
- **UV-vis spectroscopy** 32
- **Second harmonic generation (SHG) studies** 33
- **Neutron Spectroscopy** 40
- **Solid state NMR** 41

References 42

CAPTIONS OF FIGURES

Figure S1. Crystals of (a) **ABC** and (b) **ABB** crystallized from aqueous solution. 4
Figure S2. X-ray diffraction pattern of a) **ABC** and **b) ABB** at 293 K (red line) and calculated (blue line) from crystals structure at 250 and 240K for **ABC** and **ABB**, respectively. Black line represents the difference between experimental and calculated data. 5

Figure S3. The TGA and DSC traces for a) **ABC** and b) **ABB** crystal measured on heating (mass of the sample m =16.535 and 14.846 mg, heating/cooling rate 5 K/min)....... 13
Figure S4. The temperature dependence of the real (a) – **ABC** and c) - **ABB**) and imaginary (b) – **ABC** and d) - **ABB**) parts of the complex electric permittivity at several frequencies from the range 600 Hz - 2 MHz. 15
Figure S5. The temperature and frequency dependence of ε'' measured for **ABC** in frequency ranges from 135 Hz to 2 MHz. White indicates the ac conductivity contribution, while black artificial values of ε''. 16
Figure S6. The frequency dependence of the real (a) and imaginary (b) parts of the complex electric permittivity at several temperatures in phase **III** for **ABC**. 16
Figure S7. Cole–Cole plots for selected temperatures for a) **ABC** and b) **ABB** in frequency ranges from 135 Hz to 2 MHz. 17
Figure S8. Arrhenius plot of relaxation time measured for a) **ABC** in the region of two phases (**III** and **II**), and b) **ABB** measured only within one phase **II**. 18
Figure S9. Polarization–electric field (P–E) hysteresis loops measured for RT (black line, max. electric field of 5.21 kV/cm, f = 0.5 Hz). b) IC–E (instantaneous current (mA) density–electric field) red line.. 19
Figure S10. a) Temperature dependence of I_{pyro} measured for **ABC** sample after applying the DC electric field (±3.33 kV/cm, d = 1.2 mm) in phase **I** and then cooling the sample to phase **II**. The measurements of I_{pyro} were carried out during heating, b) Polarization P_s(T) determined by the integration of the pyroelectric current. 20
Figure S11. Molecular structure of the organic cations creating ferroelectric compounds among halobismuthates(III) and haloantimonates(III) with R_2MX_3 stoichiometry. 21
Figure S12. Structure and geometric parameters of the **ABC** crystal after optimization at the DFT (HSE06-D3/pob_TZVP_rev2) method level. 24
Figure S13. Structure and geometric parameters of the **ABB** crystal after optimization at the DFT (HSE06-D3/pob_TZVP_rev2) method level. 24
Figure S14. Overlay of experimental SHG spectra obtained upon irradiation with 1400 nm femtosecond laser pulses of **ABC** for (a) cooling in 293 K - 123 K range, (b) heating in 123 K - 293 K range. 34
Figure S15. Overlay of experimental SHG spectra obtained upon irradiation with 1400 nm femtosecond laser pulses of **ABB** for (a) cooling in 293 K - 123 K range, (b) heating in 123 K - 293 K range. 35
Figure S16. Overlay of SHG traces of **ABC** and **ABB** with that of KDP obtained upon irradiation with 1400 nm femtosecond laser pulses. SHG traces are normalized to the same integration time. 36
Figure S17. Plots of integral intensities of SHG signals (λ_{SHG} = 700 nm) obtained during temperature-induced switching experiment for **ABC** at a) 5 K/min, b) 10 K/min, c) 20 K/min, d) 35 K/min heating-cooling rate. 37
Figure S18. Plots of integral intensities of SHG signals (λ_{SHG} = 700 nm) obtained during temperature-induced switching experiment for **ABB** at a) 5 K/min, b) 10 K/min, c) 20 K/min, d) 35 K/min, e) 50 K/min heating-cooling rate. 38
Figure S19. SHG temperature hystereses registered for different temperature change rates (5, 10, 20, 35, and 50 K/min) for a) **ABC** and b) **ABB**. 39
Figure S20. Temperature dependence of the second moment of ^{1}HNMR line of **ABB** 41

CAPTIONS OF TABLES

Table S1. XRD experimental details. 7
Table S2. Geometric parameters (Å,°). 8
Table S3. Selected hydrogen-bond parameters. 12
Table S4. Thermodynamics parameters, ΔH and ΔS, and parameter N calculated based on Boltzmann relationship. 13
Table S5. Compilation of the Most Important Parameters Characterizing Structural and Spontaneous Polarization Properties for Ferroelectric among halobismuthates(III) and haloantimonates(III) with R_2MX_3 stoichiometry. 22
Table S6. Geometric and lattice parameters of the **ABC** crystal after optimization at the DFT (HSE06-D3/pob_TZVP_rev2) method level. 25
Table S7. Geometric and lattice parameters of the **ABB** crystal after optimization at the DFT (HSE06-D3/pob_TZVP_rev2) method level. 28

Experimental part

Synthesis

The materials necessary for the synthesis of $(C_3H_8N)_2[BiCl_5]$ (**ABC**), $(C_3H_8N)_2[BiBr_5]$ (**ABB**) were purchased from commercial sources (Bi_2O_3, 99.999%, azetidine 98%, Sigma-Aldrich) and used without further purification. 2g of Bi_2O_3 was suspended in 50 ml of distilled water, and then HX acid (X: Cl, Br) was added to dissolve the precipitate. 1g of amine was weighed into a separate vessel with 50ml of water, and a few drops of concentrated HX acid were added to this solution. After cooling, the contents were carefully combined with the bismuth salt solution, and after a week, the compound crystallized. An elemental analysis verified the crystals composition: **ABC** C: 14.20% (theor. 14.34%), N: 5.47% (theor 5.58%), H: 3.02% (theor. 3.21%) and **ABB** C: 10.22% (theor. 9.94%), N: 3.89% (theor 3.87%), H: 2.1% (theor. 2.22%). The single crystals suitable for X-ray measurements were grown from an aqueous solution at a constant room temperature. Powder X-ray diffraction verified the phase purity (see Fig. S1). Powder X-ray diffraction was recorded using an X'Pert PRO powder diffractometer operating with Cu Ka radiation

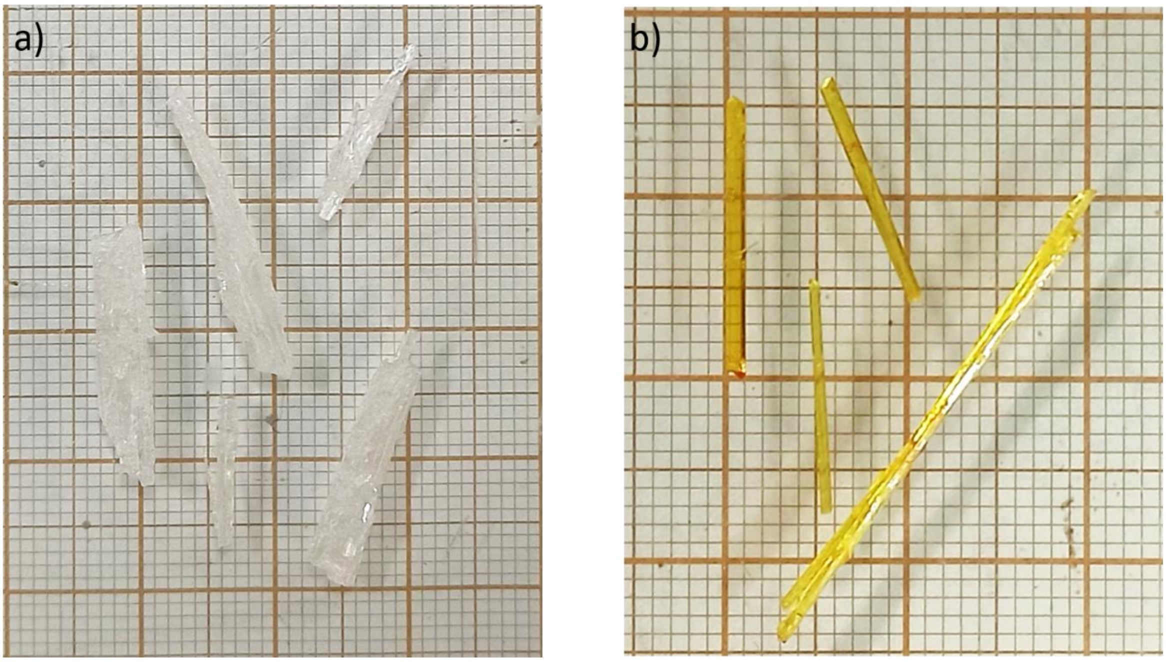

Figure S1. Crystals of (a) **ABC** and (b) **ABB** crystallized from aqueous solution.

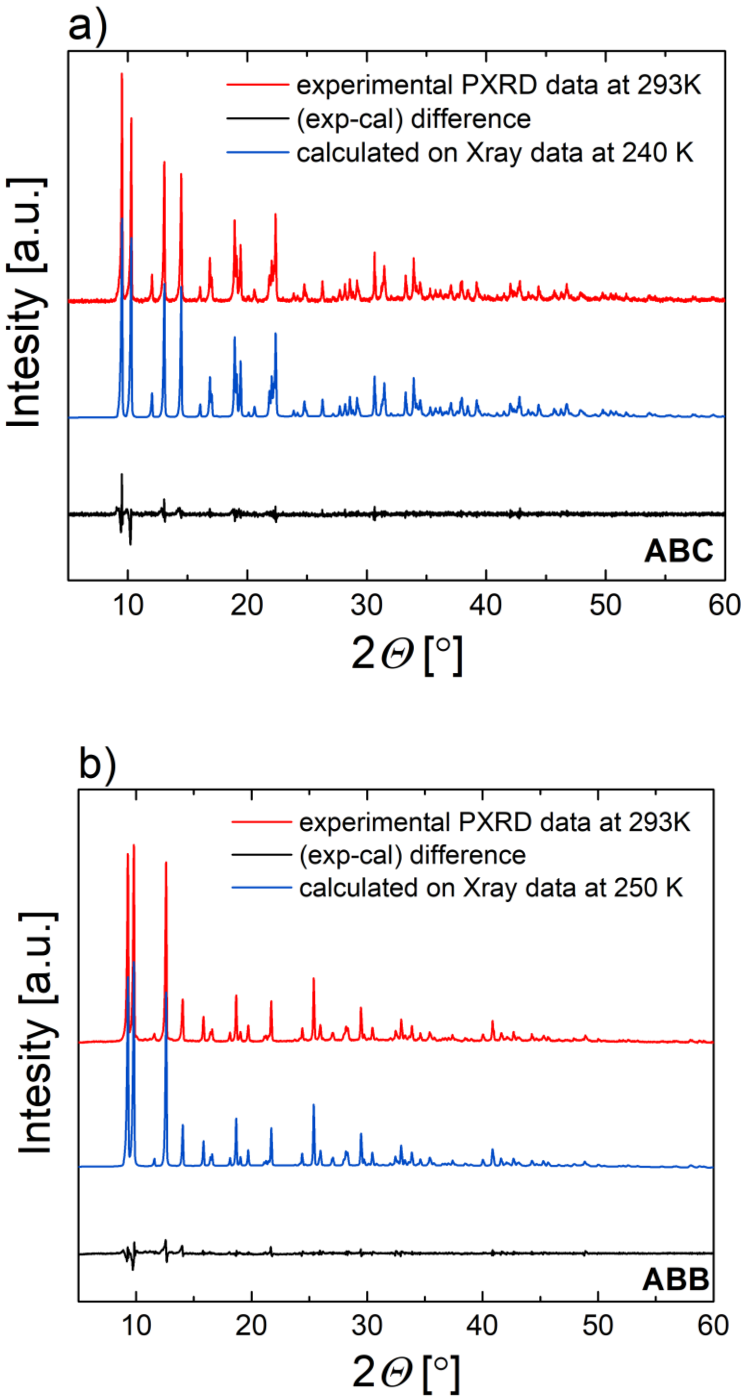

Figure S2. X-ray diffraction pattern of a) **ABC** and **b) ABB** at 293 K (red line) and calculated (blue line) from crystals structure at 250 and 240K for **ABC** and **ABB**, respectively. Black line represents the difference between experimental and calculated data.

Crystal structure determinations

The measurements of **ABC** at 240, 200 and 140 K were performed on a Xcalibur CCD diffractometer with graphite monochromated Mo-Kα (λ=0.71073Å) radiation. The measurements of **ABB** at 250 and 200 K were performed on a Rigaku XtaLAB Synergy R, DW system, HyPix-Arc 150 (graphite monochromatic, MoKα radiation, λ = 0.71073 Å). The reflections were measured using the ω-scan technique. All structures were solved by the Patterson method and the data were subjected to Lorentz, polarisation and empirical absorption corrections based on symmetry-equivalent reflections.[1] The low temperature phases (i.e. **ABC** at 200 and 140 K and **ABB** at 200 K) the structures are twinned and were refined against HKLF5 file format. Fourier maps revealed a dynamical disorder of organic cations for all structures and we were thus all hydrogen atoms were located in subsequent maps and then constrained to a distance of 0.96 and 0.89 Å for >CH2 – and >NH2 groups respectively. The hydrogen atoms were allowed to ride on the C and N atoms. Their displacement parameters were taken with coefficients 1.2 times larger than the respective parameters of the nitrogen, methylene and methyl carbon atoms. The Oxford Diffraction software CrysAlisCCD and CrysAlisRED programs were used during the data collection, cell refinement and data reduction processes.[1] The SHELX program[2] was used for the structure solution and refinement. The structure drawings were prepared using Mercury 2022.3.[3] Crystallographic data (excluding structure factors) for bis(azetidinium) pentachlorobizmuthate(III) at 240, 200 and 140 K and for bis(azetidinium) pentabromobizmuthate(III) at 250 and 200 K have been deposited at the Cambridge Crystallographic Data Centre as supplementary publication nos. CCDC 2281321, CCDC 2301159 and CCDC 2281322 for **ABC** at 140, 200 and 240 K and CCDC 2281323-2281324 for **ABB** at 200 and 250 K respectively. Copies of the data can be obtained, free of charge, on application to the Director, CCDC, 12 Union Road, Cambridge CB2 1EZ, UK (Fax: int. code+(1223) 336033 or e-mail: data request@ccdc.cam.ac.uk).

Table S1. XRD experimental details.

Crystal data					
Chemical formula	$2(BiCl_5)\cdot4(C_3H_8N)$			$2(BiBr_5)\cdot4(C_3H_8N)$	
M_r	1004.87			1449.47	
Crystal system, space group	Monoclinic, *P*2$_1$		Orthorhombic, *Pnma*	Monoclinic, *P*2$_1$	Orthorhombic, *Pnma*
Temperature (K)	140	200	240	200	250
a, *b*, *c* (Å)	10.8163(9), 7.9049(5), 16.7603 (12)	10.9363 (6), 7.9672 (5), 16.9633 (10)	10.7923(8), 8.0368(5), 16.8309 (13)	11.3007(3), 8.2430(2), 17.5993 (5)	11.0937(6), 8.3565(4), 17.9062 (11)
α, β, γ (°)	90, 91.253 (8), 90	90, 91.518 (6), 90	90, 90, 90	90, 91.162 (3), 90	90, 90, 90
V (Å^3)	1432.70 (18)	1477.53 (15)	1459.84 (18)	1639.08 (8)	1659.99 (16)
Z	2	2	4	2	4
Radiation type	Mo *K*α				
μ (mm^{-1})	13.21	12.81	12.96	22.92	22.63
Crystal size (mm)	0.3 × 0.25 × 0.13	0.3 × 0.25 × 0.13	0.3 × 0.25 × 0.13	0.14 × 0.04 × 0.03	0.14 × 0.04 × 0.03
**Data collection**					
Diffractometer	Oxford Diffraction Xcalibur System			XtaLAB Synergy R, DW system, HyPix-Arc 150	
Absorption correction	Multi-scan<br>*CrysAlis PRO* 1.171.41.93a (Rigaku Oxford Diffraction, 2020) Empirical absorption correction using spherical harmonics, implemented in SCALE3 ABSPACK scaling algorithm.				
T_{min}, T_{max}	0.455, 1.000	0.837, 1.000	0.314, 1.000	0.220, 1.000	0.031, 1.000
No. of measured, independent and observed [$I > 2\sigma(I)$] reflections	4975, 4975, 4525	11619, 11619, 10794	9402, 1538, 1132	8962, 8962, 8174	11632, 1749, 1375
$(\sin \theta/\lambda)_{max}$(Å^{-1})	0.617	0.617	0.616	0.617	0.617
**Refinement**					
$R[F^2 > 2\sigma(F^2)]$, $wR(F^2)$, *S*	0.059, 0.143, 1.05	0.076, 0.199, 1.10	0.035, 0.073, 1.05	0.046, 0.124, 1.08	0.045, 0.128, 1.07
No. of reflections	4975	11619	1538	8962	1749
No. of parameters	299	299	112	299	112

No. of restraints	229	235	84	235	72
H-atom treatment	H-atom parameters constrained				
	$w = 1/[\sigma^2(F_o^2) + (0.0794P)^2 + 20.4154P]$ where $P = (F_o^2 + 2F_c^2)/3$	w = 1/[σ2(*F*o2) + (0.151*P*)2] where *P* = (*F*o2 + 2*F*c2)/3	$w = 1/[\sigma^2(F_o^2) + (0.031P)^2]$ where $P = (F_o^2 + 2F_c^2)/3$	$w = 1/[\sigma^2(F_o^2) + (0.0646P)^2 + 18.3529P]$ where $P = (F_o^2 + 2F_c^2)/3$	$w = 1/[\sigma^2(F_o^2) + (0.0695P)^2 + 4.2221P]$ where $P = (F_o^2 + 2F_c^2)/3$
$\Delta\rho_{max}$, $\Delta\rho_{min}$(e Å^{-3})	3.25, −3.23	4.02, −3.16	0.92, −0.79	2.13, −0.87	0.85, −1.60
Absolute structure parameter	0.476 (18)	0.47 (2)	–	0.308 (11)	–

Table S2. Geometric parameters (Å,°).

**ABC**: $(C_3H_6NH_2)_2[BiCl_5]$ 140K (**III PHASE**)			
Bi1—Cl4A	2.531 (12)	C2—C3	1.44 (14)
Bi1—Cl4	2.546 (11)	N1A—C3A	1.44 (10)
Bi1—Cl1A	2.58 (2)	N1A—C1A	1.60 (10)
Bi1—Cl1B	2.58 (2)	C1A—C2A	1.42 (12)
Bi1—Cl2	2.756 (10)	C2A—C3A	1.60 (10)
Bi1—Cl3	2.883 (10)	N2—C4	1.50 (5)
Bi1—Cl3^{i}	2.903 (12)	N2—C6	1.50 (5)
Cl3—Bi1^{ii}	2.903 (12)	C4—C5	1.51 (6)
Bi11—Cl42	2.508 (10)	C5—C6	1.48 (6)
Bi11—Cl41	2.554 (10)	N11—C11	1.47 (5)
Bi11—Cl11	2.616 (9)	N11—C31	1.50 (5)
Bi11—Cl21	2.760 (9)	C11—C21	1.47 (5)
Bi11—Cl31	2.873 (10)	C21—C31	1.46 (6)
Bi11—Cl31^{iii}	2.926 (9)	N21—C41	1.41 (6)
Cl31—Bi11^{iv}	2.926 (9)	N21—C61	1.50 (5)
N1—C3	1.46 (13)	C41—C51	1.45 (5)
N1—C1	1.53 (11)	C51—C61	1.54 (7)
C1—C2	1.66 (15)		
Cl4A—Bi1—Cl4	94.6 (4)	Cl3—Bi1—Cl3^{i}	88.06 (8)
Cl4A—Bi1—Cl1A	101.0 (9)	Bi1—Cl3—Bi1^{ii}	159.2 (5)
Cl4—Bi1—Cl1A	87.8 (6)	Cl42—Bi11—Cl41	92.9 (4)
Cl4A—Bi1—Cl1B	82.1 (8)	Cl42—Bi11—Cl11	92.1 (4)
Cl4—Bi1—Cl1B	97.6 (8)	Cl41—Bi11—Cl11	92.5 (4)
Cl4A—Bi1—Cl2	88.4 (6)	Cl42—Bi11—Cl21	96.0 (3)
Cl4—Bi1—Cl2	95.9 (5)	Cl41—Bi11—Cl21	87.7 (3)
Cl1A—Bi1—Cl2	169.7 (9)	Cl11—Bi11—Cl21	171.9 (3)
Cl1B—Bi1—Cl2	164.1 (10)	Cl42—Bi11—Cl31	87.8 (4)
Cl4A—Bi1—Cl3	88.7 (4)	Cl41—Bi11—Cl31	178.7 (3)
Cl4—Bi1—Cl3	175.3 (4)	Cl11—Bi11—Cl31	88.6 (4)
Cl1A—Bi1—Cl3	88.2 (6)	Cl21—Bi11—Cl31	91.1 (3)
Cl1B—Bi1—Cl3	79.5 (8)	Cl42—Bi11—Cl31^{iii}	176.7 (3)
Cl2—Bi1—Cl3	87.5 (4)	Cl41—Bi11—Cl31^{iii}	87.8 (3)
Cl4A—Bi1—Cl3^{i}	176.8 (4)	Cl11—Bi11—Cl31^{iii}	84.7 (3)

Cl4—Bi1—Cl3^{i}	88.7 (4)	Cl21—Bi11—Cl31^{iii}	87.2 (3)
Cl1A—Bi1—Cl3^{i}	79.3 (9)	Cl31—Bi11—Cl31^{iii}	91.64 (11)
Cl1B—Bi1—Cl3^{i}	97.7 (7)	Bi11—Cl31—Bi11^{iv}	146.0 (4)
Cl2—Bi1—Cl3^{i}	91.1 (5)		

**ABC**: $(C_3H_6NH_2)_2[BiCl_5]$ 200K (**II PHASE**)			
Bi1—Cl4A	2.547 (13)	C2—C3	1.52 (19)
Bi1—Cl4	2.551 (13)	N1A—C3A	1.42 (12)
Bi1—Cl1A	2.59 (4)	N1A—C1A	1.57 (15)
Bi1—Cl1B	2.61 (3)	C1A—C2A	1.33 (15)
Bi1—Cl2	2.795 (12)	C2A—C3A	1.56 (14)
Bi1—Cl3	2.919 (11)	N2—C4	1.48 (6)
Bi1—Cl3^{i}	2.933 (13)	N2—C6	1.55 (6)
Cl3—Bi1^{ii}	2.933 (13)	C4—C5	1.45 (7)
Bi11—Cl42	2.528 (12)	C5—C6	1.56 (8)
Bi11—Cl41	2.572 (11)	N11—C11	1.46 (6)
Bi11—Cl11	2.626 (11)	N11—C31	1.47 (6)
Bi11—Cl21	2.781 (12)	C11—C21	1.51 (7)
Bi11—Cl31	2.890 (11)	C21—C31	1.43 (6)
Bi11—Cl31^{iii}	2.937 (11)	N21—C41	1.52 (9)
Cl31—Bi11^{iv}	2.937 (11)	N21—C61	1.48 (6)
N1—C3	1.44 (14)	C41—C51	1.55 (6)
N1—C1	1.45 (15)	C51—C61	1.45 (7)
C1—C2	1.67 (16)		
Cl4A—Bi1—Cl4	95.0 (5)	Cl3—Bi1—Cl3^{i}	88.29 (10)
Cl4A—Bi1—Cl1A	98.3 (9)	Bi1—Cl3—Bi1^{ii}	156.5 (5)
Cl4—Bi1—Cl1A	89.8 (9)	Cl42—Bi11—Cl41	92.7 (5)
Cl4A—Bi1—Cl1B	82.9 (10)	Cl42—Bi11—Cl11	91.8 (5)
Cl4—Bi1—Cl1B	98.2 (10)	Cl41—Bi11—Cl11	92.5 (4)
Cl4A—Bi1—Cl2	88.1 (5)	Cl42—Bi11—Cl21	95.8 (5)
Cl4—Bi1—Cl2	96.6 (5)	Cl41—Bi11—Cl21	88.3 (4)
Cl1A—Bi1—Cl2	170.6 (10)	Cl11—Bi11—Cl21	172.3 (4)
Cl1B—Bi1—Cl2	163.3 (11)	Cl42—Bi11—Cl31	87.7 (4)
Cl4A—Bi1—Cl3	88.8 (4)	Cl41—Bi11—Cl31	179.1 (4)
Cl4—Bi1—Cl3	175.2 (4)	Cl11—Bi11—Cl31	88.3 (4)
Cl1A—Bi1—Cl3	86.9 (9)	Cl21—Bi11—Cl31	90.9 (4)
Cl1B—Bi1—Cl3	79.4 (10)	Cl42—Bi11—Cl31^{iii}	176.6 (4)
Cl2—Bi1—Cl3	86.4 (4)	Cl41—Bi11—Cl31^{iii}	88.0 (4)
Cl4A—Bi1—Cl3^{i}	177.1 (4)	Cl11—Bi11—Cl31^{iii}	84.8 (4)
Cl4—Bi1—Cl3^{i}	87.9 (4)	Cl21—Bi11—Cl31^{iii}	87.6 (4)
Cl1A—Bi1—Cl3^{i}	81.8 (9)	Cl31—Bi11—Cl31^{iii}	91.70 (13)
Cl1B—Bi1—Cl3^{i}	96.8 (10)	Bi11—Cl31—Bi11^{iv}	146.8 (4)
Cl2—Bi1—Cl3^{i}	91.5 (4)		

**ABC**: $(C_3H_6NH_2)_2[BiCl_5]$ 240K (**I PHASE**)			
Bi1—Cl4^{v}	2.514 (2)	N1—C3	1.377 (18)
Bi1—Cl4	2.514 (2)	N1—C1	1.45 (2)
Bi1—Cl1	2.611 (4)	C1—C2	1.32 (2)
Bi1—Cl1^{v}	2.611 (4)	C2—C3	1.44 (3)
Bi1—Cl2^{v}	2.696 (5)	N2—C6	1.37 (2)
Bi1—Cl2	2.696 (5)	N2—C4	1.46 (2)
Bi1—Cl3^{i}	2.8706 (3)	C4—C5	1.30 (2)
Bi1—Cl3	2.8706 (3)	C5—C6	1.48 (3)
Cl4^{v}—Bi1—Cl4	92.77 (14)	Cl4—Bi1—Cl3^{i}	89.18 (7)

Cl4^{v}—Bi1—Cl1	85.67 (14)	Cl1—Bi1—Cl3^{i}	92.64 (12)
Cl4—Bi1—Cl1	96.39 (14)	Cl1^{v}—Bi1—Cl3^{i}	82.28 (11)
Cl4^{v}—Bi1—Cl1^{v}	96.39 (14)	Cl2^{v}—Bi1—Cl3^{i}	85.91 (15)
Cl4—Bi1—Cl1^{v}	85.67 (14)	Cl2—Bi1—Cl3^{i}	96.22 (14)
Cl1—Bi1—Cl1^{v}	14.8 (3)	Cl4^{v}—Bi1—Cl3	89.18 (7)
Cl4^{v}—Bi1—Cl2^{v}	95.72 (17)	Cl4—Bi1—Cl3	177.56 (7)
Cl4—Bi1—Cl2^{v}	85.06 (16)	Cl1—Bi1—Cl3	82.28 (11)
Cl1—Bi1—Cl2^{v}	177.95 (17)	Cl1^{v}—Bi1—Cl3	92.64 (12)
Cl1^{v}—Bi1—Cl2^{v}	165.1 (2)	Cl2^{v}—Bi1—Cl3	96.22 (14)
Cl4^{v}—Bi1—Cl2	85.06 (16)	Cl2—Bi1—Cl3	85.91 (15)
Cl4—Bi1—Cl2	95.72 (17)	Cl3^{i}—Bi1—Cl3	88.842 (11)
Cl1—Bi1—Cl2	165.1 (2)	Cl1^{v}—Cl1—Bi1	82.59 (13)
Cl1^{v}—Bi1—Cl2	177.95 (17)	Cl2^{v}—Cl2—Bi1	82.63 (14)
Cl2^{v}—Bi1—Cl2	14.7 (3)	Bi1^{vi}—Cl3—Bi1	180.0
Cl4^{v}—Bi1—Cl3^{i}	177.56 (7)		
**ABB**: $(C_3H_6NH_2)_2[BiBr_5]$ 200K (**II PHASE**)			
Bi1—Br4A	2.698 (4)	C2—C3	1.27 (13)
Bi1—Br4	2.705 (4)	N1A—C3A	1.46 (9)
Bi1—Br1B	2.763 (8)	N1A—C1A	1.53 (10)
Bi1—Br1A	2.776 (8)	C1A—C2A	1.43 (14)
Bi1—Br2	2.925 (3)	C2A—C3A	1.43 (11)
Bi1—Br3^{i}	3.055 (3)	N2—C6	1.45 (5)
Bi1—Br3	3.056 (3)	N2—C4	1.45 (5)
Br3—Bi1^{ii}	3.055 (3)	C4—C5	1.43 (6)
Bi11—Br42	2.689 (3)	C4—C6	2.02 (6)
Bi11—Br41	2.727 (3)	C5—C6	1.48 (6)
Bi11—Br11	2.788 (3)	N11—C11	1.45 (5)
Bi11—Br21	2.930 (3)	N11—C31	1.47 (5)
Bi11—Br31	3.008 (3)	C11—C21	1.48 (5)
Bi11—Br31^{iii}	3.059 (3)	C21—C31	1.51 (5)
Br31—Bi11^{iv}	3.059 (3)	N21—C41	1.43 (6)
N1—C1	1.36 (11)	N21—C61	1.47 (5)
N1—C3	1.59 (10)	C41—C51	1.45 (7)
C1—C2	1.78 (12)	C51—C61	1.48 (7)
C1—C3	1.98 (12)		
Br4A—Bi1—Br4	95.68 (15)	Br3^{i}—Bi1—Br3	86.85 (3)
Br4A—Bi1—Br1B	83.7 (3)	Bi1^{ii}—Br3—Bi1	159.26 (15)
Br4—Bi1—Br1B	98.5 (3)	Br42—Bi11—Br41	94.51 (13)
Br4A—Bi1—Br1A	99.3 (3)	Br42—Bi11—Br11	92.51 (12)
Br4—Bi1—Br1A	89.8 (2)	Br41—Bi11—Br11	93.01 (11)
Br4A—Bi1—Br2	88.66 (16)	Br42—Bi11—Br21	96.06 (12)
Br4—Bi1—Br2	95.47 (14)	Br41—Bi11—Br21	89.09 (10)
Br1B—Bi1—Br2	164.7 (3)	Br11—Bi11—Br21	170.99 (11)
Br1A—Bi1—Br2	170.0 (2)	Br42—Bi11—Br31	87.46 (12)
Br4A—Bi1—Br3^{i}	176.49 (13)	Br41—Bi11—Br31	177.32 (12)
Br4—Bi1—Br3^{i}	87.70 (13)	Br11—Bi11—Br31	88.71 (10)
Br1B—Bi1—Br3^{i}	96.7 (3)	Br21—Bi11—Br31	88.90 (10)
Br1A—Bi1—Br3^{i}	81.6 (3)	Br42—Bi11—Br31^{iii}	175.55 (11)
Br2—Bi1—Br3^{i}	90.10 (12)	Br41—Bi11—Br31^{iii}	88.16 (11)
Br4A—Bi1—Br3	89.81 (12)	Br11—Bi11—Br31^{iii}	83.78 (9)
Br4—Bi1—Br3	174.14 (13)	Br21—Bi11—Br31^{iii}	87.54 (10)
Br1B—Bi1—Br3	80.1 (2)	Br31—Bi11—Br31^{iii}	89.99 (3)
Br1A—Bi1—Br3	87.3 (2)	Bi11—Br31—Bi11^{iv}	150.79 (12)

Br2—Bi1—Br3	86.67 (12)		
**ABB**: $(C_3H_6NH_2)_2[BiBr_5]$ 250K (**I PHASE**)			
Bi1—Br4^{v}	2.6929 (14)	Br3—Bi1^{vi}	3.0452 (4)
Bi1—Br4	2.6930 (14)	N1—C1	1.45 (5)
Bi1—Br1	2.788 (3)	N1—C3	1.55 (4)
Bi1—Br1^{v}	2.788 (3)	C1—C1^{vii}	1.00 (8)
Bi1—Br2	2.870 (3)	C2—C3	1.37 (4)
Bi1—Br2^{v}	2.870 (3)	N2—C6	1.28 (5)
Bi1—Br3^{i}	3.0452 (4)	N2—C4	1.62 (4)
Bi1—Br3	3.0452 (4)	C4—C5	1.31 (4)
Br1—Br1^{v}	0.610 (10)	C5—C6	1.61 (5)
Br2—Br2^{v}	0.540 (16)		
Br4^{v}—Bi1—Br4	93.78 (9)	Br4—Bi1—Br3^{i}	89.78 (5)
Br4^{v}—Bi1—Br1	87.03 (10)	Br1—Bi1—Br3^{i}	91.70 (8)
Br4—Bi1—Br1	96.19 (10)	Br1^{v}—Bi1—Br3^{i}	83.09 (8)
Br4^{v}—Bi1—Br1^{v}	96.19 (10)	Br2—Bi1—Br3^{i}	94.35 (13)
Br4—Bi1—Br1^{v}	87.03 (10)	Br2^{v}—Bi1—Br3^{i}	86.95 (13)
Br1—Bi1—Br1^{v}	12.6 (2)	Br4^{v}—Bi1—Br3	89.78 (5)
Br4^{v}—Bi1—Br2	86.30 (15)	Br4—Bi1—Br3	176.32 (5)
Br4—Bi1—Br2	94.17 (14)	Br1—Bi1—Br3	83.09 (8)
Br1—Bi1—Br2	168.02 (19)	Br1^{v}—Bi1—Br3	91.70 (8)
Br1^{v}—Bi1—Br2	177.17 (11)	Br2—Bi1—Br3	86.95 (12)
Br4^{v}—Bi1—Br2^{v}	94.17 (14)	Br2^{v}—Bi1—Br3	94.35 (13)
Br4—Bi1—Br2^{v}	86.30 (15)	Br3^{i}—Bi1—Br3	86.635 (13)
Br1—Bi1—Br2^{v}	177.17 (11)	Br1^{v}—Br1—Bi1	83.72 (10)
Br1^{v}—Bi1—Br2^{v}	168.02 (19)	Br2^{v}—Br2—Bi1	84.61 (16)
Br2—Bi1—Br2^{v}	10.8 (3)	Bi1^{vi}—Br3—Bi1	180.0
Br4^{v}—Bi1—Br3^{i}	176.32 (5)		

Symmetry code(s): (i) −*x*, *y*−1/2, −*z*; (ii) −*x*, *y*+1/2, −*z*; (iii) −*x*+1, *y*+1/2, −*z*+1; (iv) −*x*+1, *y*−1/2, −*z*+1; (v) *x*, −*y*+3/2, *z*; (vi) −*x*, −*y*+2, −*z*; (vii) *x*, −*y*+1/2, *z*.

Table S3. Selected hydrogen-bond parameters.

D—H⋯*A*	*D*—H (Å)	H⋯*A* (Å)	*D*⋯*A* (Å)	*D*—H⋯*A* (°)
**ABC**: $(C_3H_6NH_2)_2[BiCl_5]$ 140K (**III PHASE**)				
N1—H1D⋯$Cl2^{i}$	0.89	2.44	3.30 (6)	162.0
N2—H2D⋯$Cl21^{iii}$	0.89	2.29	3.17 (4)	169.7
N2—H2C⋯$Cl31^{iv}$	0.89	2.32	3.20 (3)	170.2
N11—H11D⋯Cl11	0.89	2.60	3.17 (4)	123.2
N11—H11C⋯Cl31	0.89	2.39	3.22 (4)	155.4
N21—H21D⋯$Cl3^{v}$	0.89	2.46	3.34 (5)	171.7
C1—H1B⋯$Cl41^{vi}$	0.97	2.66	3.24 (8)	119.2
C3—H3B⋯$Cl1A^{v}$	0.97	1.96	2.61 (11)	122.1
**ABC**: $(C_3H_6NH_2)_2[BiCl_5]$ 200K (**II PHASE**)				
N1—H1D⋯$Cl2^{i}$	0.89	2.47	3.37 (10)	155
N2—H2D⋯$Cl21^{iii}$	0.89	2.27	3.22 (5)	166
N2—H2C⋯$Cl31^{iv}$	0.89	2.27	3.23 (4)	172
N11—H11D⋯Cl11	0.89	2.58	3.20 (5)	122
N11—H11C⋯Cl31	0.89	2.40	3.29 (5)	152
N21—H21D⋯$Cl3^{v}$	0.89	2.35	3.31 (5)	169
C1—H1B⋯$Cl41^{vi}$	0.97	2.87	3.38 (10)	113
C3—H3B⋯$Cl1A^{v}$	0.97	2.00	2.76 (12)	133
**ABC**: $(C_3H_6NH_2)_2[BiCl_5]$ 240K (**II PHASE**)				
C1—H1D⋯$Cl1^{vii}$	0.96	2.41	3.29 (2)	151.6
C5—H5B⋯$Cl2^{viii}$	0.96	2.60	3.39 (2)	139.5
N1—H1B⋯$Cl3^{ii}$	0.90	2.57	3.35 (2)	144.9
N2—H2D⋯$Cl2^{ix}$	0.90	2.57	3.31 (2)	141.1
**ABB**: $(C_3H_6NH_2)_2[BiBr_5]$ 200K (**II PHASE**)				
N1—H1C⋯$Br2^{i}$	0.89	2.77	3.56 (8)	148.8
N1—H1D⋯$Br4A^{ii}$	0.89	2.70	3.56 (8)	161.5
C2—H2A⋯Br42	0.97	2.62	3.57 (10)	165.3
C3—H3B⋯$Br1A^{v}$	0.97	2.04	2.86 (10)	140.6
N2—H2C⋯$Br21^{iii}$	0.89	2.44	3.32 (3)	171.3
N2—H2D⋯$Br31^{iv}$	0.89	2.58	3.44 (3)	163.0
N11—H11C⋯Br31	0.89	2.73	3.43 (3)	135.6
N11—H11D⋯Br11	0.89	2.73	3.40 (4)	133.5
N21—H21C⋯$Br2^{i}$	0.89	2.63	3.47 (5)	156.9
N21—H21D⋯$Br3^{v}$	0.89	2.63	3.51 (4)	167.9
**ABB**: $(C_3H_6NH_2)_2[BiBr_5]$ 250K (**I PHASE**)				
N1—H1B⋯$Br3^{ii}$	0.79	2.76	3.49 (4)	153.9
C1—H1D⋯$Br1^{vii}$	1.00	2.58	3.49 (4)	150.8
C3—H3B⋯$Br1^{ii}$	1.00	2.78	3.58 (4)	137.7
N2—H2D⋯$Br2^{ix}$	0.94	2.70	3.48 (3)	141.5
C5—H5A⋯$Br4^{x}$	1.00	3.03	3.55 (4)	113.2
C5—H5B⋯$Br2^{viii}$	1.00	2.63	3.49 (4)	144.3

Thermal analysis

Differential scanning calorimetry (DSC) heating traces were obtained using a Mettler Toledo DSC 3 differential scanning calorimeter calibrated using n-octane and indium. Hermetically sealed Al pans with the polycrystalline material were prepared in an air atmosphere. The measurements were performed between 120 and 300 K. Simultaneous thermogravimetric analysis (TGA) and differential scanning calorimetry (DSC) were carried out on a Mettler Toledo TGA/DSC 3+ instruments in the temperature range 300-900 K with a ramp rate 5 K·min^{-1} in the nitrogen atmosphere (flow rate: 1 dm^3·h^{-1}).

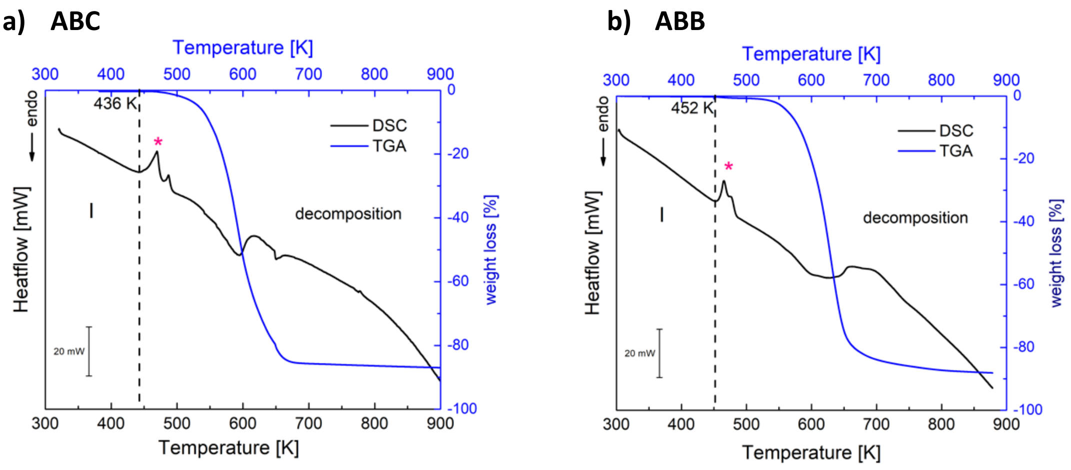

Figure S3. The TGA and DSC traces for a) **ABC** and b) **ABB** crystal measured on heating (mass of the sample m =16.535 and 14.846 mg, heating/cooling rate 5 K/min).

Table S4. Thermodynamics parameters, Δ*H* and Δ*S*, and parameter *N* calculated based on Boltzmann relationship.

	**ABC**				**ABB**	
	I-II		II-III		I-II	
	cooling	heating	cooling	heating	cooling	heating
M [g/mol]	502.5	502.5	502.5	502.5	724.7	724.7
$T_{P.T.}$ [K]	231.7	233.6	178.7	182.3	223.6	224.5
Δ*H* [J/g]	6.0	5.8	1.2	1.1	3.9	4.0
Δ*H* [J/mol]	3019.7	2909.2	577.8	567.8	2811.8	2862.6
Δ*S* [J/K·mol]	13.0	12.5	3.2	3.1	12.6	12.7
N	4.8	4.5	1.5	1.5	4.5	4.6

Electric properties

The complex dielectric permittivity, ε* = ε′ − iε″, measurements were conducted on **ABC** and **ABB** in the form of polycrystalline pellets on an Agilent E4980A Precision LCR Meter between 100 and 350 K in the frequency range between 200 Hz and 2 MHz. The silver electrodes were painted on both opposite sides of the pellets. The overall errors of ε′ and ε″ were less than 5%. The temperature was stabilized and controlled using an INSTEC STC200, and the cooling and heating runs were measured with 5K/min ramp. The pyroelectric properties were tested with a Keithley 6517D electrometer/high resistance meter between 180 and 300 K, with a temperature ramp of 2 K min^{-1}. The ferroelectric hysteresis loops were obtained by using a Sawyer–Tower circuit Precision Premier II (Radiant Technologies, Inc.), with the setup of the drive profile type as f =0.5 Hz and standard bipolar signal. The diameter of the pellet was of the order of 5 mm, and its thickness of 1 mm. The surfaces of the polycrystalline pellets were coated with silver conductive paint (Electron Microscopy Sciences, 503).

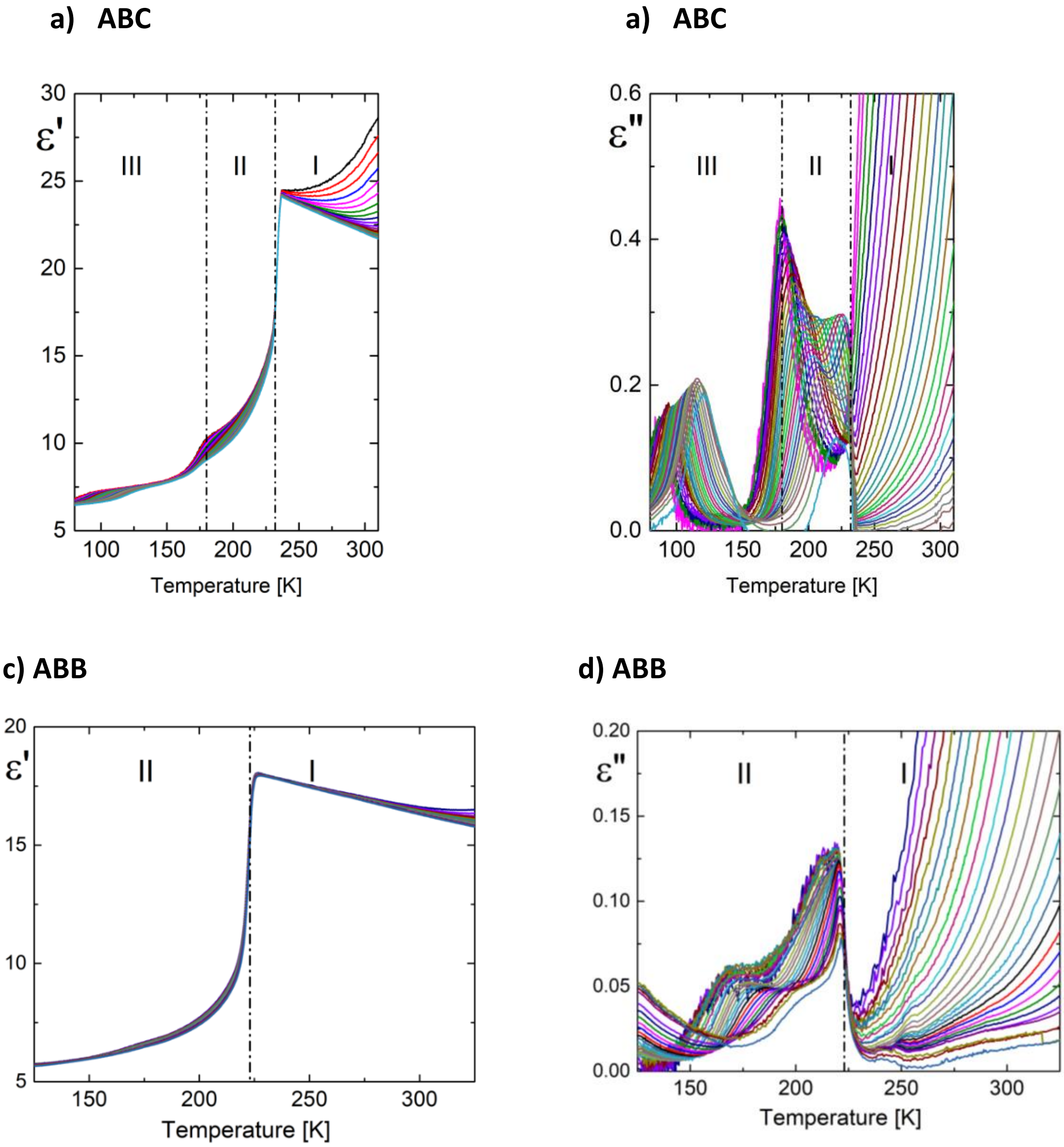

Figure S4. The temperature dependence of the real (a) – **ABC** and c) - **ABB**) and imaginary (b) – **ABC** and d) - **ABB**) parts of the complex electric permittivity at several frequencies from the range 600 Hz - 2 MHz.

Figure S3 presents the dielectric response measured on polycrystalline pellets in temperatures from 90 to 350 K and 120 to 350 K for **ABC** and **ABB**, respectively. In both cases, the phase transitions from the **I** to **II** phases are illustrated as a rapid jump from a high to a low dielectric state. Based on structural analysis and calorimetric, one can conclude that the step-wise change in permittivity at **I→II** PT is characteristic of the order-disorder type transition mechanism. Next, two relaxation processes are observed within the intermediate (II) phase, labeled as 2a and 2b in FigureS4. A double Cole-Cole[4] model was fitted to these temperatures. The fitting results are shown in Figs. S5 and S6. In the case of **ABC**, further cooling of the sample (up to 200 K) causes a transition from the area where two relaxation processes exist to a phase where the change in the orientation of molar dipoles can be described by a single Cole-Cole equation (labelled as 2 in Fig S4):

$$\varepsilon^{*}=\varepsilon'-i\varepsilon''=\varepsilon_{\infty}+\frac{\varepsilon_{0}-\varepsilon_{\infty}}{1+(i\omega\tau)^{(1-\alpha)}} \qquad (1)$$

where ε_0 and ε_∞ are the low- and high-frequency electric permittivity limits, respectively, ω is the angular frequency, τ macroscopic relaxation time, and α is the parameter describing the distribution of relaxation times. In addition, a subtle change associated with the next phase transition (**II→III**) is observed in this crystal. In the low-temperature phase (**III**), a new dielectric relaxation appears (marked as 1 in Figure S4), to which the Cole-Cole model was fitted.

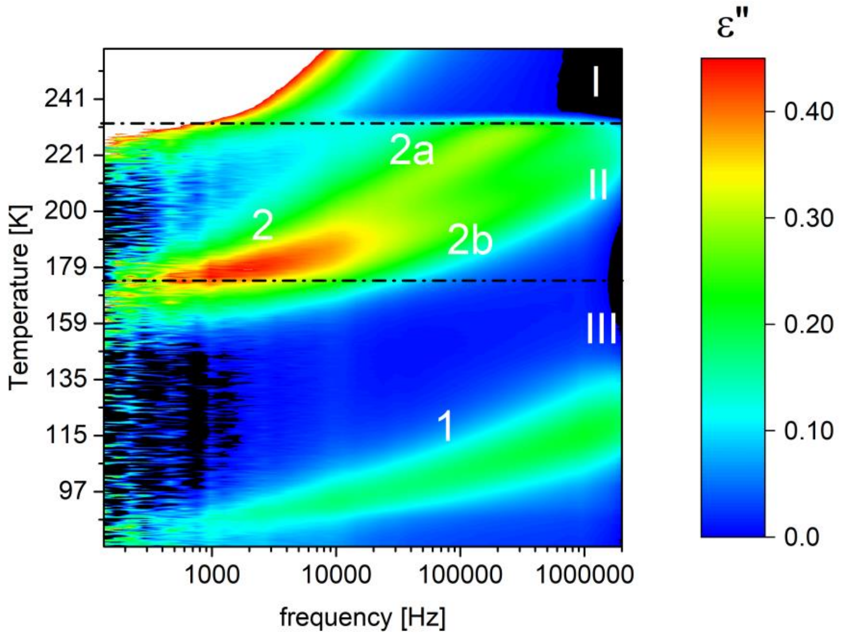

Figure S5. The temperature and frequency dependence of ε'' measured for **ABC** in frequency ranges from 135 Hz to 2 MHz. White indicates the ac conductivity contribution, while black artificial values of ε''.

The value of the activation energies, E_a, were estimated from the Arrhenius relationship:

$$\tau = C\exp\left(\frac{-E_a}{RT}\right) \qquad (2)$$

where C is constant, R is a gas constant, and T is a temperature. The ln τ *versus* 1000/T plot is shown in FigureS7.

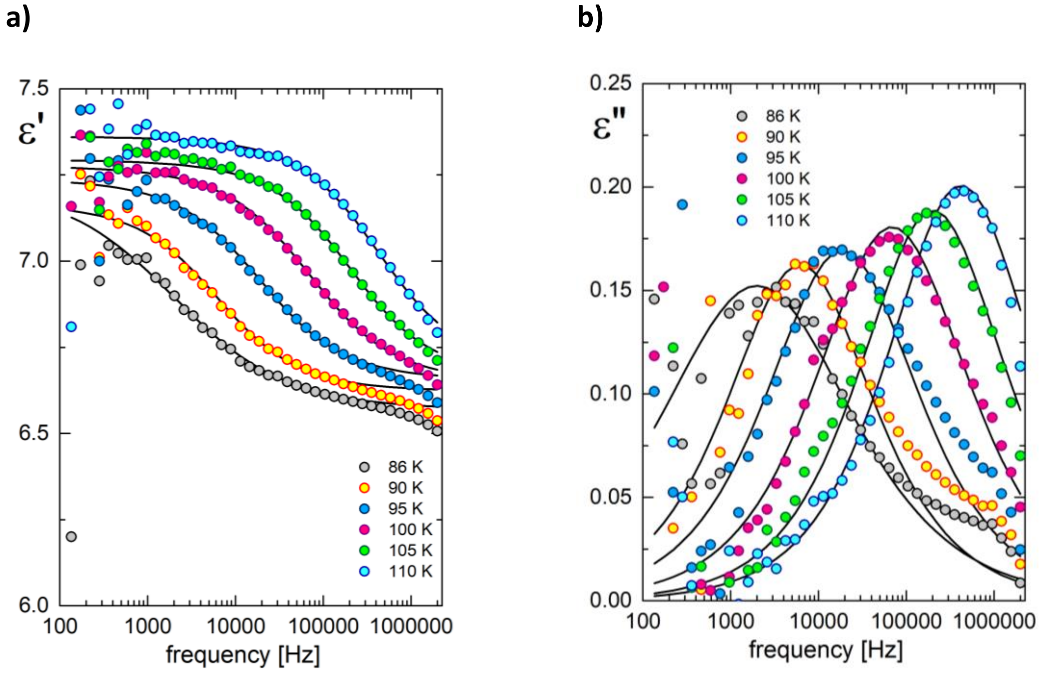

Figure S6. The frequency dependence of the real (a) and imaginary (b) parts of the complex electric permittivity at several temperatures in phase **III** for **ABC**.

a)

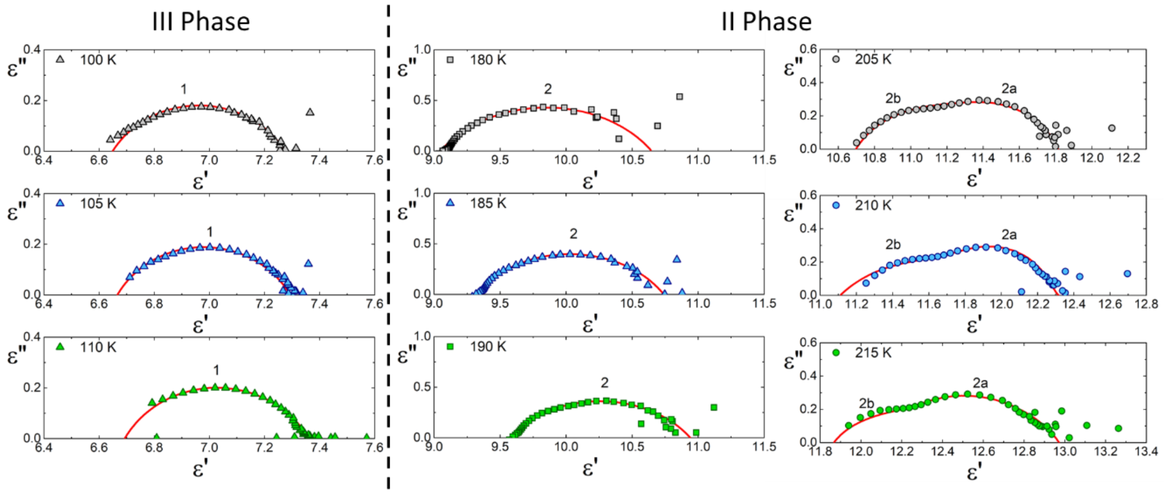

b)

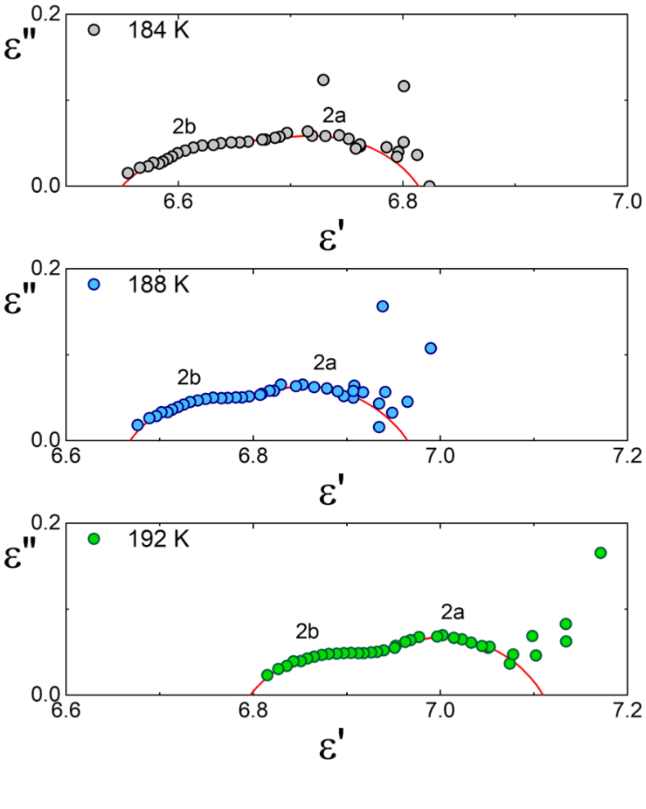

Figure S7. Cole–Cole plots for selected temperatures for a) **ABC** and b) **ABB** in frequency ranges from 135 Hz to 2 MHz.

a)

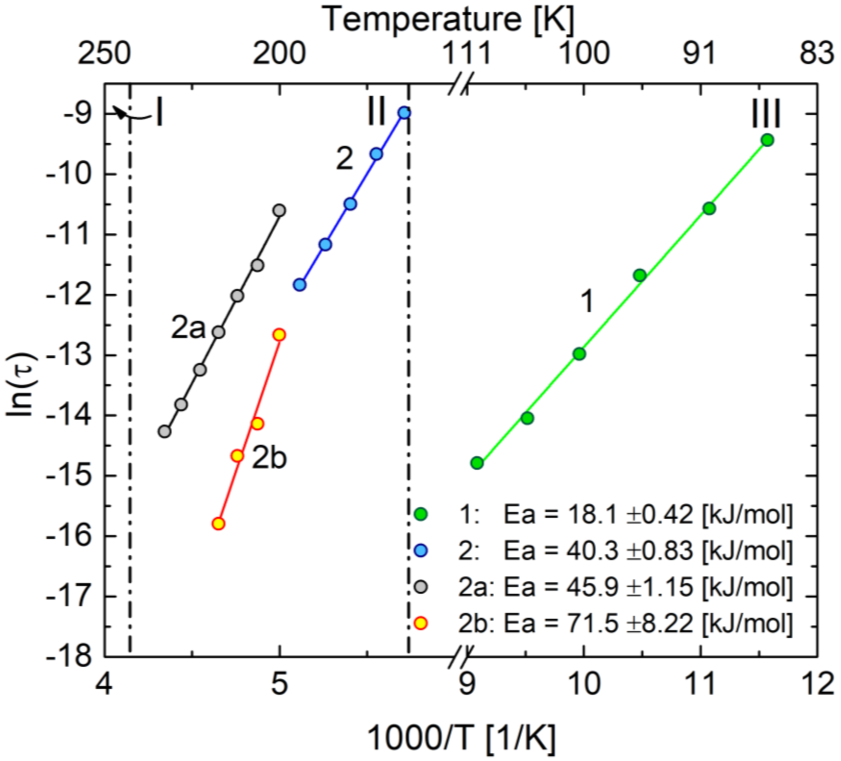

b)

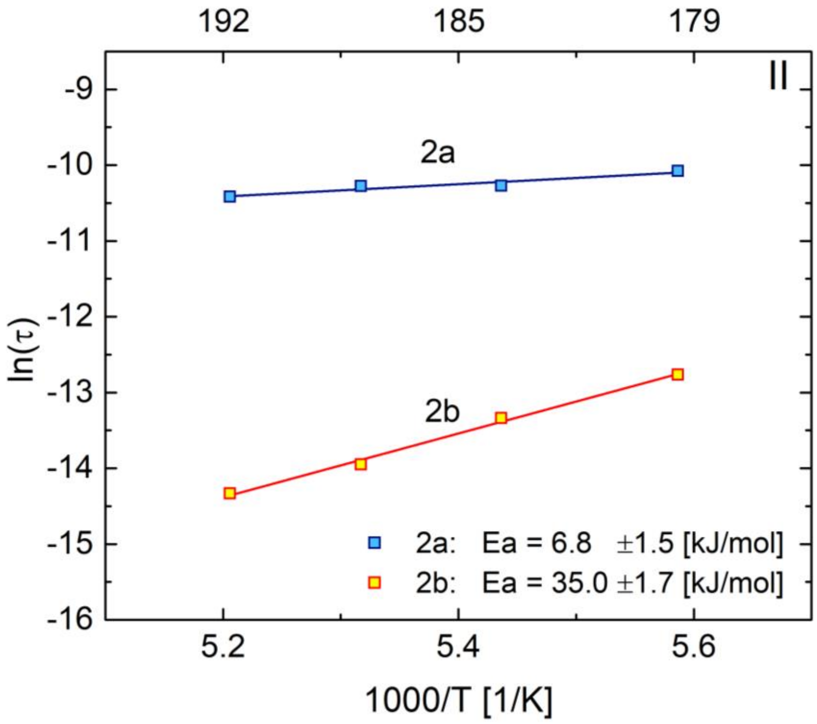

Figure S8. Arrhenius plot of relaxation time measured for a) **ABC** in the region of two phases (**III** and **II**), and b) **ABB** measured only within one phase **II**.

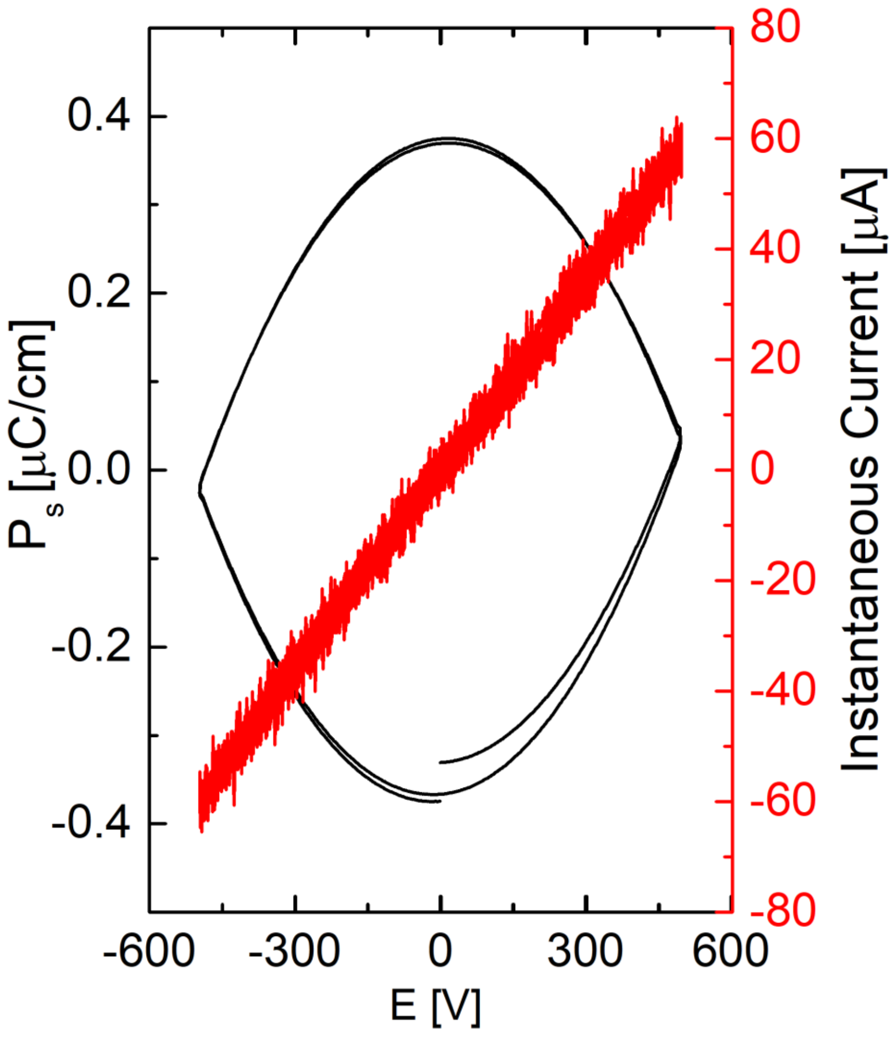

Figure S9. Polarization–electric field (*P*–*E*) hysteresis loops measured for RT (black line, max. electric field of 5.21 kV/cm, f = 0.5 Hz). b) IC–E (instantaneous current (mA) density–electric field) red line.

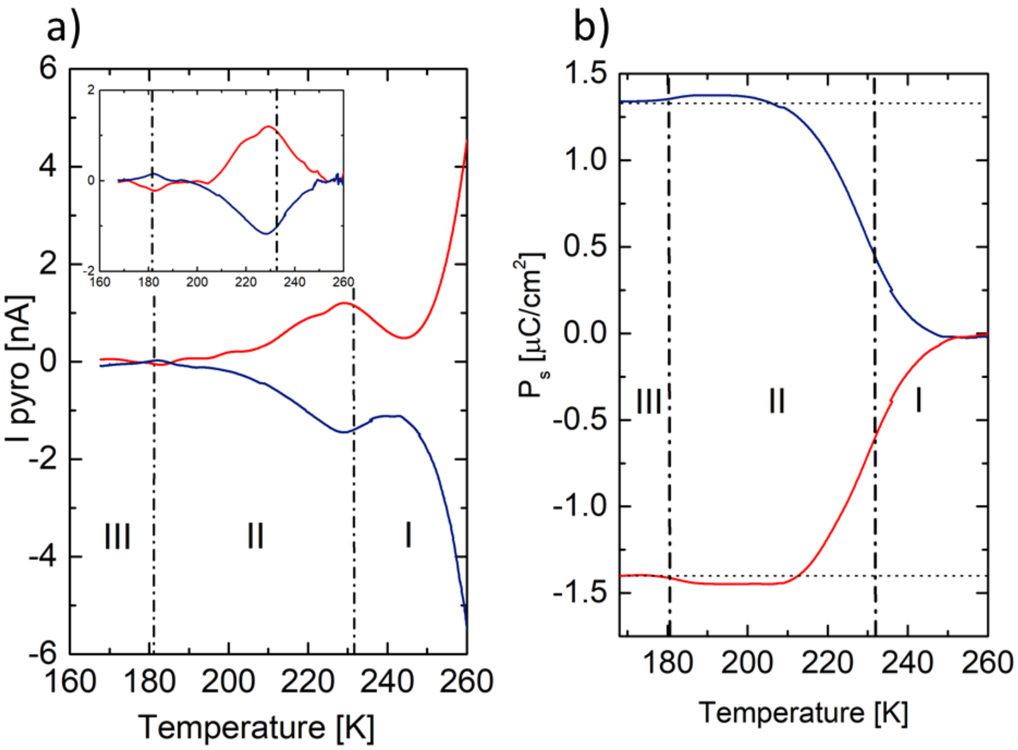

Figure S10. a) Temperature dependence of I_{pyro} measured for **ABC** sample after applying the DC electric field (±3.33 kV/cm, d = 1.2 mm) in phase **I** and then cooling the sample to phase **II**. The measurements of I_{pyro} were carried out during heating, b) Polarization P_s(T) determined by the integration of the pyroelectric current.

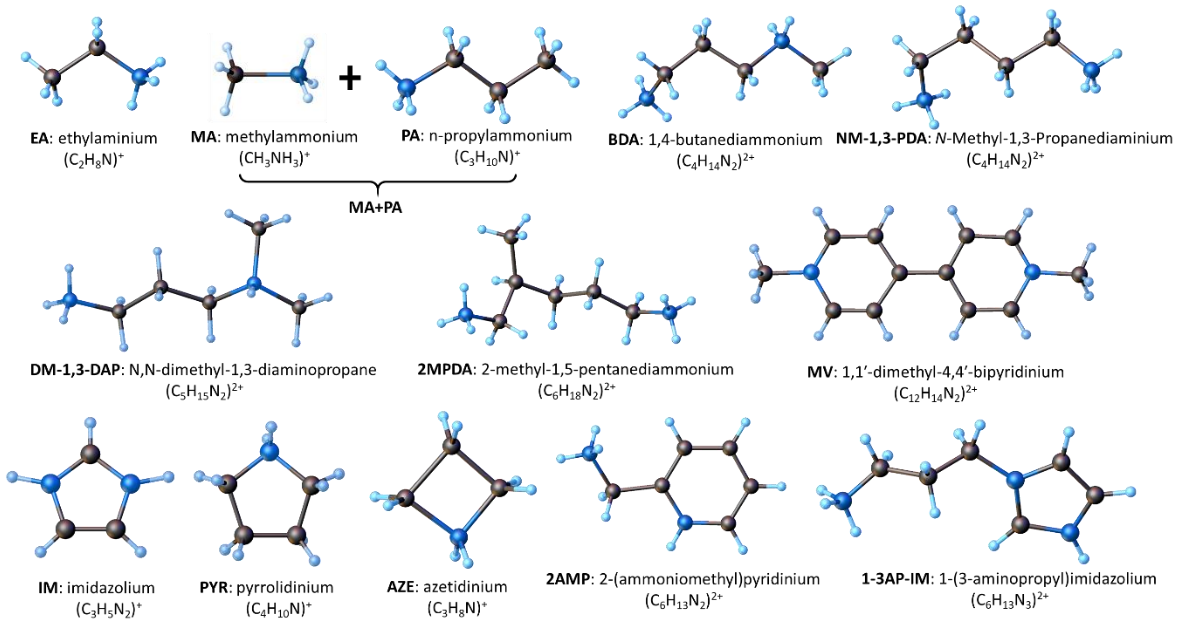

Figure S11. Molecular structure of the organic cations creating ferroelectric compounds among halobismuthates(III) and haloantimonates(III) with R_2MX_3 stoichiometry.

Table S5. Compilation of the Most Important Parameters Characterizing Structural and Spontaneous Polarization Properties for Ferroelectric among halobismuthates(III) and haloantimonates(III) with R_2MX_3 stoichiometry.

Compound stoichiometry	Space group	Anionic structure	T_c [K]	P_s [μC/cm^2]	Ref
**(AZE)**$_2$[BiCl$_5$]	*P*2$_1$	deformed *cis*-mode chain	233	1.61 (232 K)	*
**(AZE)**$_2$[BiBr$_5$]	*P*2$_1$	deformed *cis*-mode chain	224.5		*
(**2AMP**)[SbI$_5$]	*Pb*2$_1$*a*	deformed *cis*-mode chain	360	4.0 (338 K)	5
(**PYR**)$_2$[SbCl$_5$]	*Pn*	deformed *cis*-mode chain	252	0.16 (217 K)	6
(**MV**)[BiBr$_5$]	*P*2$_1$	*trans* mode chain	243	n/a	7
(**MV**)[BiCl$_2$I$_5$]	*P*4*nc*	*trans* mode chain	-	15 (292 K)	8
(**EA**)$_2$[BiCl$_5$]	*Aba*$_2$	deformed *cis*-mode chain	190	1.4 (180 K)	9
(**EA**)$_2$[BiBr$_5$]	*Pca*2$_1$	*cis*-mode chain	160	$0.5\cdot10^{-2}$ (156 K)	10
(**IM**)$_2$[SbCl$_5$]	*Pna*2$_1$	atypical *cis*-mode chain	180	0.8 (112 K)	11
(**DM-1,3-DAP**)[SbCl$_5$]	*Pc*	*cis*-mode chain	143	1.36 (119 K)	12
(**NM-1,3-PDA**)[BiCl$_5$]	*Pna*2$_1$	*cis*-mode chain	376	2.38 (353 K)	13
(**NM-1,3-PDA**)[BiCl$_5$]	*Pna*2$_1$	*cis*-mode chain	377	10 (374 K)	14
(**NM-1,3-PDA**)[BiBr$_5$]	*Pna*2$_1$	*cis*-mode chain	318	4.5 (313 K)	14
(**1-3AP-IM**)[SbBr$_5$]	*P*2$_1$	*cis*-mode chain	230	6.86 (195 K)	15
(**BDA**)[BiI$_5$]	*P*2$_1$	*cis*-mode chain	365	1.35 (345 K)	16
(**MA+PA**)[SbBr$_5$]	*Pna*2$_1$	*cis*-mode chain	238	2.9 (360 K)	17
(**2MPDA**)[SbI$_5$]	*Pna*2$_1$	*cis*-mode chain		12.5 (RT)	18

*this paper

Calculation details

Total energy calculations were performed using *ab initio* Density Functional Theory (DFT) as implemented in the CRYSTAL17[19,20] package, designed for use in modelling crystalline solids and employed the London-type empirical correction in the (D3) variant for dispersion interactions as proposed by Grimme[21–24] including three-body dispersion contributions with fast analytical gradients together with the vibrational harmonic frequency calculations. The structural data (starting geometry) were taken from the X-ray crystal structures of **ABC** and **ABB** from this present study. The periodic *ab initio* calculations were performed utilizing the DFT-D3 methods with the range-separated (short range corrected) hybrid functional, screened-Coulomb PBE functional combined with PBE correlation: HSE06-D3[25,26] with the two shrinking factors (6',6') to generate a commensurate grid of k-points in reciprocal space, following the Monkhorst–Pack[27] net method. All quantum-mechanical condensed matter simulations including: a single point energy, the geometric optimization of crystal structures and lattice parameters, the spontaneous polarization (P_s), the electronic band structure (EBS) and the density of states (DOS) were carried out with the consistent gaussian basis sets of triple-zeta valence with polarization quality and BSSE-correction for solid-state calculations (pob_TZVP_rev2)[28–31] proposed by Vilela-Oliveira, Peintinger, Laun and Bredow in the second revision version. The electronic band structure was generated according to the procedure in the CRYSTAL17 program and the SeeK-path[32] program was used to determine the k-points along a path within the first Brillouin zone including the surface in reciprocal space. The EBS and DOS data from calculations were visualized *a posteriori* in the Gnuplot[33] program.

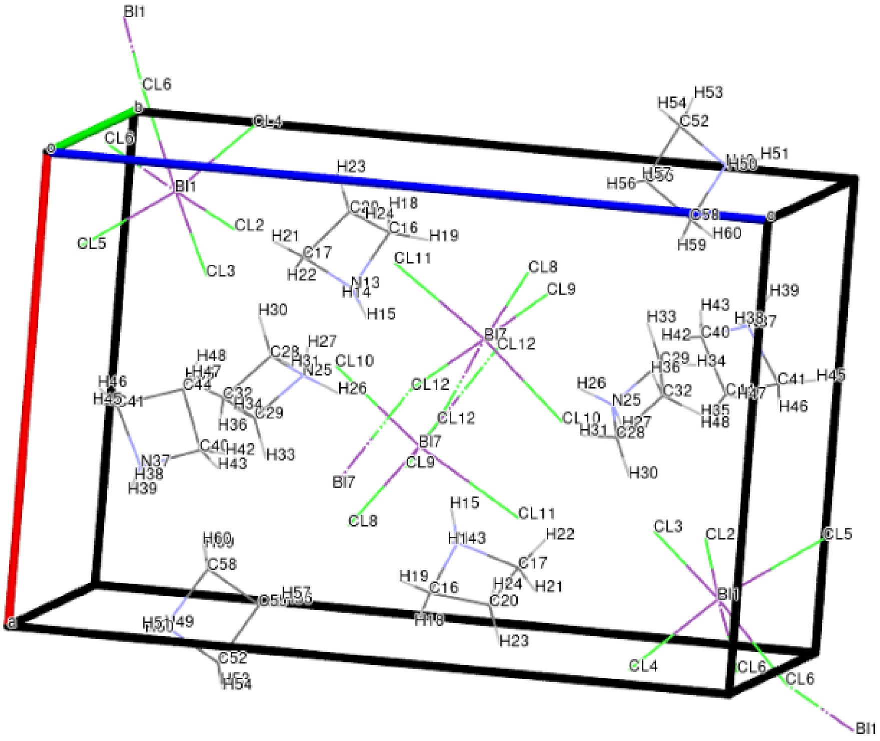

Figure S12. Structure and geometric parameters of the **ABC** crystal after optimization at the DFT (HSE06-D3/pob_TZVP_rev2) method level.

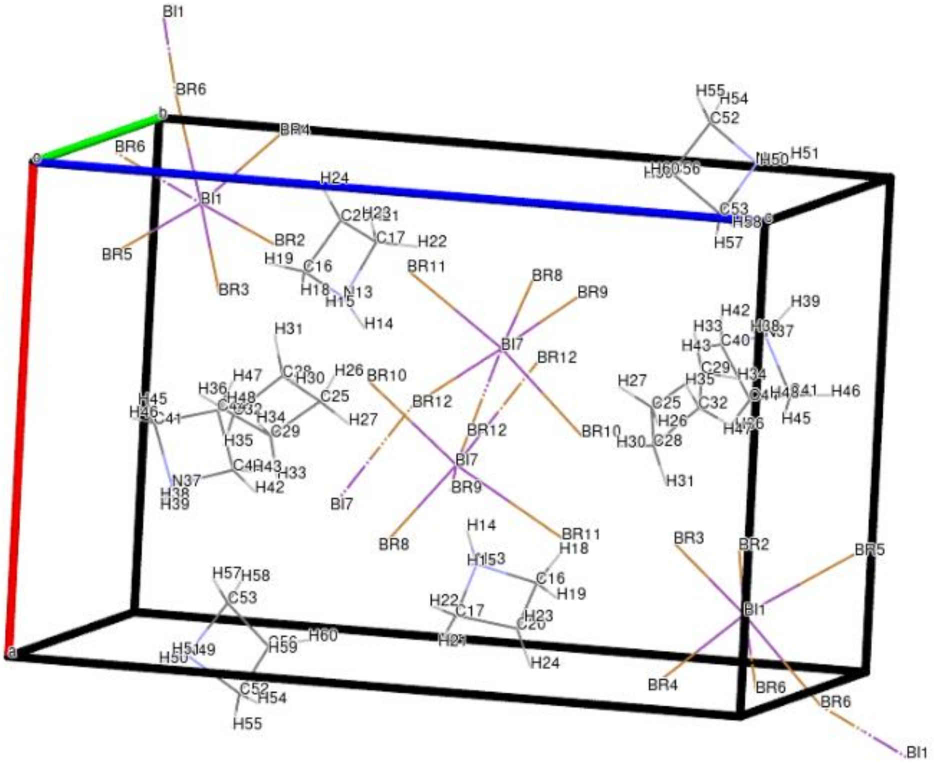

Figure S13. Structure and geometric parameters of the **ABB** crystal after optimization at the DFT (HSE06-D3/pob_TZVP_rev2) method level.

Table S6. Geometric and lattice parameters of the **ABC** crystal after optimization at the DFT (HSE06-D3/pob_TZVP_rev2) method level.

Bonds	[Å]	Angles	[deg]	Dihedrals	[deg]
Bi1-Cl3	2.546	Cl3-Bi1-Cl4	87.87	Cl3-Bi1-Cl6-Bi1	152.96
Bi1-Cl4	2.582	Cl3-Bi1-Cl2	94.56	Cl4-Bi1-Cl6-Bi1	119.49
Bi1-Cl2	2.532	Cl3-Bi1-Cl5	95.91	Cl2-Bi1-Cl6-Bi1	18.42
Bi1-Cl5	2.756	Cl3-Bi1-Cl6	175.29	Cl5-Bi1-Cl6-Bi1	-69.95
Bi1-Cl6	2.883	Cl3-Bi1-Cl6	88.66	Cl6-Bi1-Cl6-Bi1	-161.15
Bi1-Cl6	2.903	Cl4-Bi1-Cl2	101.03	Cl8-Bi7-Cl12-Bi7	-165.1
Cl6-Bi1	2.903	Cl4-Bi1-Cl5	169.64	Cl9-Bi7-Cl12-Bi7	75.88
Bi1-Cl3	2.546	Cl4-Bi1-Cl6	88.2	Cl10-Bi7-Cl12-Bi7	-72.93
Bi1-Cl4	2.582	Cl4-Bi1-Cl6	79.3	Cl11-Bi7-Cl12-Bi7	98.95
Bi1-Cl2	2.532	Cl2-Bi1-Cl5	88.32	Cl12-Bi7-Cl12-Bi7	11.7
Bi1-Cl5	2.756	Cl2-Bi1-Cl6	88.74	H14-N13-C16-H18	-16.47
Bi1-Cl6	2.883	Cl2-Bi1-Cl6	176.77	H14-N13-C16-H19	112.28
Bi1-Cl6	2.903	Cl5-Bi1-Cl6	87.54	H14-N13-C16-C20	-132.25
Cl6-Bi1	2.903	Cl5-Bi1-Cl6	91.12	H15-N13-C16-H18	-144.97
Bi7-Cl8	2.507	Cl6-Bi1-Cl6	88.06	H15-N13-C16-H19	-16.22
Bi7-Cl12	2.873	Bi1-Cl6-Bi1	159.23	H15-N13-C16-C20	99.25
Bi7-Cl9	2.554	Cl8-Bi7-Cl12	87.76	C17-N13-C16-H18	99.15
Bi7-Cl10	2.616	Cl8-Bi7-Cl9	92.86	C17-N13-C16-H19	-132.1
Bi7-Cl11	2.761	Cl8-Bi7-Cl10	92.11	C17-N13-C16-C20	-16.63
Bi7-Cl12	2.926	Cl8-Bi7-Cl11	95.99	H14-N13-C17-C20	131.8
Cl12-Bi7	2.926	Cl8-Bi7-Cl12	176.74	H14-N13-C17-H21	16.44
Bi7-Cl8	2.507	Cl12-Bi7-Cl9	178.71	H14-N13-C17-H22	-113.35
Bi7-Cl12	2.873	Cl12-Bi7-Cl10	88.56	H15-N13-C17-C20	-99.31
Bi7-Cl9	2.554	Cl12-Bi7-Cl11	91.14	H15-N13-C17-H21	145.33
Bi7-Cl10	2.616	Cl12-Bi7-Cl12	91.64	H15-N13-C17-H22	15.54
Bi7-Cl11	2.761	Cl9-Bi7-Cl10	92.54	C16-N13-C17-C20	16.29
Bi7-Cl12	2.926	Cl9-Bi7-Cl11	87.68	C16-N13-C17-H21	-99.06
N13-H14	0.888	Cl9-Bi7-Cl12	87.8	C16-N13-C17-H22	131.14
N13-H15	0.889	Cl10-Bi7-Cl11	171.88	N13-C16-C20-C17	16.48
N13-C16	1.505	Cl10-Bi7-Cl12	84.68	N13-C16-C20-H23	132.06
N13-C17	1.508	Cl11-Bi7-Cl12	87.22	N13-C16-C20-H24	-99.16
C16-H18	0.969	Bi7-Cl12-Bi7	146.02	H18-C16-C20-C17	-99.51
C16-H19	0.973	H14-N13-H15	111.26	H18-C16-C20-H23	16.07
C16-C20	1.490	H14-N13-C16	113.57	H18-C16-C20-H24	144.86
C17-C20	1.521	H14-N13-C17	113.7	H19-C16-C20-C17	132.16
C17-H21	0.964	H15-N13-C16	113.62	H19-C16-C20-H23	-112.26
C17-H22	0.970	H15-N13-C17	113.92	H19-C16-C20-H24	16.53
C20-H23	0.965	C16-N13-C17	89.2	N13-C17-C20-C16	-16.46
C20-H24	0.969	N13-C16-H18	114.07	N13-C17-C20-H23	-132.02
N13-H14	0.888	N13-C16-H19	113.84	N13-C17-C20-H24	99.09
N13-H15	0.889	N13-C16-C20	88.99	H21-C17-C20-C16	99.04
N13-C16	1.505	H18-C16-H19	110.97	H21-C17-C20-H23	-16.52
N13-C17	1.508	H18-C16-C20	113.85	H21-C17-C20-H24	-145.4

C16-H18	0.969	H19-C16-C20	113.6	H22-C17-C20-C16	-131.43
C16-H19	0.973	N13-C17-C20	87.75	H22-C17-C20-H23	113.01
C16-C20	1.490	N13-C17-H21	114.15	H22-C17-C20-H24	-15.87
C17-C20	1.521	N13-C17-H22	113.71	H26-N25-C28-H30	127.75
C17-H21	0.964	C20-C17-H21	113.98	H26-N25-C28-H31	-0.68
C17-H22	0.970	C20-C17-H22	113.6	H26-N25-C28-C32	-116.19
C20-H23	0.965	H21-C17-H22	111.73	H27-N25-C28-H30	0.45
C20-H24	0.969	C16-C20-C17	89.26	H27-N25-C28-H31	-127.98
N25-H26	0.888	C16-C20-H23	113.61	H27-N25-C28-C32	116.51
N25-H27	0.892	C16-C20-H24	113.59	C29-N25-C28-H30	-115.64
N25-C28	1.474	C17-C20-H23	113.63	C29-N25-C28-H31	115.93
N25-C29	1.501	C17-C20-H24	113.68	C29-N25-C28-C32	0.42
C28-H30	0.966	H23-C20-H24	111.46	H26-N25-C29-C32	116.1
C28-H31	0.976	H26-N25-H27	110.71	H26-N25-C29-H33	0.46
C28-C32	1.470	H26-N25-C28	113.69	H26-N25-C29-H34	-128.59
C29-C32	1.461	H26-N25-C29	113.79	H27-N25-C29-C32	-116.12
C29-H33	0.971	H27-N25-C28	113.02	H27-N25-C29-H33	128.23
C29-H34	0.972	H27-N25-C29	113.45	H27-N25-C29-H34	-0.81
C32-H35	0.966	C28-N25-C29	90.97	C28-N25-C29-C32	-0.42
C32-H36	0.974	N25-C28-H30	114.23	C28-N25-C29-H33	-116.07
N25-H26	0.888	N25-C28-H31	113.39	C28-N25-C29-H34	114.89
N25-H27	0.892	N25-C28-C32	88.49	N25-C28-C32-C29	-0.43
N25-C28	1.474	H30-C28-H31	110.94	N25-C28-C32-H35	-117.39
N25-C29	1.501	H30-C28-C32	114.29	N25-C28-C32-H36	115.96
C28-H30	0.966	H31-C28-C32	113.93	H30-C28-C32-C29	115.57
C28-H31	0.976	N25-C29-C32	87.77	H30-C28-C32-H35	-1.38
C28-C32	1.470	N25-C29-H33	114	H30-C28-C32-H36	-128.03
C29-C32	1.461	N25-C29-H34	114.17	H31-C28-C32-C29	-115.44
C29-H33	0.971	C32-C29-H33	114.26	H31-C28-C32-H35	127.61
C29-H34	0.972	C32-C29-H34	113.93	H31-C28-C32-H36	0.95
C32-H35	0.966	H33-C29-H34	111	N25-C29-C32-C28	0.43
C32-H36	0.974	C28-C32-C29	92.77	N25-C29-C32-H35	117.22
N37-H38	0.887	C28-C32-H35	113.21	N25-C29-C32-H36	-116.16
N37-H39	0.889	C28-C32-H36	113.14	H33-C29-C32-C28	115.83
N37-C40	1.417	C29-C32-H35	113.4	H33-C29-C32-H35	-127.38
N37-C41	1.500	C29-C32-H36	112.91	H33-C29-C32-H36	-0.76
C40-H42	0.969	H35-C32-H36	110.47	H34-C29-C32-C28	-115.11
C40-H43	0.971	H38-N37-H39	110.76	H34-C29-C32-H35	1.68
C40-C44	1.454	H38-N37-C40	112.52	H34-C29-C32-H36	128.3
C41-C44	1.543	H38-N37-C41	113.42	H38-N37-C40-H42	0.04
C41-H45	0.964	H39-N37-C40	112.45	H38-N37-C40-H43	128.12
C41-H46	0.970	H39-N37-C41	112.95	H38-N37-C40-C44	-116.12
C44-H47	0.968	C40-N37-C41	93.82	H39-N37-C40-H42	-125.86
C44-H48	0.972	N37-C40-H42	113.83	H39-N37-C40-H43	2.21
N37-H38	0.887	N37-C40-H43	113.75	H39-N37-C40-C44	117.97
N37-H39	0.889	N37-C40-C44	90.99	C41-N37-C40-H42	117.37
N37-C40	1.417	H42-C40-H43	110.71	C41-N37-C40-H43	-114.56

N37-C41	1.500	H42-C40-C44	113.35	C41-N37-C40-C44	1.2
C40-H42	0.969	H43-C40-C44	113.01	H38-N37-C41-C44	115.43
C40-H43	0.971	N37-C41-C44	84.59	H38-N37-C41-H45	-130
C40-C44	1.454	N37-C41-H45	114.45	H38-N37-C41-H46	1.2
C41-C44	1.543	N37-C41-H46	113.97	H39-N37-C41-C44	-117.49
C41-H45	0.964	C44-C41-H45	114.57	H39-N37-C41-H45	-2.92
C41-H46	0.970	C44-C41-H46	114.32	H39-N37-C41-H46	128.28
C44-H47	0.968	H45-C41-H46	112.31	C40-N37-C41-C44	-1.13
C44-H48	0.972	C40-C44-C41	90.58	C40-N37-C41-H45	113.43
N49-H50	0.887	C40-C44-H47	113.9	C40-N37-C41-H46	-115.37
N49-H51	0.893	C40-C44-H48	113.5	N37-C40-C44-C41	-1.16
N49-C52	1.442	C41-C44-H47	113.42	N37-C40-C44-H47	-117.22
N49-C58	1.607	C41-C44-H48	113.41	N37-C40-C44-H48	114.79
C52-H53	0.968	H47-C44-H48	110.77	H42-C40-C44-C41	-117.75
C52-H54	0.969	H50-N49-H51	111.2	H42-C40-C44-H47	126.2
C52-C55	1.601	H50-N49-C52	114.01	H42-C40-C44-H48	-1.8
C55-H56	0.970	H50-N49-C58	113.97	H43-C40-C44-C41	115.26
C55-H57	0.973	H51-N49-C52	113.6	H43-C40-C44-H47	-0.8
C55-C58	1.417	H51-N49-C58	113.89	H43-C40-C44-H48	-128.79
C58-H59	0.965	C52-N49-C58	88.53	N37-C41-C44-C40	1.1
C58-H60	0.972	N49-C52-H53	113.52	N37-C41-C44-H47	117.58
N49-H50	0.887	N49-C52-H54	113.19	N37-C41-C44-H48	-114.93
N49-H51	0.893	N49-C52-C55	90.55	H45-C41-C44-C40	-113.35
N49-C52	1.442	H53-C52-H54	111.05	H45-C41-C44-H47	3.13
N49-C58	1.607	H53-C52-C55	113.68	H45-C41-C44-H48	130.62
C52-H53	0.968	H54-C52-C55	113.5	H46-C41-C44-C40	115
C52-H54	0.969	C52-C55-H56	113.89	H46-C41-C44-H47	-128.53
C52-C55	1.601	C52-C55-H57	113.73	H46-C41-C44-H48	-1.02
C55-H56	0.970	C52-C55-C58	89.65	H50-N49-C52-H53	129
C55-H57	0.973	H56-C55-H57	110.74	H50-N49-C52-H54	1.22
C55-C58	1.417	H56-C55-C58	113.67	H50-N49-C52-C55	-114.75
C58-H59	0.965	H57-C55-C58	113.78	H51-N49-C52-H53	0.21
C58-H60	0.972	N49-C58-C55	91.25	H51-N49-C52-H54	-127.57
		N49-C58-H59	113.25	H51-N49-C52-C55	116.46
		N49-C58-H60	113	C58-N49-C52-H53	-115.31
		C55-C58-H59	113.72	C58-N49-C52-H54	116.92
		C55-C58-H60	113.45	C58-N49-C52-C55	0.94
		H59-C58-H60	110.93	H50-N49-C58-C55	114.66
**Lattice**	**[Å]**			H50-N49-C58-H59	-1.91
a	10.789			H50-N49-C58-H60	-129.13
b	7.647			H51-N49-C58-C5	-116.31
c	16.549			H51-N49-C58-H59	127.12
**Angles**	**[deg]**			H51-N49-C58-H60	-0.1
α	90.00			C52-N49-C58-C55	-1.06
β	91.49			C52-N49-C58-H59	-117.64
γ	90.00			C52-N49-C58-H60	115.15
				N49-C52-C55-H56	114.81

				N49-C52-C55-H57	-117.03
				N49-C52-C55-C58	-1.07
				H53-C52-C55-H56	-129.09
				H53-C52-C55-H57	-0.93
				H53-C52-C55-C58	115.04
				H54-C52-C55-H56	0.89
				H54-C52-C55-H57	127.27
				H54-C52-C55-C58	-116.77
				C52-C55-C58-N49	0.96
				C52-C55-C58-H59	117.12
				C52-C55-C58-H60	-114.86
				H56-C55-C58-N49	-115.11
				H56-C55-C58-H59	1.05
				H56-C55-C58-H60	129.07
				H57-C55-C58-N49	116.88
				H57-C55-C58-H59	-126.96
				H57-C55-C58-H60	1.06

Table S7. Geometric and lattice parameters of the **ABB** crystal after optimization at the DFT (HSE06-D3/pob_TZVP_rev2) method level.

Bonds	[Å]	Angles	[deg]	Dihedrals	[deg]2
Bi1-Br2	2.698	Br2-Bi1-Br3	95.68	Br2-Bi1-Br6-Bi1	24.99
Bi1-Br3	2.705	Br2-Bi1-Br4	99.26	Br3-Bi1-Br6-Bi1	-175.36
Bi1-Br4	2.775	Br2-Bi1-Br5	88.67	Br4-Bi1-Br6-Bi1	124.27
Bi1-Br5	2.925	Br2-Bi1-Br6	89.82	Br5-Bi1-Br6-Bi1	-63.69
Bi1-Br6	3.056	Br2-Bi1-Br6	176.51	Br6-Bi1-Br6-Bi1	-153.97
Bi1-Br6	3.055	Br3-Bi1-Br4	89.84	Br8-Bi7-Br12-Bi7	-159.61
Br6-Bi1	3.055	Br3-Bi1-Br5	95.47	Br9-Bi7-Br12-Bi7	62.97
Bi1-Br2	2.698	Br3-Bi1-Br6	174.13	Br10-Bi7-Br12-Bi7	-67.03
Bi1-Br3	2.705	Br3-Bi1-Br6	87.69	Br11-Bi7-Br12-Bi7	104.29
Bi1-Br4	2.775	Br4-Bi1-Br5	169.99	Br12-Bi7-Br12-Bi7	16.74
Bi1-Br5	2.925	Br4-Bi1-Br6	87.25	H14-N13-C16-H18	8.59
Bi1-Br6	3.056	Br4-Bi1-Br6	81.62	H14-N13-C16-H19	135.88
Bi1-Br6	3.055	Br5-Bi1-Br6	86.68	H14-N13-C16-C20	-107.82
Br6-Bi1	3.055	Br5-Bi1-Br6	90.1	H15-N13-C16-H18	-120.47
Bi7-Br8	2.688	Br6-Bi1-Br6	86.85	H15-N13-C16-H19	6.82
Bi7-Br9	2.726	Bi1-Br6-Bi1	159.25	H15-N13-C16-C20	123.12
Bi7-Br12	3.007	Br8-Bi7-Br9	94.53	C17-N13-C16-H18	124.48
Bi7-Br10	2.787	Br8-Bi7-Br12	87.46	C17-N13-C16-H19	-108.23
Bi7-Br11	2.929	Br8-Bi7-Br10	92.52	C17-N13-C16-C20	8.07
Bi7-Br12	3.058	Br8-Bi7-Br11	96.05	H14-N13-C17-C20	107.8
Br12-Bi7	3.058	Br8-Bi7-Br12	175.55	H14-N13-C17-H21	-135.47
Bi7-Br8	2.688	Br9-Bi7-Br12	177.29	H14-N13-C17-H22	-7.83
Bi7-Br9	2.726	Br9-Bi7-Br10	93.02	H15-N13-C17-C20	-123.08
Bi7-Br12	3.007	Br9-Bi7-Br11	89.09	H15-N13-C17-H21	-6.35
Bi7-Br10	2.787	Br9-Bi7-Br12	88.14	H15-N13-C17-H22	121.29

Bi7-Br11	2.929	Br12-Bi7-Br10	88.72	C16-N13-C17-C20	-7.81
Br12-Bi7	3.058	Br12-Bi7-Br11	88.88	C16-N13-C17-H21	108.92
N13-H14	0.886	Br12-Bi7-Br12	89.99	C16-N13-C17-H22	-123.44
N13-H15	0.892	Br10-Bi7-Br11	170.99	N13-C16-C20-C17	-7.94
N13-C16	1.451	Br10-Bi7-Br12	83.77	N13-C16-C20-H23	108.19
N13-C17	1.452	Br11-Bi7-Br12	87.55	N13-C16-C20-H24	-123.1
C16-H18	0.967	Bi7-Br12-Bi7	150.81	H18-C16-C20-C17	-124.66
C16-H19	0.971	H14-N13-H15	111.27	H18-C16-C20-H23	-8.52
C16-C20	1.428	H14-N13-C16	113.99	H18-C16-C20-H24	120.19
C17-C20	1.475	H14-N13-C17	114.28	H19-C16-C20-C17	108.53
C17-H21	0.965	H15-N13-C16	113.79	H19-C16-C20-H23	-135.33
C17-H22	0.974	H15-N13-C17	113.56	H19-C16-C20-H24	-6.62
C20-H23	0.966	C16-N13-C17	88.23	N13-C17-C20-C16	7.94
C20-H24	0.974	N13-C16-H18	113.47	N13-C17-C20-H23	-107.77
N13-H14	0.886	N13-C16-H19	113.27	N13-C17-C20-H24	123.11
N13-H15	0.892	N13-C16-C20	92.17	H21-C17-C20-C16	-108.43
N13-C16	1.451	H18-C16-H19	110.68	H21-C17-C20-H23	135.86
N13-C17	1.452	H18-C16-C20	113.12	H21-C17-C20-H24	6.74
C16-H18	0.967	H19-C16-C20	113.07	H22-C17-C20-C16	123.3
C16-H19	0.971	N13-C17-C20	90.29	H22-C17-C20-H23	7.6
C16-C20	1.428	N13-C17-H21	113.77	H22-C17-C20-H24	-121.52
C17-C20	1.475	N13-C17-H22	113.04	H26-C25-C28-H30	-120.14
C17-H21	0.965	C20-C17-H21	114.18	H26-C25-C28-H31	6.87
C17-H22	0.974	C20-C17-H22	113.33	H26-C25-C28-C32	123.25
C20-H23	0.966	H21-C17-H22	110.88	H27-C25-C28-H30	6.92
C20-H24	0.974	C16-C20-C17	88.21	H27-C25-C28-H31	133.94
C25-H26	0.887	C16-C20-H23	114.04	H27-C25-C28-C32	-109.68
C25-H27	0.896	C16-C20-H24	113.7	C29-C25-C28-H30	122.82
C25-C28	1.452	C17-C20-H23	114.51	C29-C25-C28-H31	-110.17
C25-C29	1.468	C17-C20-H24	113.68	C29-C25-C28-C32	6.21
C28-H30	0.970	H23-C20-H24	111.05	H26-C25-C29-C32	-123.22
C28-H31	0.973	H26-C25-H27	110.34	H26-C25-C29-H33	120.85
C28-C32	1.471	H26-C25-C28	113.92	H26-C25-C29-H34	-7.78
C29-C32	1.509	H26-C25-C29	113.79	H27-C25-C29-C32	109.98
C29-H33	0.968	H27-C25-C28	113.11	H27-C25-C29-H33	-5.95
C29-H34	0.974	H27-C25-C29	112.94	H27-C25-C29-H34	-134.58
C32-H35	0.969	C28-C25-C29	91.7	C28-C25-C29-C32	-6.06
C32-H36	0.970	C25-C28-H30	113.62	C28-C25-C29-H33	-121.99
C25-H26	0.887	C25-C28-H31	113.04	C28-C25-C29-H34	109.38
C25-H27	0.896	C25-C28-C32	90.16	C25-C28-C32-C29	-6.04
C25-C28	1.452	H30-C28-H31	110.53	C25-C28-C32-H35	109.48
C25-C29	1.468	H30-C28-C32	114.15	C25-C28-C32-H36	-121.66
C28-H30	0.970	H31-C28-C32	114.07	H30-C28-C32-C29	-122.17
C28-H31	0.973	C25-C29-C32	88.12	H30-C28-C32-H35	-6.65
C28-C32	1.471	C25-C29-H33	114.26	H30-C28-C32-H36	122.21
C29-C32	1.509	C25-C29-H34	113.47	H31-C28-C32-C29	109.42
C29-H33	0.968	C32-C29-H33	114.32	H31-C28-C32-H35	-135.06

C29-H34	0.974	C32-C29-H34	114.01	H31-C28-C32-H36	-6.2
C32-H35	0.969	H33-C29-H34	111	C25-C29-C32-C28	5.98
C32-H36	0.970	C28-C32-C29	89.37	C25-C29-C32-H35	-110.07
N37-H38	0.889	C28-C32-H35	114.03	C25-C29-C32-H36	121.91
N37-H39	0.891	C28-C32-H36	113.9	H33-C29-C32-C28	121.86
N37-C40	1.429	C29-C32-H35	113.45	H33-C29-C32-H35	5.81
N37-C41	1.475	C29-C32-H36	113.56	H33-C29-C32-H36	-122.21
C40-H42	0.967	H35-C32-H36	111.03	H34-C29-C32-C28	-108.95
C40-H43	0.972	H38-N37-H39	110.76	H34-C29-C32-H35	135
C40-C44	1.456	H38-N37-C40	113.75	H34-C29-C32-H36	6.98
C41-C44	1.481	H38-N37-C41	113.27	H38-N37-C40-H42	119.54
C41-H45	0.972	H39-N37-C40	113.39	H38-N37-C40-H43	-8.61
C41-H46	0.973	H39-N37-C41	113.04	H38-N37-C40-C44	-124.2
C44-H47	0.965	C40-N37-C41	91.46	H39-N37-C40-H42	-8.21
C44-H48	0.969	N37-C40-H42	113.86	H39-N37-C40-H43	-136.36
N37-H38	0.889	N37-C40-H43	113.35	H39-N37-C40-C44	108.05
N37-H39	0.891	N37-C40-C44	90.01	C41-N37-C40-H42	-124.16
N37-C40	1.429	H42-C40-H43	111	C41-N37-C40-H43	107.69
N37-C41	1.475	H42-C40-C44	113.86	C41-N37-C40-C44	-7.9
C40-H42	0.967	H43-C40-C44	113.36	H38-N37-C41-C44	124.5
C40-H43	0.972	N37-C41-C44	87.31	H38-N37-C41-H45	9.06
C40-C44	1.456	N37-C41-H45	114.23	H38-N37-C41-H46	-120.1
C41-C44	1.481	N37-C41-H46	114.24	H39-N37-C41-C44	-108.48
C41-H45	0.972	C44-C41-H45	114.24	H39-N37-C41-H45	136.09
C41-H46	0.973	C44-C41-H46	114.21	H39-N37-C41-H46	6.93
C44-H47	0.965	H45-C41-H46	110.9	C40-N37-C41-C44	7.78
C44-H48	0.969	C40-C44-C41	90.16	C40-N37-C41-H45	-107.66
N49-H50	0.885	C40-C44-H47	113.81	C40-N37-C41-H46	123.18
N49-H51	0.887	C40-C44-H48	113.32	N37-C40-C44-C41	7.87
N49-C52	1.469	C41-C44-H47	113.51	N37-C40-C44-H47	123.79
N49-C53	1.533	C41-C44-H48	113.28	N37-C40-C44-H48	-107.69
C52-H54	0.967	H47-C44-H48	111.31	H42-C40-C44-C41	124.12
C52-H55	0.968	H50-N49-H51	111.95	H42-C40-C44-H47	-119.96
C52-C56	1.432	H50-N49-C52	113.85	H42-C40-C44-H48	8.56
C53-C56	1.436	H50-N49-C53	114.11	H43-C40-C44-C41	-107.72
C53-H57	0.968	H51-N49-C52	113.55	H43-C40-C44-H47	8.2
C53-H58	0.972	H51-N49-C53	114.03	H43-C40-C44-H48	136.72
C56-H59	0.968	C52-N49-C53	87.28	N37-C41-C44-C40	-7.63
C56-H60	0.974	N49-C52-H54	114.15	N37-C41-C44-H47	-123.82
N49-H50	0.885	N49-C52-H55	113.94	N37-C41-C44-H48	107.97
N49-H51	0.887	N49-C52-C56	88.88	H45-C41-C44-C40	107.79
N49-C52	1.469	H54-C52-H55	111.47	H45-C41-C44-H47	-8.4
N49-C53	1.533	H54-C52-C56	113.52	H45-C41-C44-H48	-136.61
C52-H54	0.967	H55-C52-C56	113.19	H46-C41-C44-C40	-123.06
C52-H55	0.968	N49-C53-C56	86.28	H46-C41-C44-H47	120.74
C52-C56	1.432	N49-C53-H57	114.16	H46-C41-C44-H48	-7.46
C53-C56	1.436	N49-C53-H58	114.26	H50-N49-C52-H54	145.94

C53-H57	0.968	C56-C53-H57	114.17	H50-N49-C52-H55	16.3
C53-H58	0.970	C56-C53-H58	114.24	H50-N49-C52-C56	-98.68
C56-H59	0.968	H57-C53-H58	111.62	H51-N49-C52-H54	16.25
C56-H60	0.974	C52-C56-C53	92.5	H51-N49-C52-H55	-113.39
		C52-C56-H59	113.55	H51-N49-C52-C56	131.63
		C52-C56-H60	113.24	C53-N49-C52-H54	-98.84
		C53-C56-H59	113.25	C53-N49-C52-H55	131.52
		C53-C56-H60	112.99	C53-N49-C52-C56	16.54
		H59-C56-H60	110.35	H50-N49-C53-C56	98.45
**Lattice**	**[Å]**			H50-N49-C53-H57	-16.42
a	10.895			H50-N49-C53-H58	-146.59
b	8.246			H51-N49-C53-C56	-131.16
c	16.747			H51-N49-C53-H57	113.97
**Angles**	**[deg]**			H51-N49-C53-H58	-16.2
α	90.00			C52-N49-C53-C56	-16.53
β	92.87			C52-N49-C53-H57	-131.4
γ	90.00			C52-N49-C53-H58	98.43
				N49-C52-C56-C53	-17.69
				N49-C52-C56-H59	99.06
				N49-C52-C56-H60	-134.06
				H54-C52-C56-C53	98.28
				H54-C52-C56-H59	-144.97
				H54-C52-C56-H60	-18.09
				H55-C52-C56-C53	-133.35
				H55-C52-C56-H59	-16.6
				H55-C52-C56-H60	110.28
				N49-C53-C56-C52	16.96
				N49-C53-C56-H59	-100.05
				N49-C53-C56-H60	133.55
				H57-C53-C56-C52	131.81
				H57-C53-C56-H59	14.81
				H57-C53-C56-H60	-111.6
				H58-C53-C56-C52	-98.02
				H58-C53-C56-H59	144.97
				H58-C53-C56-H60	18.57

UV-vis spectroscopy

The UV-vis diffuse reflectance spectra of a polycrystalline **ABB** and **ABC** were recorded at room temperature in the spectral range 250-800 nm, with a Cary-5000 UV–Vis-NIR spectrophotometer. The UV-vis luminescence spectra were measured at 77 K on an Edinburgh Instruments FLS 920 spectrometer. The measurements were performed in a nitrogen cryostat Optistat DN (Oxford Instruments).

Second harmonic generation (SHG) studies

Nonlinear optical experiments were performed using a laser system employing a wavelength-tunable Topaz Prime Vis-NIR optical parametric amplifier (OPA) pumped by Coherent Astrella Ti:Sapphire regenerative amplifier providing femtosecond laser pulses (800 nm, 75 fs) at 1 kHz repetition rate. The output of OPA was set to 1400 nm and the laser fluence at samples was equal 0.19 mJ/cm^2. The single crystals of **ABC**, **ABB** and KDP were crushed with a spatula and sieved through an Aldrich mini-sieve set, collecting a microcrystal size fraction of 88–125 µm. Next, size-graded samples were fixed in-between microscope glass slides to form tightly packed layers, sealed, and mounted to the horizontally aligned sample holder. No refractive index matching oil was used. The employed measurement setup operates in the reflection mode. Specifically, the laser beam was directed onto the sample at 45 degrees to its surface. Emission collecting optics consisted of a Ø25.0 mm plano-convex lens of focal length 25.4 mm mounted to the 400 µm 0.22 NA glass optical fiber and was placed along the normal to the sample surface. The distance between collection lens and the sample was equal to 30 mm. The spectra of the temperature-dependent SHG responses were recorded by an Ocean Optics Flame T XR fiber-coupled CCD spectrograph with 200 µm entrance slit. Scattered pumping radiation was suppressed with the use of a Thorlabs 750 nm hard-coated short-pass dielectric filter. The temperature control of the sample was performed using a Linkam LTS420 Heating/Freezing Stage. Temperature stability was equal to 0.1 K. TR-SHG study of **ABC** and **ABB** was conducted in a range of 123 – 293 K.

Kurtz-Perry powder test was performed by comparing the SHG signals of **ABC** and **ABB** collected at 123 K with that of the KDP standard, after normalizing SHG spectra to the same integration time.

The SHG switching experiment employed **ABC** and **ABB** samples prepared in the same way as well as the same optical setup and beam parameters as for TR-SHG and Kurtz-Perry studies. It was performed by alternating the samples' temperature with 5, 10, 20, 35, 50 K/min rates, with each cycle followed by the isothermal time period of 30s. Boundary temperatures corresponding to SHG-*on* and SHG-*off* states, employed for switching experiments, are provided in Figure S17, Figure S18 and Figure 11b.

a)

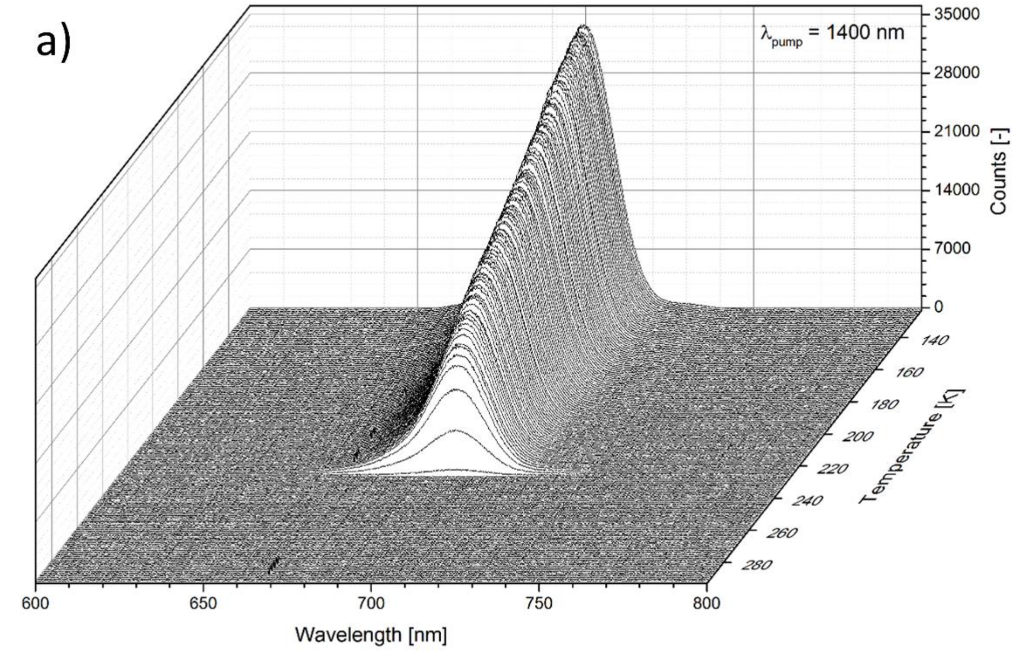

b)

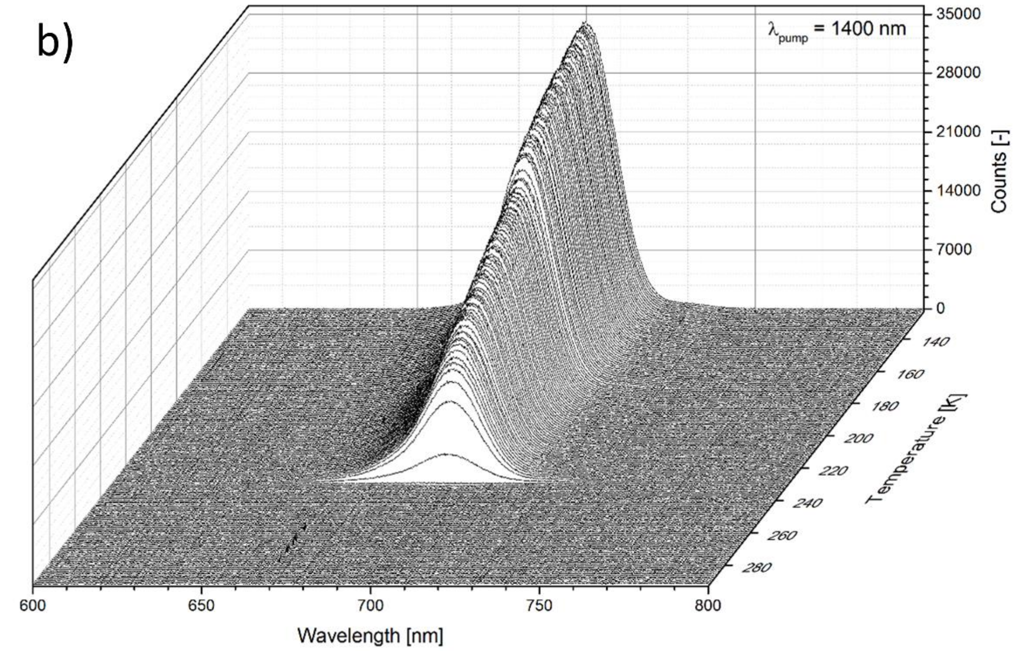

Figure S14. Overlay of experimental SHG spectra obtained upon irradiation with 1400 nm femtosecond laser pulses of **ABC** for (a) cooling in 293 K - 123 K range, (b) heating in 123 K - 293 K range.

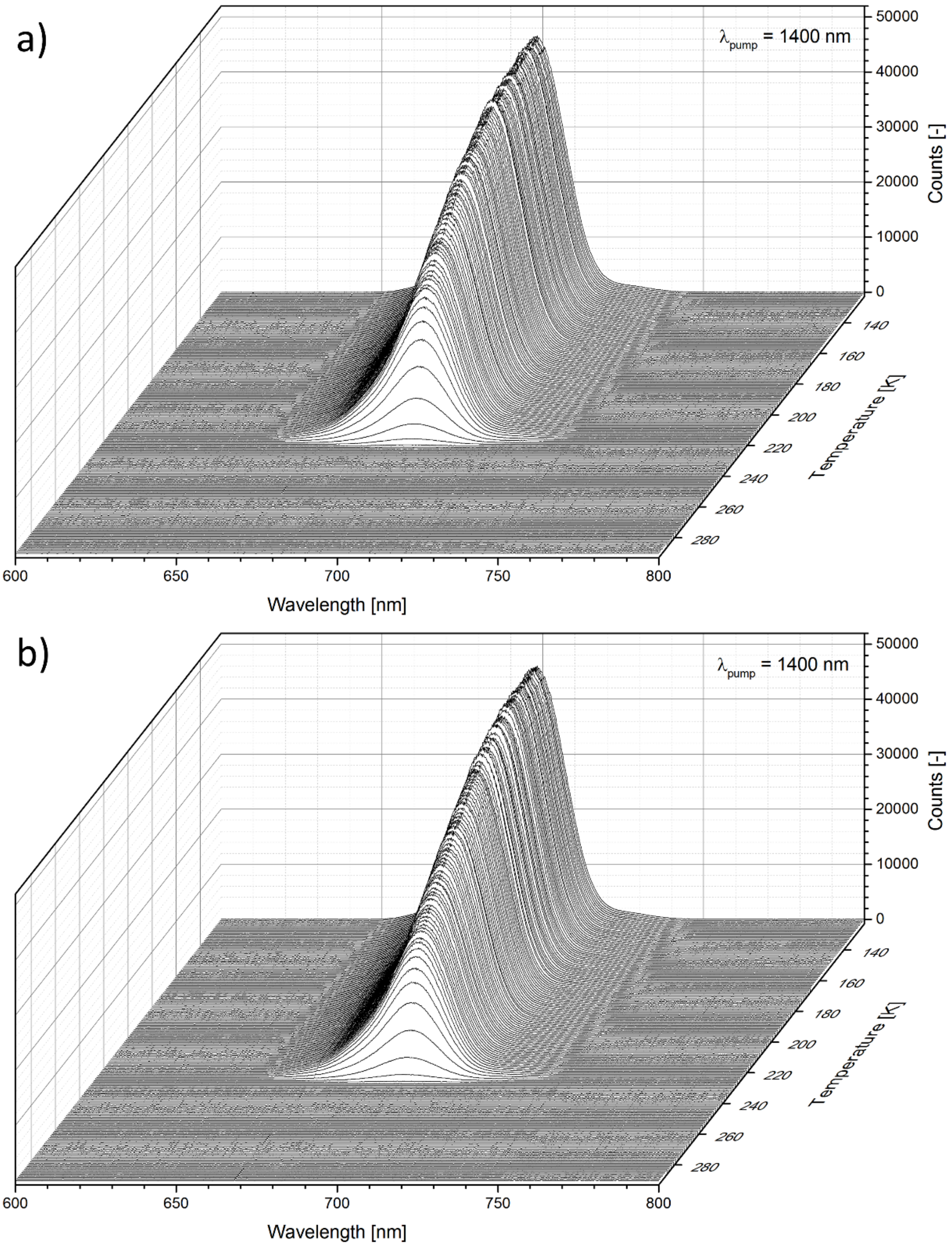

Figure S15. Overlay of experimental SHG spectra obtained upon irradiation with 1400 nm femtosecond laser pulses of **ABB** for (a) cooling in 293 K - 123 K range, (b) heating in 123 K - 293 K range.

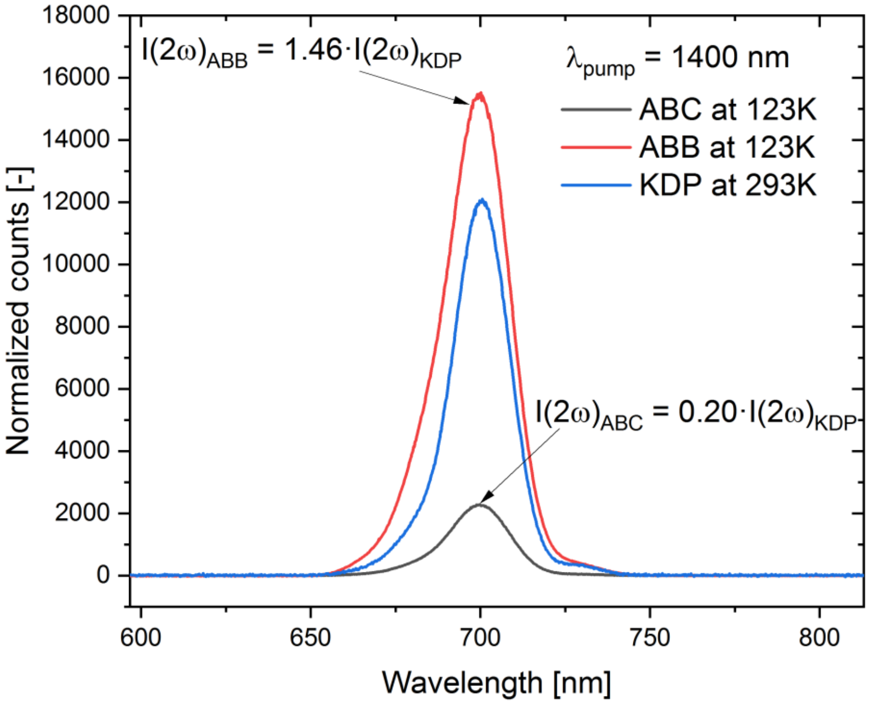

Figure S16. Overlay of SHG traces of **ABC** and **ABB** with that of KDP obtained upon irradiation with 1400 nm femtosecond laser pulses. SHG traces are normalized to the same integration time.

ABC

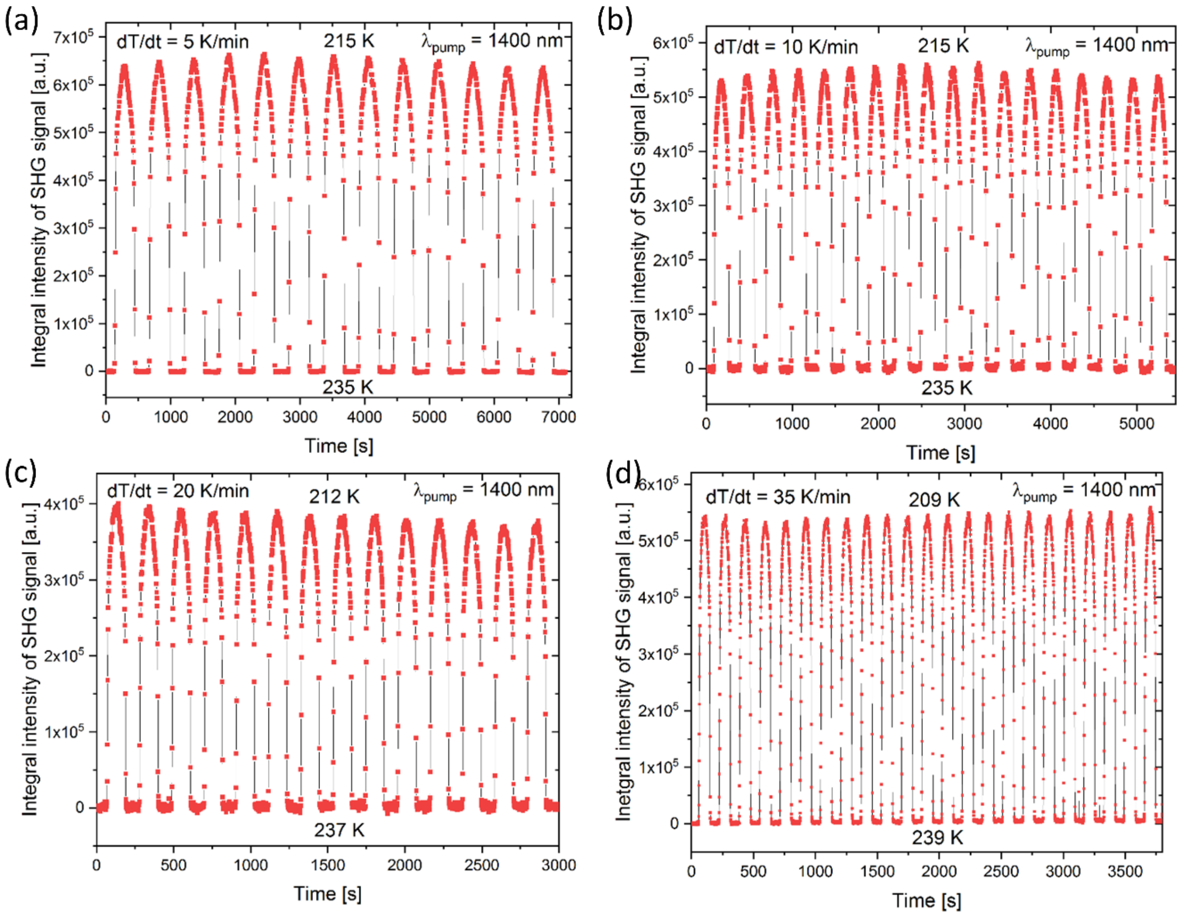

Figure S17. Plots of integral intensities of SHG signals (λ_{SHG} = 700 nm) obtained during temperature-induced switching experiment for **ABC** at a) 5 K/min, b) 10 K/min, c) 20 K/min, d) 35 K/min heating-cooling rate.

ABB

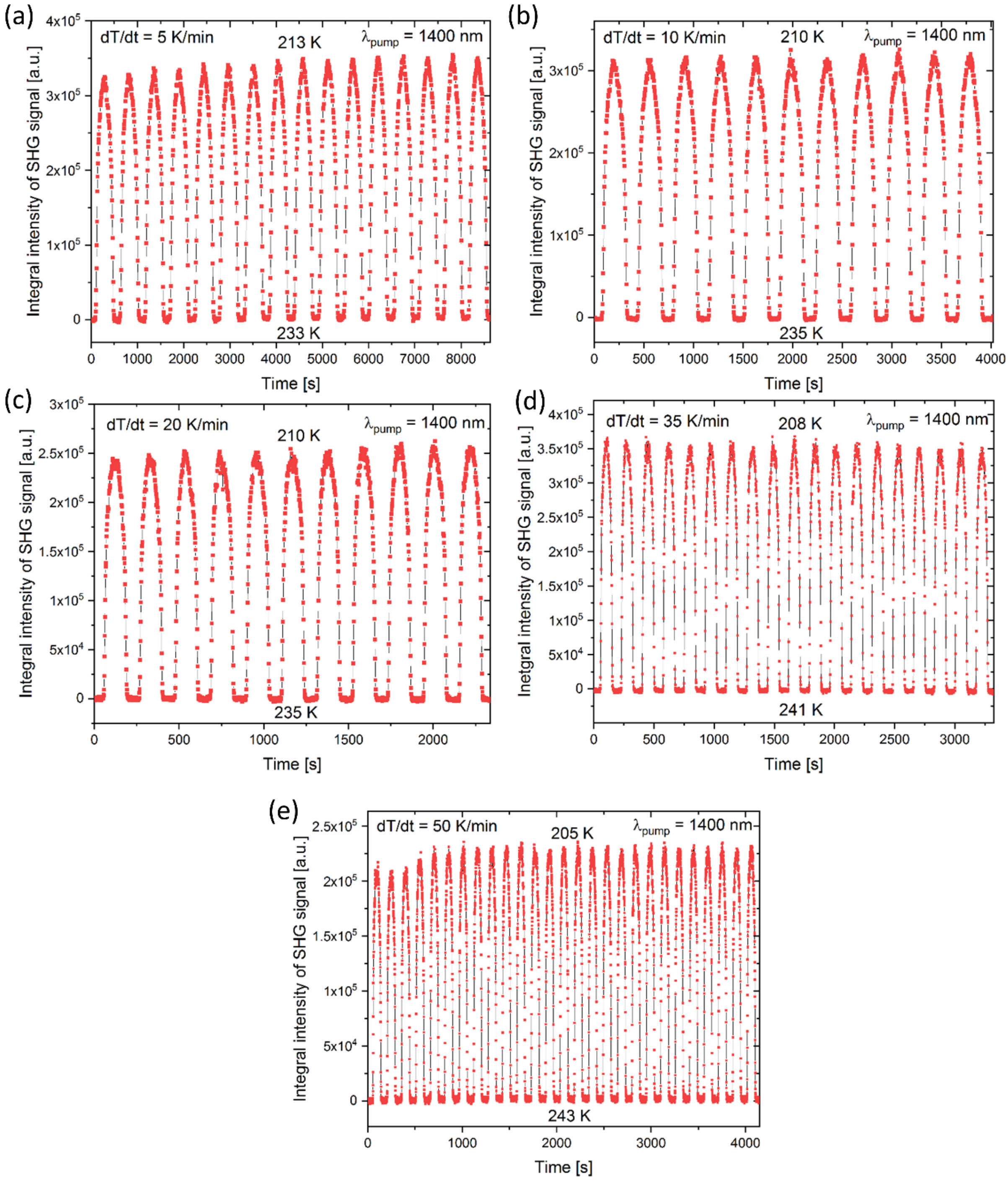

Figure S18. Plots of integral intensities of SHG signals (λ_{SHG} = 700 nm) obtained during temperature-induced switching experiment for **ABB** at a) 5 K/min, b) 10 K/min, c) 20 K/min, d) 35 K/min, e) 50 K/min heating-cooling rate.

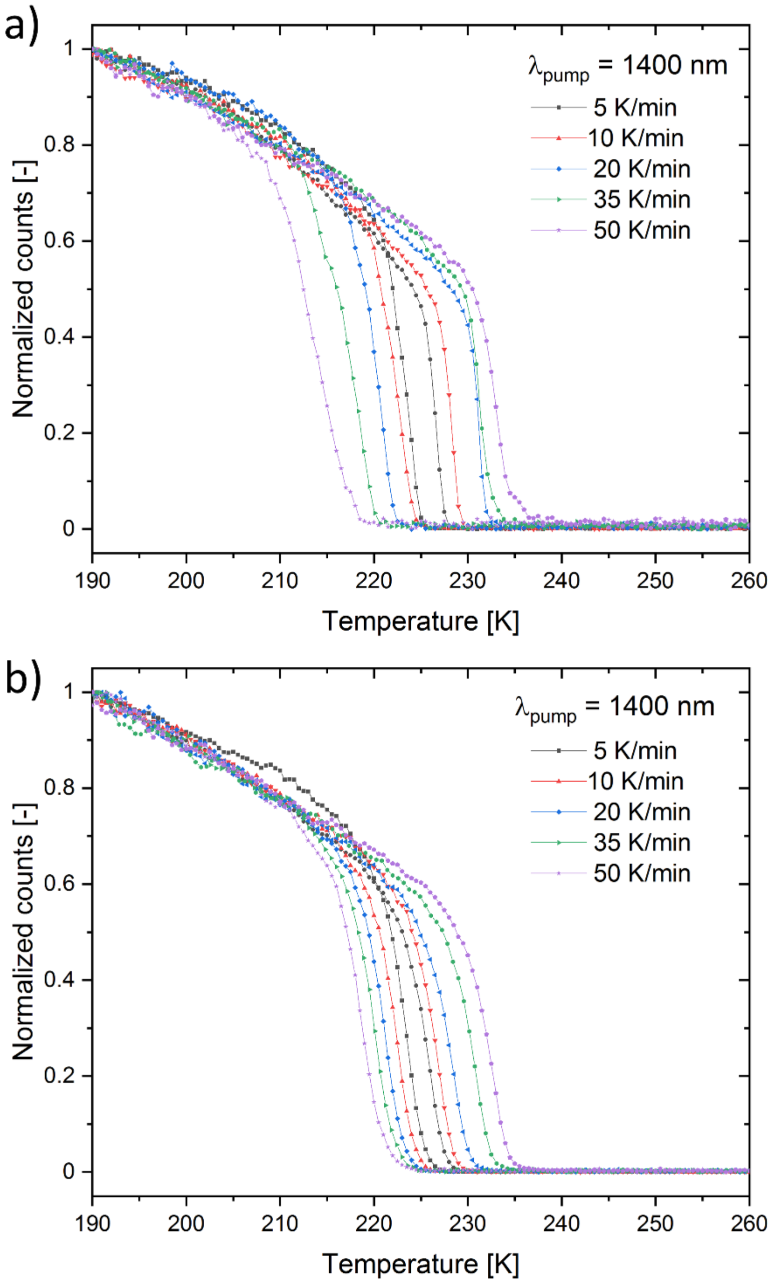

Figure S19. SHG temperature hystereses registered for different temperature change rates (5, 10, 20, 35, and 50 K/min) for a) **ABC** and b) **ABB**.

Neutron Spectroscopy

High-resolution neutron spectra were measured on the backscattering spectrometer SPHERES[34,35] operated by JCNS at the Heinz Maier-Leibnitz Zentrum (Garching, Germany) at temperatures between 3 and 360 K in the energy range of ±30 μeV. The experiment employs a neutron wavelength of 6.27 Å (E = 2.08 meV) with energy resolution (full width at half-maximum, fwhm) of~0.66 μeV for large scattering angles. The Q-range investigated varied from 0.2 to 1.8 Å. The programs SLAW[36] and FRIDA[35] were used for reducing and fitting the data. The powder samples were obtained by milling the crystals for 1 min in an agate mortar. The instrument resolution function was determined by measuring the samples at ~3K, where only elastic scattering was observed.

Solid state NMR

The spin-lattice relaxation time T_1 was measured using an ELLAB TEL-Atomic PS 15 spectrometer working at 25 MHz from 84 K to 290 K. The T_1 relaxation times were determined using a π - τ - $\pi/2$ method. Errors in the measured T_1 values were estimated to be lower than 5%. The measurements of the proton NMR line second moment were carried out with a wide-line ELLAB TEL-Atomic CW Spectrometer operating at 26.8 MHz from 116 K to 290 K. The second moment (M_2) values were calculated by numeral integration of the first derivative of an absorption line and corrected for the finite modulation amplitude. The temperature of the sample was automatically controlled by a UNIPAN 660 temperature unit with a Pt-100 sensor. The powdered sample of **ABB** was briefly degassed under pressure of 10^{-5} Torr and sealed in glass ampoules.

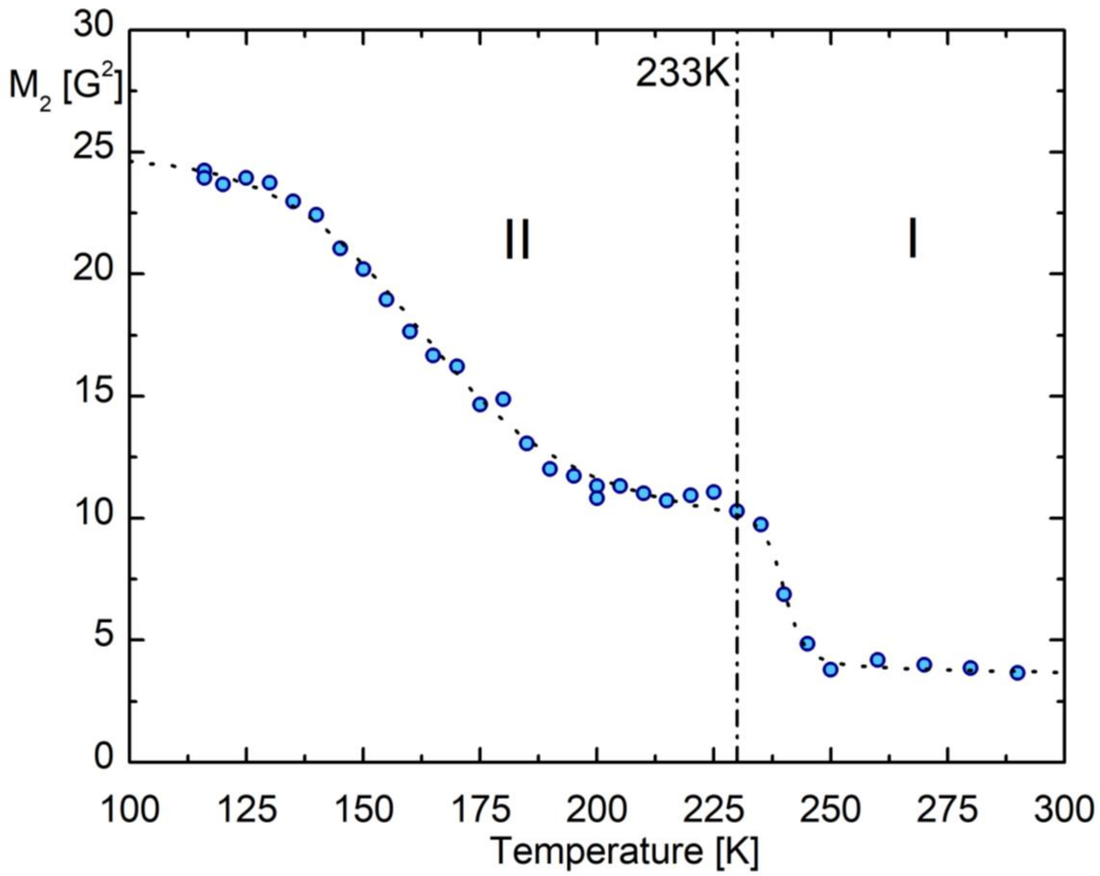

Figure S20. Temperature dependence of the second moment of ^{1}HNMR line of **ABB**

References

(1) *CrysAlis RED, CrysAlis CCD,Oxford Diffraction:*; Oxford Diffraction Ltd, Abingdon, England, 2008.

(2) Sheldrick, G. M. Crystal Structure Refinement with SHELXL. *Acta Crystallogr. Sect. C Struct. Chem.* **2015**, *71* (Md), 3–8.

(3) MacRae, C. F.; Sovago, I.; Cottrell, S. J.; Galek, P. T. A.; McCabe, P.; Pidcock, E.; Platings, M.; Shields, G. P.; Stevens, J. S.; Towler, M.; et al. Mercury 4.0: From Visualization to Analysis, Design and Prediction. *J. Appl. Crystallogr.* **2020**, *53*, 226–235.

(4) Cole, K. S.; Cole, R. H. Dispersion and Absorption in Dielectrics I. Alternating Current Characteristics. *J. Chem. Phys.* **1941**, *9*, 341–351.

(5) Li, P.; Tang, Y.; Liao, W.; Ye, H.; Zhang, Y.; Fu, D.; You, Y. A Semiconducting Molecular Ferroelectric with a Bandgap Much Lower than That of BiFeO3. *NPG Asia Mater.* **2017**, *9*, 1–6.

(6) Ksiądzyna, M.; Gągor, A.; Piecha-Bisiorek, A.; Ciżman, A.; Medycki, W.; Jakubas, R. Exploring a Hybrid Ferroelectric with a 1-D Perovskite-like Structure: Bis(Pyrrolidinium) Pentachloroantimonate(III). *J. Mater. Chem. C* **2019**, *7* (33), 10360–10370.

(7) Bi, W.; Leblanc, N.; Mercier, N.; Auban-Senzier, P.; Pasquier, C. Thermally Induced Bi(III) Lone Pair Stereoactivity: Ferroelectric Phase Transition and Semiconducting Properties of (MV)BiBr5 (MV = Methylviologen). *Chem. Mater.* **2009**, *21* (18), 4099–4101.

(8) Leblanc, N.; Mercier, N.; Zorina, L.; Simonov, S.; Auban-Senzier, P.; Pasquier, C. Large Spontaneous Polarization and Clear Hysteresis Loop of a Room-Temperature Hybrid Ferroelectric Based on Mixed-Halide [BiI 3Cl2] Polar Chains and Methylviologen Dication. *J. Am. Chem. Soc.* **2011**, *133* (38), 14924–14927.

(9) Piecha-Bisiorek, A.; Gągor, A.; Jakubas, R.; Ciżman, A.; Janicki, R.; Medycki, W. Ferroelectricity in Bis(Ethylammonium) Pentachlorobismuthate(III): Synthesis, Structure, Polar and Spectroscopic Properties. *Inorg. Chem. Front.* **2017**, *4*, 1281–1286.

(10) Jakubas, R.; Gągor, A.; Winiarski, M. J.; Ptak, M.; Piecha-Bisiorek, A.; Ciżman, A. Ferroelectricity in Ethylammonium Bismuth-Based Organic-Inorganic Hybrid: (C2H5NH3)2[BiBr5]. *Inorg. Chem.* **2020**, *59*, 3417−3427.

(11) Piecha, A.; Białońska, A.; Jakubas, R. Novel Organic-Inorganic Hybrid Ferroelectric: Bis(Imidazolium) Pentachloroantimonate(Iii), (C 3N 2H 5) 2SbCl 5. *J. Mater. Chem.* **2012**, *22* (2), 333–336.

(12) Zhao, W. P.; Shi, C.; Stroppa, A.; Di Sante, D.; Cimpoesu, F.; Zhang, W. Lone-Pair-Electron-Driven Ionic Displacements in a Ferroelectric Metal-Organic Hybrid. *Inorg. Chem.* **2016**, *55* (20), 10337–10342.

(13) Wang, Y.; Shi, C.; Han, X. Bin. Organic–Inorganic Hybrid [H2mdap][BiCl5] Showing an above-Room-Temperature Ferroelectric Transition with Combined Order–Disorder and Displacive Origins. *Polyhedron* **2017**, *133*, 132–136.

(14) Chen, Q.; Jiang, H.; Fan, Y.; Li, Z.; Ye, H.; Yao, Y.; Chen, S.; Ji, C.; Zhang, S.; Luo, J. High-Tc Realization of Lead-Free Halide Hybrid Ferroelectrics via Steric Confinement Modulation. *Adv. Funct. Mater.* **2023**, *33*, 1–7.

(15) Song, N.; Dong, X. X.; Zhuang, J. C.; Li, Y. K.; Han, D. C.; Tan, Y. H.; Wei, W. J.; Tang, Y. Z. Coupling Narrow Band Gap and Switchable SHG Responses in a New Molecule Ferroelectric: Imidazolyl Propylamine Pentabromo Stibium(III). *Inorg. Chem.* **2021**, *60*, 1195–1201.

(16) Liu, Y. H.; Peng, H.; Liao, W. Q. A Lead-Free Bismuth Iodide Organic-Inorganic Ferroelectric Semiconductor. *Chem. Commun.* **2021**, *57*, 647–650.

(17) Zhang, W.; Hong, M.; Luo, J. Centimeter-Sized Single Crystal of a One-Dimensional Lead-Free Mixed-Cation Perovskite Ferroelectric for Highly Polarization Sensitive Photodetection. *J. Am. Chem. Soc.* **2021**, *143*, 16758–16767.

(18) Zhang, G.; Zhu, M.; Guan, J.; Liu, X.; Zeng, T.; Yang, W. Polarization-Enhanced Photovoltaic Effects in a High-Temperature Molecular Ferroelectric [C6N2H18][SbI5]-Based Solar Device. *ACS Appl. Energy Mater.* **2022**, *5*, 2738–2746.

(19) Dovesi, R.; Erba, A.; Orlando, R.; Zicovich-Wilson, C. M.; Civalleri, B.; Maschio, L.; Rérat, M.; Casassa, S.; Baima, J.; Salustro, S.; et al. Quantum-Mechanical Condensed Matter Simulations with CRYSTAL. *Wiley Interdiscip. Rev. Comput. Mol. Sci.* **2018**, *8* (4), 1–36.

(20) Dovesi, R.; Saunders, V. R.; Roetti, C.; Orlando, R.; Zicovich-Wilson, C. M.; Pascale, F.; Civalleri, B.; Doll, K.; Harrison, N. M.; Bush, I. J.; et al. *CRYSTAL17 User's Manual*; University of Torino: Torino, 2017.

(21) Grimme, S. Semiempirical GGA-Type Density Functional Constructed with a Long-Range Dispersion Correction STEFAN. *J. Comput. Chem.* **2006**, *27*, 1787–1799.

(22) Grimme, S.; Antony, J.; Ehrlich, S.; Krieg, H. A Consistent and Accurate Ab Initio Parametrization of Density Functional Dispersion Correction (DFT-D) for the 94 Elements H-Pu. *J. Chem. Phys.* **2010**, *132* (15).

(23) Grimme, S.; Ehrlich, S.; Goerigk, L. Effect of the Damping Function in Dispersion Corrected Density Functional Theory. *J. Comput. Chem.* **2011**, *32* (7), 1456–1465.

(24) Grimme, S.; Hansen, A.; Brandenburg, J. G.; Bannwarth, C. Dispersion-Corrected Mean-Field Electronic Structure Methods. *Chem. Rev.* **2016**, *116* (9), 5105–5154.

(25) Krukau, A. V.; Vydrov, O. A.; Izmaylov, A. F.; Scuseria, G. E. Influence of the Exchange Screening Parameter on the Performance of Screened Hybrid Functionals. *J. Chem. Phys.* **2006**, *125* (22).

(26) Perdew, J.; Burke, K.; Ernzerhof, M. Generalized Gradient Approximation Made Simple. *Phys. Rev. Lett.* **1996**, *77* (18), 3865–3868.

(27) Monkhorst, H. J.; Pack, J. D. Special Points for Brillouin-Zone Integrations. *Phys. Rev. B* **1976**, *13*, 5188–5192.

(28) Vilela Oliveira, D.; Laun, J.; Peintinger, M. F.; Bredow, T. BSSE-Correction Scheme for Consistent Gaussian Basis Sets of Double- and Triple-Zeta Valence with Polarization Quality for Solid-State Calculations. *J. Comput. Chem.* **2019**, *40* (27), 2364–2376.

(29) Laun, J.; Vilela Oliveira, D.; Bredow, T. Consistent Gaussian Basis Sets of Double- and Triple-Zeta Valence with Polarization Quality of the Fifth Period for Solid-State Calculations. *J. Comput. Chem.* **2018**, *39* (19), 1285–1290.

(30) Laun, J.; Bredow, T. BSSE-Corrected Consistent Gaussian Basis Sets of Triple-Zeta Valence with Polarization Quality of the Sixth Period for Solid-State Calculations. *J. Comput. Chem.* **2021**, *42* (15), 1064–1072.

(31) Laun, J.; Bredow, T. BSSE-Corrected Consistent Gaussian Basis Sets of Triple-Zeta Valence with Polarization Quality of the Fifth Period for Solid-State Calculations. *J. Comput. Chem.* **2022**, *43* (12), 839–846.

(32) Hinuma, Y.; Pizzi, G.; Kumagai, Y.; Oba, F.; Tanaka, I. Band Structure Diagram Paths Based on Crystallography. *Comput. Mater. Sci.* **2017**, *128*, 140–184.

(33) Williams, T.; Kelley, C. Gnuplot 5.0. 2017, pp 1–259.

(34) Wuttke, J.; Zamponi, M. Simulation-Guided Optimization of Small-Angle Analyzer Geometry in the Neutron Backscattering Spectrometer SPHERES. *Rev. Sci. Instrum.* **2013**, *84* (11), 115108.

(35) Wuttke, J.; Budwig, A.; Drochner, M.; Kämmerling, H.; Kayser, F. J.; Kleines, H.; Ossovyi, V.; Pardo, L. C.; Prager, M.; Richter, D.; et al. SPHERES, Jülichs High-Flux Neutron Backscattering Spectrometer at FRM II. *Revi. Sci. Instrum.* **2012**, *83*, 075109-1–11.

(36) Wuttke, J. SLAW--- a neutron histogram to scattering law converter, http://apps.jcns.fz-juelich.de/slaw.

(37) Wuttke, J. FRIDA --- Flexible rapid interactive data analysis, http://apps.jcns.fz-juelich.de/frida.
