# Supplementary material for: Ferroelectric, Switchable Dielectric and Nonlinear Optical Properties in Inorganic–Organic Lead-Free 1D Hybrids Based on Bi(III) and Azetidine: (C3NH8)2[BiCl5], (C3NH8)2[BiBr5]
Source: J Phys Chem Lett. 2024 Nov 15;15(47):11709–22. doi: 10.1021/acs.jpclett.4c02695 (PMC11613664; doi:10.1021/acs.jpclett.4c02695)
Supplement: Supplementary file 4 — jz4c02695_si_006.pdf [file jz4c02695_si_006.pdf]

jz-2024-02695r.R1

Name: Peer Review Information for "Ferroelectric, Switchable Dielectric and Nonlinear Optical Properties in Inorganic-Organic Lead-Free 1D Hybrids Based on Bi(III) and Azetidine: $(C_3NH_8)_2[BiCl_5]$, $(C_3NH_8)_2[BiBr_5]$"

First Round of Reviewer Comments

Reviewer: 1

Comments to the Author

This work demonstrates a comprehensive investigation of the phase transition behavior of two new halobismuthates(III) hybrid crystals, coupling with dielectric, ferroelectric, and optical properties. The experimental analysis and conclusions are convincing for appealing to the attention of the related fields. I recommend it for publication but the current version should be revised before the final acceptance.

1. The authors mentioned many structural analogs with phase transition and ferroelectricity have been reported. While I was wondering about the core difference between the current using Azetidine and others. Is there significant new insight for the phase transition mechanism, enhancing physical performance, or something else? Or just using a new amine for new hybrid crystals for ferroelectrics?

2. The cis and trans-connected MX6 octahedra in various structural frameworks were highlighted in the portion of the introduction. The definition should be provided for figuring out the classification. Besides, the authors mentioned trans types are rare and exhibit high performance. However, the title hybrids in this work belong to the commonly reported cis type. The introduction section must be properly revised to highlight the purpose of the current work for comparison with others.

Reviewer: 2

Comments to the Author

Exploring lead-free organic-inorganic hybrid ferroelectrics is important for developing environment-friendly optoelectronic devices. In this manuscript, the authors reported two new lead-free organic-inorganic ferroelectrics $(C_3NH_8)_2[BiCl_5]$ and $(C_3NH_8)_2[BiBr_5]$. They exhibit ferroelectricity

accompanied by dielectric and second harmonic generation switching behaviors, which have been systematically characterized. I think this work is interesting to the scientific communities of organic-inorganic hybrids and molecular ferroelectrics. Therefore, it is recommended for publication in The Journal of Physical Chemistry Letters after some minor revision.

1. The authors stated that "Both compounds exhibit paraelectric (I) to ferroelectric (II) phase transitions (PTs) at 230/233K and 228/229K, respectively, transitioning from orthorhombic (Pnma) to monoclinic (P21) phases". The phase transition from Pnma (high-temperature phase) to P21 (low-temperature phase) does not belong to the 88 species of paraelectric-ferroelectric phase transitions given by Aizu. Please carefully check the crystal symmetry and give a corresponding explanation.

2. In Figure 3, the two phases (Ⅱ phase and Ⅲ phase) of overlayed organic and Bi chains are difficult to distinguish.

3. The crystal growth process is too brief. More details are needed for others to reproduce the synthesis and crystal growth.

4. In the main text, there is no relevant description of Figures S2 and S4.

5. Some level B Alerts appear in the checkcif. The authors should try to eliminate these alerts or give a response.

Reviewer: 3

Comments to the Author

The article presented to me for review presents new and very interesting research results on functional organic-inorganic hybrid materials. I believe that the article will be suitable for publication because of the very minor corrections listed below:

1. In Figure 6a, the electric field strength should be presented in kV/cm units and not V as it is currently. Although the P(E) dependence itself could not be classified as a ferroelectric loop, the inclusion of the I(V) dependence fully represents polarization switching in an electrically lossy material, especially since it is supported by Figure 9S. Although on page 15, line 19, it is written about the influence of AC conductivity, it seems that at a measuring field frequency of 0.5 Hz, it should rather be about the influence of DC conductivity (similarly on page 16, line 40)

2. In the case of Figure 2S, in my opinion, the presentation of measured and calculated diffraction patterns should be done in such a way that they can be easily compared. Although the authors have attached a graph of the difference between these two diffraction patterns, I believe that they should be presented one for the other and not on oppositely directed intensity axes.

3. On page 16, line 52, the authors of the work somewhat exaggeratedly attributed a change in the sign of the pyroelectric current with a change in the sign of the polarizing field. This effect is known in many organic materials and may be related, among others, to charge injection from electrodes and the so-called electret effect. In fact, the only indisputable evidence for the existence of ferroelectric properties is the observation of a ferroelectric hysteresis loop – well-formed and saturated. I recommend the work of M. Maglione Appl. Phys. Lett. 93, 032902 (2008). I think that this radical statement about proving the occurrence of ferroelectricity in the ABB material should be softened. A similar softening will be useful in the final conclusions on page 32, line 10.

4. On page S14, there is no information about the rate of temperature change during dielectric measurements and the intensity of the measuring field, and information whether the ferroelectric loops were obtained using a triangular or sinusoidal measuring field.

5. on page S15 the statement that a step change in permittivity is characteristic of order-disorder transitions is a bit of an exaggeration – in materials such as BaTiO3 or PbZrO3 phase transitions show a step change in permittivity in Tc, but these are not transitions considered to be order-disorder transitions.

After making the minor corrections mentioned and softening some of the statements, I fully recommend the article for publication in the Journal of Chemical Physics Letters.

Author's Response to Peer Review Comments:

Thursday, 10 October 2024
Manuscript ID: jz-2024-02695r

Corresponding Author: dr hab. Magdalena Rok

Dear Editor,

We are grateful to all the Reviewers for their effort in carefully and critically reading the manuscript submitted to JPCL and associated ESI†. The comments and the remarks certainly helped us to make our contribution more clear and more scientifically sound.

The manuscript has been revised carefully according to the Reviewers' comments and improve. The point-by-point replies to the comments are listed below.

According to the comments of the Referees, the following major changes were made in the paper:

Reviewer #1:

Comments:

This work demonstrates a comprehensive investigation of the phase transition behavior of two new halobismuthates(III) hybrid crystals, coupling with dielectric, ferroelectric, and optical properties. The experimental analysis and conclusions are convincing for appealing to the attention of the related fields. I recommend it for publication but the current version should be revised before the final acceptance.

1. The authors mentioned many structural analogs with phase transition and ferroelectricity have been reported. While I was wondering about the core difference between the current using Azetidine and others. Is there significant new insight for the phase transition mechanism, enhancing physical performance, or something else? Or just using a new amine for new hybrid crystals for ferroelectrics?

Author note:

Numerous factors exist that determine the structure of organic-inorganic hybrids based on halo-Bi(III). These factors are interconnected and include the stoichiometry of the reactants during synthesis as well as the type of the organic part. In crystals, for example, the dimensionality of the anionic sublattices can be moderated by the size of organic cations. Small cations like methyl derivatives of amine or aromatic compounds such as imidazole and pyrrolidine will form mainly 2 or 1-D structures.
On the other hand, large cations (pyridine derivatives, significant aliphatic amines) favour the 0D structures. It seems that ferroelectricity is observed more often in 1D structures than in 0-D. Therefore, we wanted to obtain a 1D anionic structure with R_2MX_5 stoichiometry, where the anionic chains were unfolded in one direction. A relatively small cyclic cation (with dipole moments differs from 0) was embedded between the chains. The dynamics of those cations (during ordering in the low-temperature phases) can generate a polar phase. We followed these assumptions in selecting the amine in the compounds studied.

2. The cis and trans-connected MX6 octahedra in various structural frameworks were highlighted in the portion of the introduction. The definition should be provided for figuring out the classification. Besides, the authors mentioned trans types are rare and exhibit high performance. However, the title hybrids in this work belong to the commonly reported cis type. The introduction section must be properly revised to highlight the purpose of the current work for comparison with others. **Author note:**

As suggested by the Reviewer, the following sentences were added to the introduction section.

In the case of R_2MX_5 stoichiometry, two halide atoms have a bridging position, and four are terminal. Two opposite bridging halide atoms share the vertices of consecutive octahedra, resulting in a linear 1D chain extended in a given direction (*trans*chain). In contrast, in the *cis*-type chain, two adjacent halides join octahedra to form 1D zigzag chains (as shown in Scheme 1).

Reviewer #2:

Exploring lead-free organic-inorganic hybrid ferroelectrics is important for developing environmentfriendly optoelectronic devices. In this manuscript, the authors reported two new lead-free organicinorganic ferroelectrics $(C_3NH_8)_2[BiCl_5]$ and $(C_3NH_8)_2[BiBr_5]$. They exhibit ferroelectricity accompanied by dielectric and second harmonic generation switching behaviors, which have been systematically characterized. I think this work is interesting to the scientific communities of organic-inorganic hybrids and molecular ferroelectrics. Therefore, it is recommended for publication in The Journal of Physical Chemistry Letters after some minor revision.

1. The authors stated that "Both compounds exhibit paraelectric (I) to ferroelectric (II) phase transitions (PTs) at 230/233K and 228/229K, respectively, transitioning from orthorhombic (Pnma) to monoclinic (P21) phases". The phase transition from Pnma (high-temperature phase) to P21 (low-temperature phase) does not belong to the 88 species of paraelectric-ferroelectric phase transitions given by Aizu. Please carefully check the crystal symmetry and give a corresponding explanation. **<u>Author note:</u>**

According to Aizu the PT from orthorhombic - *Pnma* space group tomonoclinic ($P2_1$) is classified as *mmm*F21 species (#54) as partial ferroelectric and ferroelastic transition. This is a partial ferroelectric species becuse the initial point group *mmm* is non-polar and the low tempreture (2) is polar. It admits a spontaneous polarization along the two-fold axis. The species is partial because the orientation states generated by the lost of mirror planes containig the two-fold axis do not change the polarization.

2. In Figure 3, the two phases (Ⅱ phase and Ⅲ phase) of overlayed organic and Bi chains are difficult to distinguish.

<u>Author note:</u>

The figure has been changed to the following:

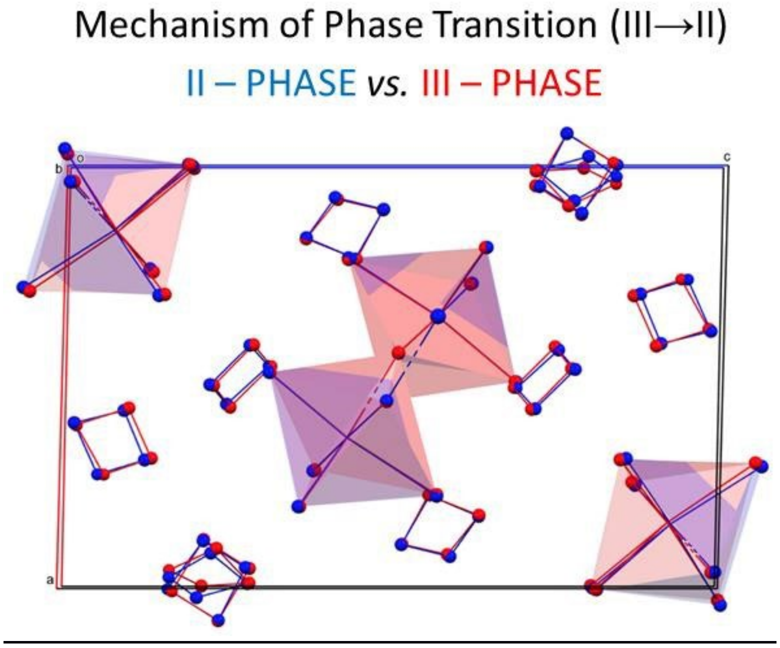

Figure 3. Overlayed organic parts of **ABC** at 200 (blue) and 140 K (red) and inorganic sublattices.

3. The crystal growth process is too brief. More details are needed for others to reproduce the synthesis and crystal growth.

Author note:

The description of the synthesis has been changed to the following.

The materials necessary for the synthesis of $(C_3H_8N)_2[BiCl_5]$ (**ABC**), $(C_3H_8N)_2[BiBr_5]$ (**ABB**) were purchased from commercial sources (Bi_2O_3, 99.999%, azetidine 98%, Sigma-Aldrich) and used without further purification. 2g of Bi_2O_3 was suspended in 50 ml of distilled water, and then HX acid (X: Cl, Br) was added to dissolve the precipitate. 1g of amine was weighed into a separate vessel with 50ml of water, and a few drops of concentrated HX acid were added to this solution. After cooling, the contents were carefully combined with the bismuth salt solution, and after a week, the compound crystallized. An elemental analysis verified the crystals composition: **ABC** C: 14.20% (theor. 14.34%), N: 5.47% (theor 5.58%), H: 3.02% (theor. 3.21%) and **ABB** C: 10.22% (theor. 9.94%), N: 3.89% (theor 3.87%), H: 2.1% (theor. 2.22%). The single crystals suitable for X-ray measurements were grown from an aqueous solution at a constant room temperature. Powder X-ray diffraction verified the phase purity (see Fig. S1). Powder X-ray diffraction was recorded using an X'Pert PRO powder diffractometer operating with Cu Ka radiation

4. In the main text, there is no relevant description of Figures S2 and S4.

Author note:

In the introduction, the sentence below was added.

The powder diffractogram was recorded to confirm the phase compatibility since most physicochemical measurements were performed on the polycrystalline sample (Figure S2, ESI).

In contrast, the reference to Figure S4 has been added to the sentence:

The complex electric permittivity measurements were performed in the 100 – 350 K temperature range and 200 Hz - 2 MHz frequency range (see Figure S4, ESI).

5. Some level B Alerts appear in the checkcif. The authors should try to eliminate these alerts or give a response.

Author note:

In accordance with Reviewer's comments, the alerts have been explained as follows:

_vrf_PLAT196
PROBLEM: No TEMP record and _measurement_temperature .NE. 293 Degree
RESPONSE: The alarm is connected with the lack of the TEMP instruction in the shelx res file. As the positions of hydrogen atoms in the structures are totally constrained it does not influence the final model. Nevertheless, the instruction has been added.

For remaining alarms, the following response has been included in the cif files.

_vrf_PLAT342_BiCl140K
;PROBLEM: Low Bond Precision on C-C Bonds .... 0.06667 Ang.
RESPONSE: The C-C bond precision is low due to the disordered organic part of the structure. Moreover, TWINed structure below phase transition temperature may influence the bond precision as the measured data for the sample are of a lower accuracy.

_vrf_PLAT987_BiCl140K
PROBLEM: The Flack x is >> 0 - Do a BASF/TWIN Refinement Please Check RESPONSE: The refinement was made against merged data (HKLF 4) from TWIN reduction process. The TWIN refinement was performed.

_vrf_PLAT242_BiCl240K
PROBLEM: Low 'MainMol' Ueq as Compared to Neighbors of Bi1 Check
RESPONSE: The false alarm may occur for terminal groups as indicated by checkcif dictionary. Additionally, here in the case of Bi1 atom the disordered ligands are present, which may influence the checkcif test.

_vrf_PLAT342_BiBr200K
PROBLEM: Low Bond Precision on C-C Bonds .... 0.06333 Ang.
RESPONSE: The C-C bond precision is low due to the disordered organic part of the structure. Moreover TWINed structure below phase transition temperature may influence the bond precision as the measured data for the sample are of a lower accuracy.

_vrf_PLAT987_BiBr200K
PROBLEM: The Flack x is >> 0 - Do a BASF/TWIN Refinement Please Check
RESPONSE: The refinement was made against merged data (HKLF 4) from TWIN reduction process. The TWIN refinement was performed.

_vrf_PLAT242_BiBr250K
PROBLEM: Low 'MainMol' Ueq as Compared to Neighbors of Bi1 Check
RESPONSE: The false alarm may occur for terminal groups as indicated by checkcif dictionary.
Additionally, here in the case of Bi1 atom the disordered ligands are present, which may influence the test.

Reviewer #3:

The article presented to me for review presents new and very interesting research results on functional organic-inorganic hybrid materials. I believe that the article will be suitable for publication because of the very minor corrections listed below:

1. In Figure 6a, the electric field strength should be presented in kV/cm units and not V as it is currently. Although the P(E) dependence itself could not be classified as a ferroelectric loop, the inclusion of the I(V) dependence fully represents polarization switching in an electrically lossy material, especially since it

is supported by Figure 9S. Although on page 15, line 19, it is written about the influence of AC conductivity, it seems that at a measuring field frequency of 0.5 Hz, it should rather be about the influence of DC conductivity (similarly on page 16, line 40). **Author note:**

Figure 6a presents two units, V (bottom axis) and kV/cm (top axis). Following the Reviewer's proper remark, the AC abbreviation has been changed to proper sentences.:

……

Figure S9 (ESI) illustrates the polarization-electric field intensity (*P*-*E*) relationship measured on the **ABC** sample in its paraelectric phase, where a considerable contribution of *dc* conductivity is observed.

……

Due to the strong contribution of *dc* conductivity, the sample was polarized in high-temperature phase **I** at 250 K and then cooled down to 180 K …….

According to Figure 6b, a strong *dc* conductivity contribution is observed despite 30 min short-circuiting the sample. Therefore, the background was subtracted from the measured current to extract the peak associated with the change in polarization (insert in Fig 6b).

2. In the case of Figure 2S, in my opinion, the presentation of measured and calculated diffraction patterns should be done in such a way that they can be easily compared. Although the authors have attached a graph of the difference between these two diffraction patterns, I believe that they should be presented one for the other and not on oppositely directed intensity axes.

Author note: Figures have been changed according to the Reviewer's advice.

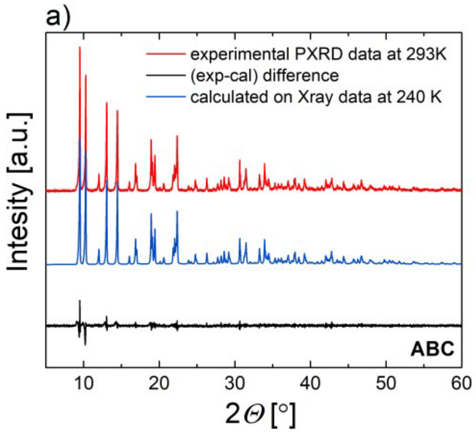

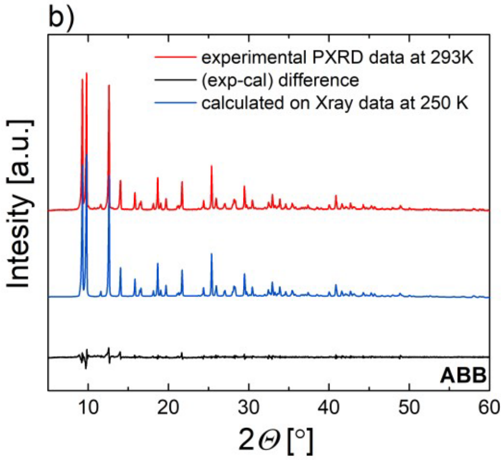

Figure S1. X-ray diffraction pattern of a) **ABC** and **b) ABB** at 293 K (red line) and calculated (blue line) from crystals structure at 250 and 240K for **ABC** and **ABB**, respectively. Black line represents the difference between experimental and calculated data.

3. On page 16, line 52, the authors of the work somewhat exaggeratedly attributed a change in the sign of the pyroelectric current with a change in the sign of the polarizing field. This effect is known in many organic materials and may be related, among others, to charge injection from electrodes and the so-called electret effect. In fact, the only indisputable evidence for the existence of ferroelectric properties is the observation of a ferroelectric hysteresis loop – well-formed and saturated. I recommend the work of M. Maglione Appl. Phys. Lett. 93, 032902 (2008). I think that this radical statement about proving the occurrence of ferroelectricity in the ABB material should be softened. A similar softening will be useful in the final conclusions on page 32, line 10. **Author note:**

We agree with the Reviewer that only the ferroelectric loop is indisputable evidence of the ferroelectric properties of a compound. In the case of ABB, the shape of the loop is characteristic of conductive compounds. Measurement of the pyroelectric current only confirms that polarization is generated, which is reversible with a change in the sign of the applied field. Following this suggestion, we have changed the statement that ABB is ferroelectric to "may" exhibit ferroelectric properties, and the conclusion has also been verified.

Old version:

P-E hysteresis loop and pyroelectric current confirmed the ferroelectric properties for ABC and ABB, with the spontaneous polarization values of 1.66 and 0.8 $mC \cdot cm^{-2}$, respectively.

New version:

P-E hysteresis loop and pyroelectric current confirmed the ferroelectric properties for **ABC** with the spontaneous polarization values of 1.66 $mC \cdot cm^{-2}$. In the case of **ABB**, pyroelectric current measurement also suggests the ferroelectric properties of the crystal.

4. On page S14, there is no information about the rate of temperature change during dielectric measurements and the intensity of the measuring field, and information whether the ferroelectric loops were obtained using a triangular or sinusoidal measuring field. **Author note:**

The missing information concerning the electric experiment has been added to the experimental part.

The complex dielectric permittivity, $\varepsilon^* = \varepsilon' - i\varepsilon''$, measurements were conducted on **ABC** and **ABB** in the form of polycrystalline pellets on an Agilent E4980A Precision LCR Meter between 100 and 350 K in the frequency range between 200 Hz and 2 MHz. The silver electrodes were painted on both opposite sides of the pellets. The overall errors of ε' and ε'' were less than 5%. The temperature was stabilized and controlled using an INSTEC STC200, and the cooling and heating runs were measured with 5K/min ramp. The pyroelectric properties were tested with a Keithley 6517D electrometer/high resistance meter between 180 and 300 K, with a temperature ramp of 2 K min^{-1}. The ferroelectric hysteresis loops were obtained by using a Sawyer–Tower circuit Precision Premier II (Radiant Technologies, Inc.), with the setup of the drive profile type as f =0.5 Hz and standard bipolar signal. The diameter of the pellet was of the order of 5 mm, and its thickness of 1 mm. The surfaces of the polycrystalline pellets were coated with silver conductive paint (Electron Microscopy Sciences, 503).

5. on page S15 the statement that a step change in permittivity is characteristic of orderdisorder transitions is a bit of an exaggeration – in materials such as BaTiO3 or PbZrO3 phase transitions show a step change in permittivity in Tc, but these are not transitions considered to be order-disorder transitions. **Author note:**

The Reviewer is correct, and defining the phase transition mechanism should not be based on the shape of the dielectric anomaly. The conclusion has been drawn based on structural and calorimetric data analysis. Therefore, the following sentence has been changed as below:

Old version

This step-wise change in permittivity at **I→II** PT is characteristic of the order-disorder type transition mechanism. This statement is consistent with the transition entropy values found in the DSC experiment and the X-ray results.

New version

Based on structural analysis and calorimetric, one can conclude that the step-wise change in permittivity at **I→II** PT is characteristic of the order-disorder type transition mechanism.

After making the minor corrections mentioned and softening some of the statements, I fully recommend the article for publication in the Journal of Chemical Physics Letters.

With kindest regards,

Magdalena Rok with co-authors
Department of Ferroics and Semiconductors,
University of Wroclaw, Poland
